# Reassessing Electrolyte Design for Non‐Aqueous Magnesium Batteries: Atomistic Structures and Performance Optimization

**DOI:** 10.1002/adma.202514224

**Published:** 2025-10-30

**Authors:** Hao Xu, Xiaoqian He, Yue Li, Quanquan Guo, Xinyu Sun, Xinlong Xie, Lingyue Liu, Richard M. Laine, Jianxin Zou, Xinliang Feng, Minghao Yu

**Affiliations:** ^1^ National Engineering Research Center of Light Alloy Net Forming & Center of Hydrogen Science School of Materials Science and Engineering Shanghai Jiao Tong University Shanghai 200240 China; ^2^ Center for Advancing Electronics Dresden (cfaed) & Faculty of Chemistry and Food Chemistry Technische Universität Dresden 01062 Dresden Germany; ^3^ Shanghai Key Laboratory of Magnetic Resonance, School of Physics and Electronic Science, Institute of Magnetic Resonance and Molecular Imaging in Medicine East China Normal University Shanghai 200241 China; ^4^ Max Planck Institute of Microstructure Physics 06120 Halle (Saale) Germany; ^5^ Department of Applied Biology and Chemical Technology the Hong Kong Polytechnic University Hong Kong 100872 P. R. China; ^6^ Department of Materials Science and Engineering University of Michigan Ann Arbor MI 48109‐2136 USA

**Keywords:** atomistic structures, chlorine‐containing electrolytes, chlorine‐free electrolytes, electrolyte design, magnesium batteries

## Abstract

Non‐aqueous magnesium (Mg) batteries have attracted considerable attention as a promising next‐generation energy storage technology, owing to the high Mg resource abundance and good safety. Nevertheless, the development of efficient non‐aqueous electrolytes remains a critical challenge that hinders the practical deployment of Mg batteries. This review provides a comprehensive analysis of recent advances, ongoing challenges, and future directions for non‐aqueous electrolytes in Mg batteries, emphasizing both chlorine‐containing and chlorine‐free electrolytes. Furthermore, it discusses how these electrolyte systems influence the Mg stripping/plating process, particularly in terms of compatibility with the Mg metal anode, and evaluates their electrochemical stability, including the applicable voltage window. Chlorine‐containing electrolytes exhibit high ionic conductivities and reversibility but present challenges related to corrosion and limited electrochemical stability. In contrast, chlorine‐free electrolytes offer a more eco‐friendly alternative, though often with lower ionic conductivity and limited compatibility with Mg metal anodes. This review examines electrolyte design strategies, atomistic structures, and performance optimization to outline the advantages and drawbacks of each electrolyte type. Emerging methodologies for advanced electrolyte development are also discussed. This comprehensive analysis highlights the pivotal role of electrolyte innovation in realizing the full potential of non‐aqueous Mg batteries and advancing sustainable, high‐performance energy storage solutions.

## Graphical abstract

This review systematically examines recent advances in non‐aqueous electrolytes for rechargeable magnesium batteries, focusing on chlorine‐containing and chlorine‐free systems. It highlights the interdependent relationship between electrolyte compositions, atomistic structures, and electrochemical performance.

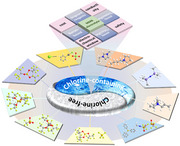

## 1 Introduction

The urgent need for efficient, sustainable, and high‐capacity energy storage solutions has accelerated research into next‐generation battery technologies.^[^ ^1^, ^2^, ^3^, ^4^, ^5^, ^6^, ^7^ ^]^ Non‐aqueous Mg batteries have emerged as a promising alternative to the widely used lithium‐ion batteries (LIBs),^[^ ^8^, ^9^ ^]^ due to the natural abundance of Mg (2.1% in the Earth's crust), high theoretical capacity (3833 mAh cm^−3^ and 2205 mAh g^−1^), and enhanced safety profile due to the near absence of dendritic deposition.^[^ ^10^, ^11^, ^12^, ^13^ ^]^ These attributes make Mg batteries attractive for large‐scale energy storage applications, including renewable energy grids. However, despite these advantages, the practical implementation of Mg batteries is limited by challenges,^[^ ^14^ ^]^ especially the development of suitable electrolytes that can ensure efficient Mg^2+^ transport, high stability, and good compatibility with other cell components.^[^ ^15^, ^16^, ^17^, ^18^ ^]^ Overcoming these challenges is essential to realizing the full potential of Mgbatteries and facilitating their transition from research to practical applications.

Electrolytes function not only as an ion‐conducting medium but also as a critical component in maintaining electrode stability,^[^ ^19^ ^]^ preventing anode surface passivation, and enabling reversible Mg plating/stripping. Unlike monovalent Li^+^,^[^ ^20^, ^21^ ^]^ Mg^2+^ possesses a high charge density, which complicates its transport through electrolyte solutions.^[^ ^22^ ^]^ Furthermore, their divalent nature introduces unique challenges for electrolyte stability,^[^ ^23^ ^]^ including complex interactions with solvents, salts, and electrode interfaces, leading to an increased likelihood of irreversible and parasitic reactions. These issues collectively limit cycle life, efficiency, and reliability, underscoring the need for innovative electrolyte formulations.

To date, a variety of electrolytes have been investigated, including aqueous and non‐aqueous systems.^[^ ^24^, ^25^, ^26^ ^]^ Due to the thermodynamic limitations, it is extremely difficult to match aqueous electrolytes with Mg anodes.^[^ ^27^ ^]^ Non‐aqueous electrolytes have received significant attention due to their tunability, potential for high ionic conductivity (IC),^[^ ^28^ ^]^ as well as good compatibility with metal anodes and cathodes.^[^ ^29^ ^]^ Their versatility allows researchers to optimize key properties, such as dielectric constant (ε), donor number (DN), viscosity, and electrochemical stability windows (ESW).^[^ ^30^, ^31^ ^]^ Additionally, non‐aqueous electrolytes support the integration of a wide range of additives, solvents, and salts, enabling enhancements in ion dissociation kinetics, ionic mobility, passivation mitigation, and overall battery performance.^[^ ^32^ ^]^

Despite their promising features, non‐aqueous electrolytes for Mg batteries have yet to achieve the maturity required for practical applications, primarily due to challenges associated with chlorine‐containing electrolytes and chlorine‐free electrolytes.^[^ ^14^, ^33^ ^]^ Chlorine‐containing electrolytes, typically comprising MgCl_2_ and/or AlCl_3_, have been extensively studied for their favorable electrochemical properties.^[^ ^34^ ^]^ Cl^−^ plays a crucial role in enhancing Mg plating/stripping, thereby improving overall battery efficiency.^[^ ^35^, ^36^, ^37^, ^38^ ^]^ These electrolytes also exhibit high ionic conductivity,^[^ ^39^, ^40^ ^]^ essential for achieving high power densities. However, their corrosive nature can degrade battery components, potentially reducing lifespans. Moreover, environmental and safety concerns associated with chlorinated compounds, especially during recycle, necessitate careful handling and disposal.^[^ ^41^, ^42^, ^43^ ^]^ To address the limitations of chlorine‐containing electrolytes, researchers have developed chlorine‐free alternatives specifically designed to minimize corrosive effects, improving battery durability, longevity, long‐term environmental impact, and ease of handling (recycle).^[^ ^44^ ^]^ Despite their advantages, chlorine‐free electrolytes often demonstrate lower ionic conductivities, leading to reduced power densities and overall performance. Furthermore, the synthesis of effective chlorine‐free electrolytes is frequently more complex and expensive, presenting significant challenges for large‐scale production and commercialization.

In this review, we examine recent progress in the development of both chlorine‐containing and chlorine‐free electrolytes, with particular emphasis on the design of organic solvents, Mg salts, and additives. The discussion highlights innovations in molecular structures and formulation strategies aimed at enhancing electrochemical performance, such as increasing ionic conductivity (typically from <10^−5^ to >10^−3^ S cm^−1^), expanding electrochemical stability windows (ESW, up to 4.0 V vs Mg^2+^/Mg), and improving Coulombic efficiency (CE, often exceeding 95% for optimized systems).^[^ ^14^, ^18^ ^]^ These improvements directly address key challenges, including electrode surface passivation, low ionic conductivity, and poor compatibility with Mg anodes. Besides electrolytes, this review also discusses the fundamentals and design strategies of Mg anodes and cathodes, highlighting their interplay with electrolyte chemistry. By highlighting these emerging areas with concrete performance metrics, this review seeks to inspire further research efforts toward the development of safe and high‐performance non‐aqueous Mg battery electrolytes.

## 2 Basics of Non‐Aqueous Mg Batteries

**Figure 1:**
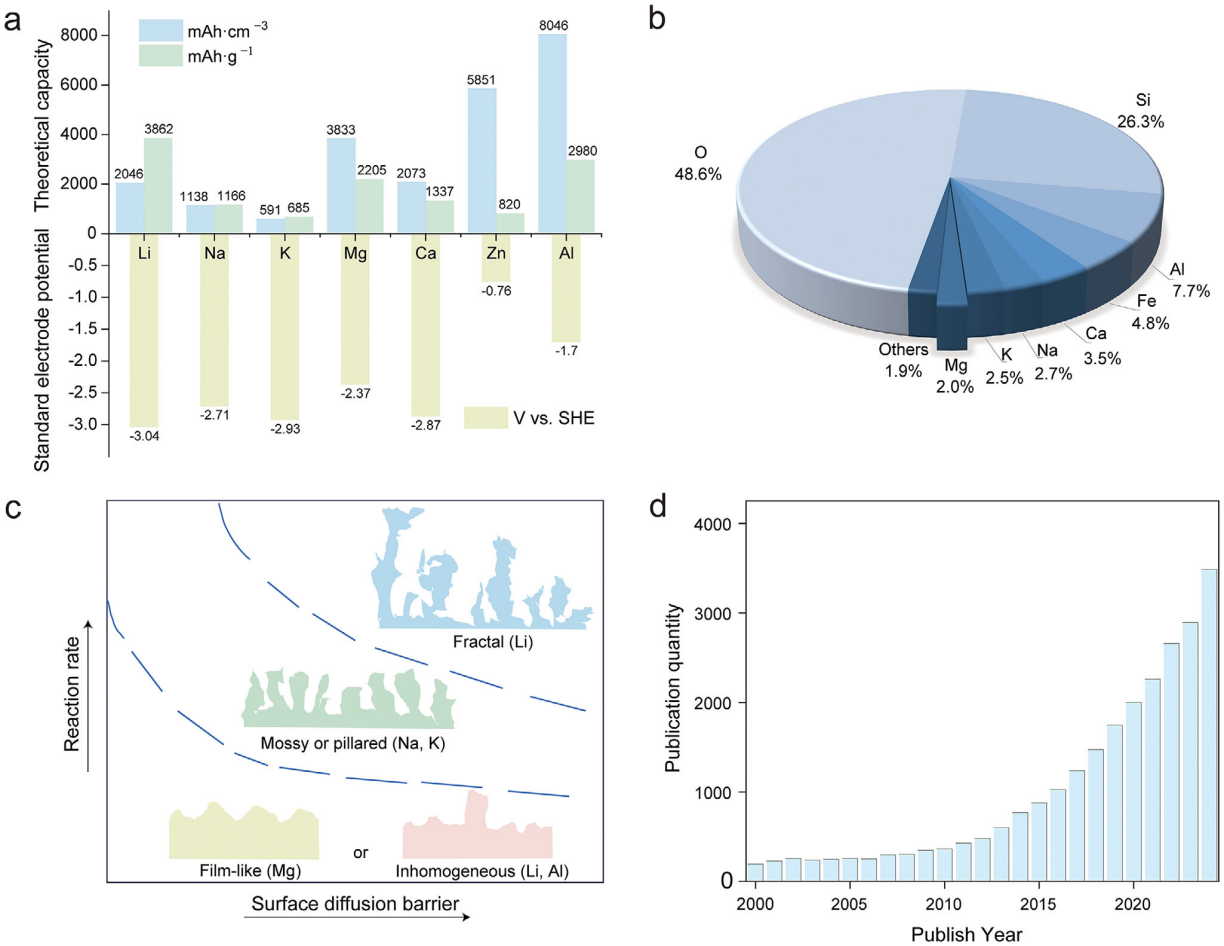
a) Theoretical specific and volumetric capacities, and standard electrode potentials for different metal anodes. b) Elements’ natural abundance in Earth's crust. c) Schematic illustration of electrodeposition patterns for representative metal anodes: Li (fractal and inhomogeneous), Na and K (mossy or pillared), Mg (film‐like), and Al (inhomogeneous). d) Mg battery publication numbers (2000–2024) from Web of Science by searching for the keyword “Mg battery”.

Mg anodes have attracted growing attention as a viable alternative to lithium anodes due to their low dendrite formation tendency and natural abundance. **Figure** **1**a provides a comparative analysis of key parameters influencing the development and potential utility of Mg anodes. The theoretical specific and volumetric capacities, and standard electrode potentials of various metal anodes (Li, Na, K, Mg, Ca, Zn, and Al) are presented. The combination of high volumetric capacity (3832 mAh cm^−3^), moderate specific capacity (2205 mAh g^−1^), and relatively low standard electrode potential (−2.37 V vs SHE) makes Mg particularly attractive for safe, high‐energy‐density rechargeable batteries. Unlike Li, Mg does not easily form dendrites,^[^ ^45^ ^]^ enhancing safety and durability. The relatively high abundance of Mg (2.1%) vs Li (0.0065%) of significance is underscored in Figure 1b for cost‐effective, sustainable resource management and large‐scale deployment for next‐generation batteries.^[^ ^46^ ^]^

As shown in Figure 1c, distinct differences in the electrodeposition morphologies of various metal anodes are evident. Li and Na typically exhibit dendritic or mossy/pillared deposition due to the inhomogeneous nucleation associated with the high surface diffusion barriers and rapid deposition kinetics. K shows similar mossy or pillared growth, arising from its weak metallic bonding and low surface energy, which promotes loose and unstable deposits. Al often presents irregular and less‐defined morphologies, largely influenced by substrate interaction and high overpotentials. In contrast, Mg generally shows film‐like and uniform deposition, reflecting its strong metallic bonding and suppressed dendrite formation. Notably, the smooth and compact morphology of Mg greatly mitigates the risk of short circuits and enhances the operational safety of Mg‐based batteries. Furthermore, stable Mg deposition behavior contributes to the long‐term structural integrity of Mg electrodes, which is essential for achieving durable cycling performance of Mg batteries. Complementing these observations, the temporal evolution of publication volume, presented in Figure 1d, demonstrates a clear upward trajectory in “Mg battery” related research from 2002 to 2024. This surge in academic interest underscores the increasing recognition of strategic value for Mg in the development of sustainable and high‐performance energy storage technologies.

### 2.1 Battery Configuration and Principle

In Mg batteries, discharge releases Mg^2+^ from an Mg anode by oxidation, which then migrates through the electrolyte to the cathode, and is inserted in the cathode.^[^ ^47^, ^48^ ^]^ This process generates a flow of electrons that provides electrical power through an external circuit. When charging, an external power source drives the reverse process, causing Mg^2+^ to migrate back to the anode, where they are reduced and deposited.^[^ ^49^ ^]^ The electrochemical performance of Mg batteries is heavily influenced by the electrolyte, which must support efficient Mg^2+^ transport while remaining stable during cycling. In addition, cathode reactions in non‐aqueous Mg batteries fall into three main categories: Mg^2+^‐, hybrid ion‐dominated, and anion‐involved reactions.

**Figure 2:**
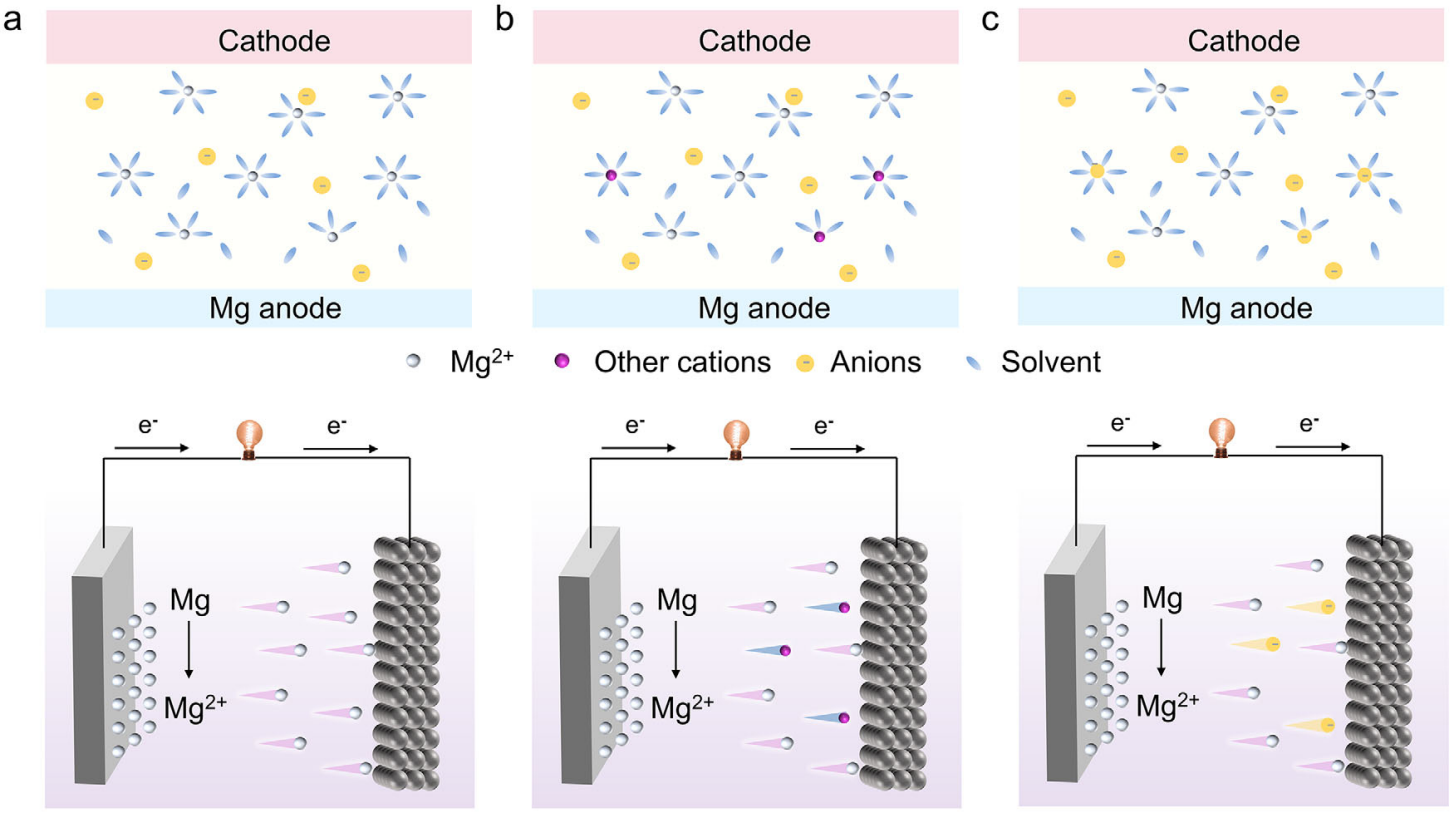
Schematic of rechargeable Mg batteries during discharge with (a) Mg^2+^‐, (b) dual ions‐dominated, and (c) anion‐involved cathode reactions.

Mg^2+^‐dominant cathode reactions are the most common, where Mg^2+^ directly participates in the electrochemical process (**Figure** **2**a). The advantage of Mg^2+^‐dominated cathodes is their simplicity and direct involvement of Mg in both charge and discharge processes. However, this system is often limited by the poor kinetics of Mg^2+^ insertion.^[^ ^50^ ^]^ Hybrid ion‐dominated cathode reactions offer a more complex scenario, where both Mg^2+^ and other co‐insertion ions (e.g., Li^+^, Na^+^, K^+^) participate in the cathode reactions (Figure 2b). In this system, the cathode active material accommodates both Mg^2+^ and other cationic species present in the electrolyte, significantly enhancing electrochemical performance by increasing the number of charge carriers. Furthermore, this system can reduce the strain typically encountered in pure Mg^2+^‐dominated cathodes, thereby improving battery cycle life and efficiency. While dual ion‐dominated cathode reactions offer enhanced performance, they also introduce additional complexities. These include the need to precisely control the concentrations of solvated ions and electrolyte design to effectively deliver multiple ions to the cathode without inducing unwanted parasitic side reactions.

Anion‐involved cathode reactions represent a distinct class of reactions,^[^ ^51^, ^52^, ^53^ ^]^ where electrolyte anions play a dominant role in cathode electrochemical processes (Figure 2c). In this system, the primary reaction involves the insertion of anions from electrolytes to the cathode, either in association with Mg^2+^ or independently. Anion‐involved systems have garnered interest due to their ability to exploit the chemical stability of certain anions, offering high‐energy density reactions without the need to insert significant amounts of Mg^2+^. In dual‐ion systems, the participation of electrolyte‐derived anions in the charge compensation process inevitably leads to electrolyte consumption. Moreover, the major challenges include ensuring electrochemical reversibility and the stability of the anions under repeated cycling, which can complicate the development of suitable electrolytes and cathode materials. Accordingly, the three categories of cathode reactions involve distinct ionic species, thus requiring electrolytes tailored to accommodate different types of charge carriers.

### 2.2 Fundamental Mg Anode Chemistries and Anode Design

**Figure 3:**
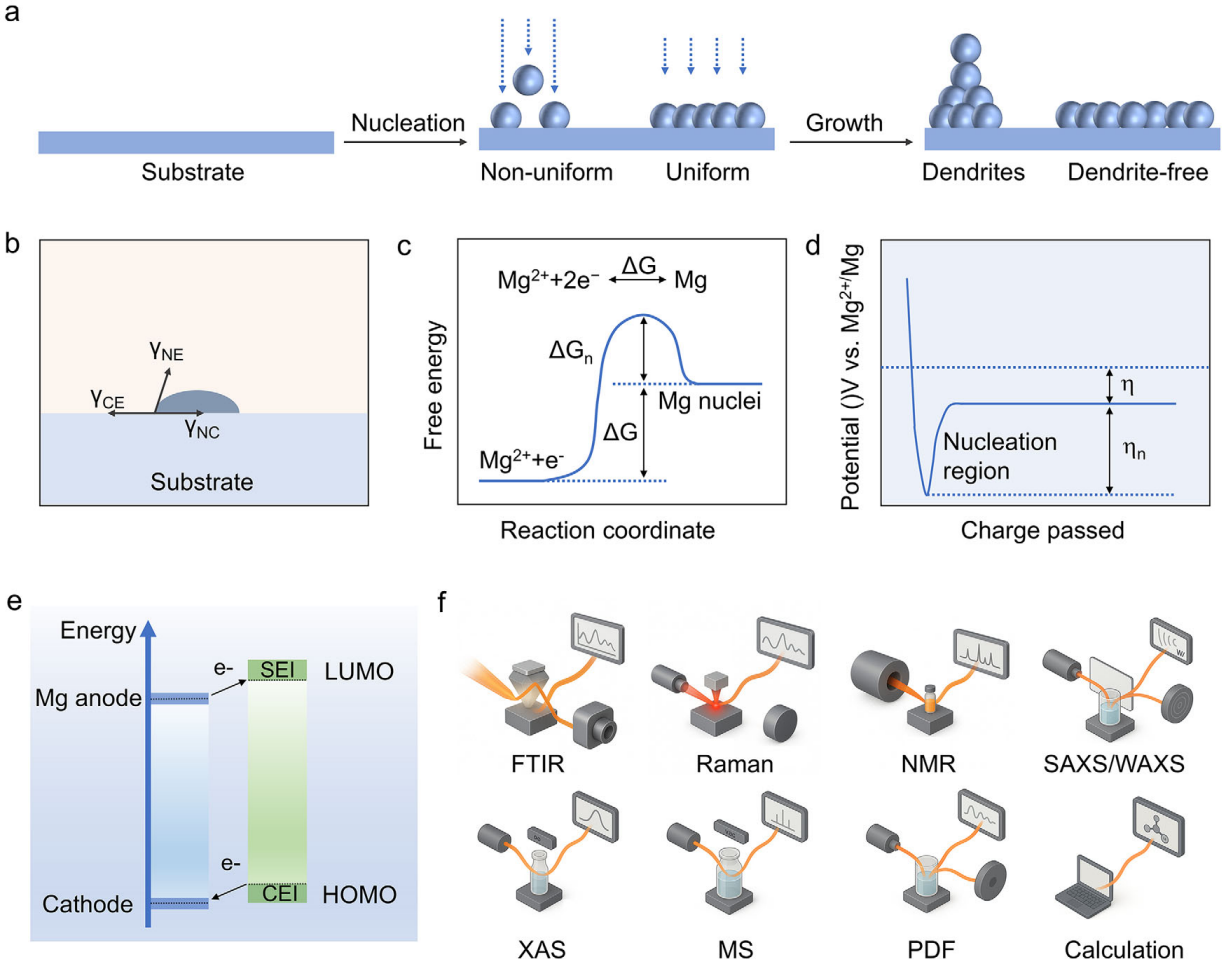
a) The Mg nucleation and growth processes on collectors. b) The Mg nucleation on collectors based on the classical heterogeneous nucleation model. c) Schematic plot of free energy. d) Plot of the typical voltage profiles of galvanostatic Mg deposition. e) The illustration of SEI formation mechanism based on molecular orbital energy levels. f) Characterization techniques for investigating electrolyte components and molecular structures.

During stripping and plating, Mg^2+^ undergoes strong coulombic interactions with both solvent molecules and anions, resulting in sluggish desolvation kinetics and increased energy barriers for charge transfer at the electrode‐electrolyte interface.^[^ ^54^ ^]^ At the microscopic level, Mg deposition proceeds via a series of elementary steps, including desolvation of Mg^2+^, electron transfer to form neutral Mg atoms, surface diffusion, and nucleation into clusters. The strong Mg–Mg metallic bonds and high nucleation barriers significantly suppress dendritic growth, contributing to smooth deposit morphologies under ideal conditions (**Figure** **3**a).^[^ ^55^ ^]^ However, recent studies have revealed that nonuniform nucleation can still occur, particularly under high current densities.^[^ ^56^ ^]^

The initial stage of Mg deposition onto current collectors involves a nucleation process, which can be described by classical heterogeneous nucleation theory.^[^ ^57^, ^58^ ^]^ As depicted in Figure 3b, this model conceptualizes deposited nuclei as spherical caps on the substrate.^[^ ^59^ ^]^ Thermodynamically, nucleation proceeds through a competition between the decrease in bulk free energy associated with the phase transition and the increase in surface free energy arising from the creation of new interfaces. The bulk free energy reduction serves as the driving force for nucleation, whereas the surface energy penalty establishes an energetic barrier that must be overcome. Consequently, a nucleation‐free energy barrier and corresponding overpotential are typically observed during the early stages of deposition (Figure 3c,d), reflecting the inherent kinetic resistance to nucleation.^[^ ^60^ ^]^ The overall Mg electrodeposition process proceeds with a negative Gibbs free energy change. It should be emphasized that Figure 3c is intended to schematically illustrate the nucleation process rather than the bulk deposition equilibrium. During nucleation, the total free energy includes both the volume free energy (ΔG_v, favorable) and the surface energy penalty (ΔG_s, unfavorable). As a result, the free energy of a small Mg nucleus can initially be higher than that of Mg^2+^, leading to a nucleation barrier. Only when the nucleus size exceeds the critical radius, the total ΔG becomes negative and metallic Mg growth become thermodynamically favorable.^[^ ^57^ ^]^

Upon formation of thermodynamically and kinetically stable nuclei, subsequent growth ensues at a relatively constant rate until the final morphology is achieved. Within the framework of heterogeneous nucleation theory, the total interfacial energy is determined by a balance among the interfacial tensions between the nucleus, the electrolyte, and the current collector, denoted as *γ*_NE_, *γ*_CE_, and *γ*_NC_, respectively, with θ representing the contact angle at the triple phase boundary (Equation 1).^[^ ^59^ ^]^ Based on this model, the heterogeneous nucleation barrier (ΔG_het_) can be related to the homogeneous nucleation barrier (ΔG_hom_) through a geometric factor S(θ), as shown in Equations (2 and 3). Since S(θ) decreases monotonically with decreasing contact angle, lowering θ effectively reduces the nucleation barrier. In the ideal case where θ approaches 0°, nucleation becomes barrierless, whereas when θ reaches 180°, the energy barrier is maximized. These insights suggest that enhancing the magnesiophilicity of current collector surfaces offers an effective strategy to facilitate Mg nucleation by minimizing the nucleation energy barrier.^[^ ^61^ ^]^

(1) $$\cos\theta =\frac{\gamma_{\mathrm{CE}}-\gamma_{\mathrm{NC}}}{\gamma_{\mathrm{NE}}}$$

(2) $$\Delta G_{\mathrm{het}}=S\left(\theta \right)\Delta G_{\mathrm{hom}}$$

(3) $$S\left(\theta \right)=\frac{\left(2+\cos\theta \right){\left(1-\cos\theta \right)}^{2}}{4}$$

Solid electrolyte interphase (SEI) formation on Mg anodes remains a complex and controversial topic, which is intrinsically linked to the Lowest Unoccupied Molecular Orbital (LUMO) energy levels of the electrolyte components (Figure 3e). Electrolytes with low‐lying LUMOs are more susceptible to reductive decomposition at the anode surface, facilitating SEI formation. Unlike in Li‐based systems, where SEI layers form spontaneously and are crucial for stabilizing the interface, classical SEI layers are rarely observed on Mg due to the limited reductive decomposition of suitable electrolytes. Nonetheless, decomposition of electrolytes at low potentials can generate interfacial films composed primarily of inorganic species such as MgCl_2_, MgO, and MgCO_3_.^[^ ^28^ ^]^ These layers are often thin and heterogeneous, offering limited ionic conductivity for Mg^2+^, which contributes to interfacial resistance growth over cycling. Engineering artificial SEI layers via electrolyte additives, molecular interlayers, or surface pretreatments is an emerging strategy to improve anode stability. Overall, despite the relatively uniform deposition behavior of Mg, challenges remain in controlling interfacial chemistry, minimizing overpotentials, and achieving long‐term cycling stability.

**Figure 4:**
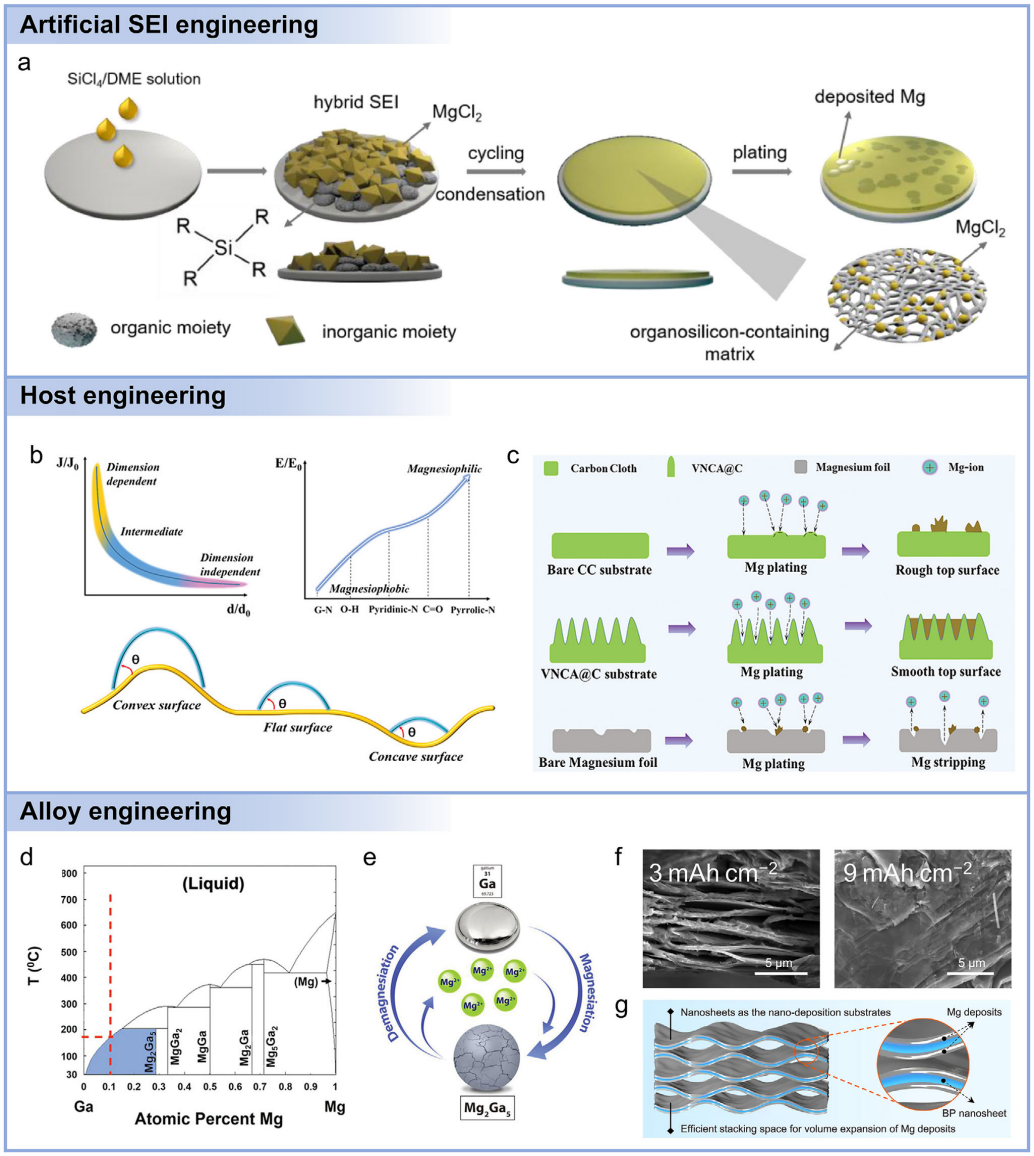
a) Schematic diagrams of SEI evolution and Mg deposition manner based on Mg–Si electrode in conventional electrolyte. Reproduced with permission.^[^ ^62^ ^]^ Copyright 2022, Elsevier. b) Schematic illustration of the design matrix for uniform Mg electrodeposition. c) The schematic illustration of Mg electrodepositing on varied substrates. Reproduced with permission.^[^ ^63^ ^]^ Copyright 2021, WileyVCH. d) Equilibrium phase diagram of the Mg–Ga system and e) Schematic of the magnesiation and demagnesiation processes during which liquid Ga is reversibly converted into solid Mg_2_Ga_5_ at constant temperature and pressure. Reproduced with permission.^[^ ^64^ ^]^ Copyright 2019, WileyVCH. f) Cross‐section morphologies of Mg@BP composite negative electrode with plated capacity of 3 and 9 mAh cm^−2^, and g) schematic illustration of Mg plating behavior of Mg@BP composite negative electrode. Reproduced with permission.^[^ ^65^ ^]^ Copyright 2024, Springer Nature.

Although Mg anodes inherently exhibit reduced dendritic growth compared with Li, their practical application remains challenged by sluggish desolvation/transport kinetics, unstable interphases, and limited reversibility. Recent advances have therefore converged on several major design strategies.^[^ ^66^, ^67^ ^]^ For example, artificial SEI engineering has emerged as an effective approach to suppress parasitic reactions and enable facile Mg^2+^ transport.^[^ ^68^ ^]^ In this strategy, ex situ SEI is pre‐constructed on the Mg surface before cell assembly, offering tunability of thickness and composition. As a representative example, Li et al. designed a bilayer MgCl_2_/organosilicon coating via simple SiCl_4_/DME treatment (**Figure** **4**a).^[^ ^62^ ^]^ Unlike the bare Mg anode, where spontaneous reactions with the electrolyte generated a dense passivation layer (MgO, MgF_2_, and other Mg compounds) with high Mg^2+^ diffusion barriers, the pre‐structured hybrid SEI exhibited an anti‐passivation effect. This heterogeneous SEI was formed through the reactions between Mg and the SiCl_4_/DME solution at room temperature, consisting of robust inorganic components (e.g., MgCl_2_ in the upper layer) and flexible organic moieties (e.g., organosilicon in the lower layer). During cycling, the pristine SEI became uniform due to Si–O–Si condensation and the dynamic redistribution of MgCl_2_, ultimately evolving into a Si‐rich organic matrix embedded with MgCl_2_ nanodomains. This hierarchical structure, characterized by its low diffusion barrier, facilitated smooth, dendrite‐free Mg deposition with low overpotential. More advanced designs include mixed ion–electron conducting (MIEC) perovskite layers,^[^ ^69^ ^]^ defect‐free MOF coatings,^[^ ^70^ ^]^ and polymer‐based SEIs,^[^ ^68^ ^]^ which enhance ionic transport and block solvent penetration. Apart from ex situ SEI, in situ SEI can be formed and engineered by introducing proper additives or salts into the electrolyte. For instance, Mai et al. incorporated Mg(pftb)_2_ (Mg salt of perfluoro‐tert‐butanol) into the magnesium–aluminum chloride complex (MACC) electrolyte,^[^ ^71^ ^]^ extending the electrolyte oxidative stability to ≈4 V vs Mg^2+^/Mg. A compact solid–electrolyte interphase (SEI) with a thickness of ≈10.5 nm was distinctly detected on the Mg anode surface. Structural characterization indicated that this SEI, formed through electrolyte decomposition during cycling, consisted of an organic‐inorganic hybrid layer that effectively protected the Mg anode. With this electrolyte, a symmetric Mg cell achieved stable cycling for over 2000 h at 0.5 mA cm^−2^, maintaining a low overpotential of ≈50 mV. Other strategies for stabilizing Mg anodes include the introduction of iodine into Mg(TFSI)_2_/DME to generate a MgI_2_‐rich interphase that suppresses surface passivation and reduces overpotentials,^[^ ^72^ ^]^ the utilization of GeCl_4_ to construct a self‐healing Ge–MgCl_2_ SEI that enables long‐term cycling even at high current densities,^[^ ^73^ ^]^ and the application of SO_2_ gas to convert inactive MgO into MgSO_3_, thereby enhancing ionic transport.^[^ ^74^ ^]^ However, in situ SEIs often suffer from uncontrollable thickness and composition, while excess additives may affect electrode performance.

Mg host engineering provides a complementary manner to regulate Mg nucleation and growth by modifying the collector surfaces.^[^ ^75^, ^76^ ^]^ 3D host architectures with porous skeletons and high surface areas can effectively reduce local current densities and Mg^2+^ nucleation barriers, thereby suppressing dendritic growth and stabilizing long‐term cycling.^[^ ^77^ ^]^ Their robust frameworks and internal pores also accommodate electrode volume changes during repeated Mg plating/stripping. As summarized in Figure 4b, uniform Mg electrodeposition is governed by electric field, chemisorption, and geometric confinement.^[^ ^63^ ^]^ Short nanoarrays (<10 µm) homogenize current density, avoiding tip‐induced nonuniformity. Carbon‐based scaffolds have been widely used due to their high conductivity, large surface area, and structural stability. Their N/O functional groups could greatly enhance magnesiophilicity and reduce nucleation barriers, while concave microchannels lowering activation energy for Mg nucleation. For instance, graphitic carbon nano‐substrates (GC‐NSs) enabled uniform Mg nucleation and long cycling for over 1000 cycles with a high CE of 99.9% at 0.2 mA cm^−2^.^[^ ^78^ ^]^ N,O‐doped carbon nanofiber arrays (VNCA@C) shown in Figure 4c further improved magnesiophilicity and delivered stable operation even at 10 mA cm^−2^.^[^ ^63^ ^]^ Besides, incorporating magnesiophilic nanoparticles such as Au or Ag into porous carbon or Cu frameworks could lower nucleation overpotentials and ensure homogeneous Mg deposition, as demonstrated by Au@PCNF,^[^ ^79^ ^]^ Ni(OH)_2_@CC,^[^ ^80^ ^]^ and Ag@3D Cu mesh.^[^ ^81^ ^]^ These designs achieved stable cycling at both high current densities and large areal capacities (cycled for over 600 h at 10mA cm^−2^@1 h). Other advanced strategies include Zn skeletons with MgO coatings that improve interfacial charge transport,^[^ ^82^ ^]^ and 3D Mg_3_Bi_2_ scaffolds that combine high surface area with intrinsic Mg affinity for durable cycling in Mg(TFSI)_2_/DME electrolytes.^[^ ^83^ ^]^ Overall, Mg host design through porous architectures, dopant engineering, and magnesiophilic interfaces, has proven highly effective in stabilizing Mg deposition, offering a promising pathway toward reliable and high‐performance rechargeable Mg batteries.

Alloy‐type anode design represents another effective strategy to address the intrinsically sluggish Mg^2+^ diffusion and high nucleation overpotential of pure Mg metal.^[^ ^84^ ^]^ Mg‐based alloy anodes with post‐transition metals (Ga, In, and Sn) have been explored as alternatives to pure Mg anodes.^[^ ^85^ ^]^ In particular, Ga was shown to form multiple Mg–Ga intermetallics (Figure 4d,e). Mg_2_Ga_5_ demonstrated long cycle life (>1000 cycles) and high rate performance due to its self‐healing solid–liquid phase transformation, delivering a specific capacity ≈290 mAh g^−1^ after 680 cycles at 615 mA g^−1^.^[^ ^64^ ^]^ In alloys exhibited reversible Mg–In reactions at low potentials, but poor kinetics and cycling stability limited their application.^[^ ^86^ ^]^ Group IVA elements are of particular interest due to their high theoretical capacities. Si and Ge are predicted to form Mg_2_Si and Mg_2_Ge phases with ultrahigh capacities, but unfavorable defect formation energies and sluggish Mg diffusion hinder practical implementation.^[^ ^87^, ^88^ ^]^ Amorphous or doped nanostructures could alleviate these barriers. In addition, Sn has been actively studied. Although Mg_2_Sn formation enables high capacity, large volume expansion, sluggish Mg^2+^ kinetics, and interfacial instability cause pulverization, poor reversibility, and rapid fading.^[^ ^89^, ^90^ ^]^ Hooman et al. prepared Mg_2_Sn material by melting quantified amounts of Mg and Sn powder.^[^ ^91^ ^]^ The resulting ingot was crushed and milled using a high‐energy ball mill. The obtained micron‐sized Mg_2_Sn material, when used as an anode for Mg batteries, fragmented into nano‐sized Sn particles during the initial discharging and charging processes, leading to improved performance in subsequent magnesium intercalation and de‐intercalation processes. Moreover, strategies such as nanostructuring and the use of composites (e.g., Mg_2_Sn/graphite) have demonstrated improved performance.^[^ ^91^, ^92^ ^]^

Group VA elements (P, Bi, and Sb) and their alloys have also been investigated as potential Mg‐based anodes. Among them, black phosphorus (BP) shows ultrahigh theoretical capacity through Mg alloying, but large structural instability and bond breaking remain critical challenges. Zhao et al. developed a Mg@BP composite anode (Figure 4f,g),^[^ ^65^ ^]^ where Mg^2+^ partially intercalates into black phosphorus before metallic Mg deposition, enabling uniform Mg plating and reduced dead Mg formation. The symmetric cells cycled stably for 1600 h with a cumulative capacity of 3200 mAh cm^−2^, and maintained nearly 100% CE at 16 mA cm^−2^ with a plating capacity of 16 mAh cm^−2^. Bi is a promising anode material for rechargeable Mg batteries due to its high Mg^2+^ mobility and favorable alloying reaction with Mg to form Mg_3_Bi_2_. Bi–Mg alloys offer a theoretical specific capacity of 385 mAh g^−1^.^[^ ^84^ ^]^ Experimentally, nanocrystalline Bi–Mg anodes achieve >250 mAh g^−1^ at 7.7 A g^−1^ and were compatible with common Mg electrolytes,^[^ ^93^ ^]^ making Bi an attractive choice for Mg alloy anodes. Sb shares the rhombohedral crystal structure similar to Bi. Besides, both the Sb‐Mg phase diagram and the Bi‐Mg phase diagram contain similar intermetallic compounds (Mg_3_Sb_2_ and Mg_3_Bi_2_). Timothy and co‐workers reported micro‐ and nano‐Sb films as anodes,^[^ ^84^ ^]^ which exhibited low capacity (6 mAh g^−1^ at 660 mA g^−1^). Besides, Matsui et al. recently found that Mg_3_Sb_2_ alloy anodes exhibited minimal electrochemical activity, indicating that Mg_3_Sb_2_ was likely an unsuitable candidate for Mg batteries.^[^ ^94^ ^]^

In summary, the rational design of Mg anodes requires a multi‐dimensional strategy that integrates interphase stabilization, alloy engineering, and host modification. While each approach individually addresses specific bottlenecks, their synergistic integration, combined with electrolyte optimization, is essential to achieve both thermodynamic stability and kinetic accessibility. Future progress will rely on coupling these strategies with interfacial analyses, advanced in situ/operando characterizations, and theoretical modelings to clarify the fundamental mechanisms of Mg nucleation and growth.

### 2.3 Fundamental Cathode Chemistries and Cathode Design

Cathode development for Mg batteries remains one of the most formidable challenges, owing to the sluggish solid‐state diffusion of divalent Mg^2+^, interfacial incompatibility with electrolytes, and structural instabilities under repeated cycling. To address these issues, multiple classes of cathode materials including intercalation‐type compounds,^[^ ^95^, ^96^, ^97^ ^]^ conversion‐type compounds,^[^ ^98^, ^99^ ^]^ and organic compounds have been extensively investigated, each requiring distinct design strategies to balance capacity, reversibility, and long‐term stability.^[^ ^100^ ^]^

**Figure 5:**
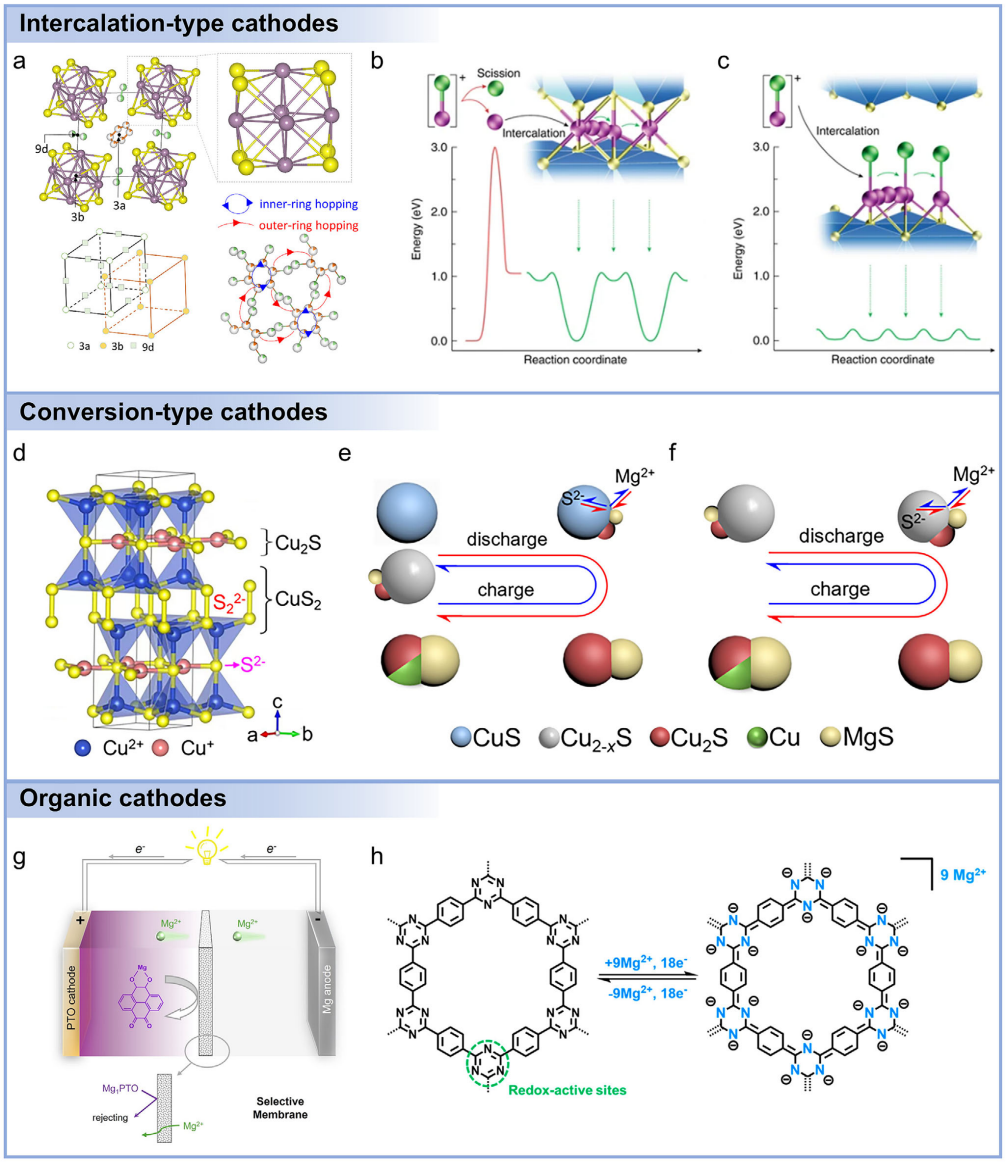
a) Crystalline structure and sublattices in Chevrel phase Mo_6_S_8_: Mo_6_S_8_ superanion and positions of highly symmetric 3a, 3b, and 9d sites (viewed along the^[^ ^273^ ^]^ direction), sublattice of 3a and 3b sites, and outer‐ring and inner‐ring hopping between partially occupied inner and outer sites. Mo is colored light purple, S yellow, the inner site orange and white, and the outer site green and white. Reproduced with permission.^[^ ^95^ ^]^ Copyright 2017, American Chemical Society. Energy diagrams for the intercalation and diffusion of Mg^2+^ and MgCl^+^. b) Typical intercalation of Mg^2+^ involves scission of MgCl^+^ ions into Mg^2+^ and Cl^−^, which requires a substantial activation energy of 3 eV at least. Subsequent diffusion of divalent Mg^2+^ also has a high‐migration energy barrier of 16 eV, which results in the limited level of intercalation at room temperature. c) Intercalation of MgCl^+^ bypasses the sluggish scission of the Mg–Cl bond at the electrolyte–cathode interface; afterward MgCl^+^ diffuses fast in the expanded interlayers due to the fairly low‐migration energy barrier of 0.18 eV. Mg and Cl atoms are shown as purple and green spheres, respectively. Reproduced with permission.^[^ ^101^ ^]^ Copyright 2017, Springer Nature. d) Structure of covellite CuS. e) Schematic diagram for the conversion of CuS to Cu_2_S/Cu and concomitant heteroepitaxial growth of MgS (discharge) and the back‐conversion of Cu_2_S/Cu to Cu_2‐_ *_x_* S (charge) at the first cycle. f) Schematic diagram for the conversion of Cu_2‐_ *_x_* S to Cu_2_S/Cu and concomitant heteroepitaxial growth of MgS (discharge) and the back‐conversion of Cu_2_S/Cu to Cu_2‐_ *_x_* S (charge) at the following cycles. Reproduced with permission,^[^ ^102^ ^]^ Copyright 2024, Elsevier g) Schematic of Mg‐PTO cells and the function of the Separator and Membrane. Selective membranes are employed to address this issue by preventing the diffusion of the Mg_1_PTO intermediate. Reproduced with permission.^[^ ^103^ ^]^ Copyright 2025, American Chemical Society. h) Chemical structure and possible electrochemical redox mechanism of the COF. Reproduced with permission.^[^ ^104^ ^]^ Copyright 2020, American Chemical Society.

For intercalation‐type cathodes, including spinel oxides,^[^ ^105^, ^106^ ^]^ Chevrel phases,^[^ ^95^ ^]^ and layered sulfides,^[^ ^107^, ^108^, ^109^ ^]^ the bottleneck lies in the intrinsically slow Mg^2+^ diffusion through compact lattices. The Chevrel phase (CP) Mo_6_S_8_ material is the first intercalation‐type material with reversible Mg^2+^ insertion and extraction discovered by the Aurbach group.^[^ ^39^, ^110^, ^111^ ^]^ As shown in **Figure** **5**a, its structure consists of Mo_6_ octahedra at the face‐centered positions and S atoms at the cube corners, forming a quasi‐simple cubic stacking.^[^ ^95^ ^]^ The 3a (internal) and 9d (external) sites provide 3D migration channels, exhibiting two discharge plateaus at 1.2 and 1.1 V, though electrostatic repulsion restricts occupancy to one Mg^2+^ per site type. Two diffusion modes are also illustrated: inner‐ring hopping within hexagons and outer‐ring hopping between adjacent hexagons. Typically, Mo_6_S_8_ cathodes deliver reversible capacities above 90 mAh g^−1^. Beyond CPs, layered transition metal sulfides such as VS_4_,^[^ ^112^ ^]^ VS_2_,^[^ ^109^ ^]^ TiS_2_,^[^ ^113^ ^]^ MoS_2_,^[^ ^114^ ^]^ and WS_2_ have also been studied. For example, Zhao et al. added 1‐butyl‐1‐methylpiperidinium chloride (PP_14_Cl) to the APC electrolyte and utilized VS_2_ nanosheets as the cathode material.^[^ ^115^ ^]^ The assembled battery exhibited a high initial discharge capacity of 460 mAh g^−1^ at 20 mA g^−1^ and a reversible specific capacity of 200 mAh g^−1^ after 300 cycles at 1000 mA g^−1^. Yoo et al. reported an expanded TiS_2_ material capable of storing MgCl^+^ cations.^[^ ^101^ ^]^ As depicted in Figure 5b, the cleavage energy of the Mg‐Cl bond in the electrolyte reaches up to 3 eV, while the diffusion barrier for individual Mg^2+^ in TiS_2_ is 1.06 eV, imposing a limitation on the Mg^2+^ storage capacity of TiS_2_ material at room temperature. When the distance of crystal planes for TiS_2_ material increases, the diffusion barrier for MgCl^+^ in TiS_2_ is reduced to 0.18 eV (Figure 5c). Subsequently, the authors employed 1‐butyl‐1‐methylpyrrolidinium chloride (PY_14_Cl) as an electrolyte additive. After initial activation, TiS_2_ cathodes could accommodate PY_14_^+^ ions, causing the lattice expansion. At 25°C, the expanded TiS_2_ exhibited a specific capacity of 239 mAh g^−1^ at 24 mA g^−1^. Furthermore, Liu et al. synthesized a sandwich‐like MoS_2_@C microsphere composite material,^[^ ^116^ ^]^ which delivered a high initial discharge specific capacity of 213 mAh g^−1^ at 50 mA g^−1^. After 50 cycles, the battery still maintained a specific capacity of 84 mAh g^−1^. In addition, WS_2_ theoretically possesses a high discharge voltage (1.5 V vs Mg^2+^/Mg) and a high theoretical specific capacity of 360 mAh g^−1^ (based on the discharge product Mg_2_WS_2_).^[^ ^117^ ^]^ However, a study by Latha et al. indicated that WS_2_‐based composite material exhibited a low actual discharge specific capacity of 82 mAh g^−1^,^[^ ^118^ ^]^ showing a certain gap compared to the theoretical value.

Compared with metal sulfides, metal oxides generally deliver higher operating voltages due to strong metal–oxygen bonds. Among them, layered α‐V_2_O_5_ has been extensively studied. Yoo et al. revealed that single layered α‐V_2_O_5_ could reversibly insert and extract one mol Mg^2+^ at 110°C,^[^ ^97^ ^]^ maintaining a high specific capacity exceeding 280 mAh g^−1^. Despite their potential for high energy density, oxides often suffer from poor kinetics at room temperature. In contrast, transition metal selenides exhibit lower Mg^2+^ diffusion barriers and higher electronic conductivity. Mao et al. first experimentally demonstrated that TiSe_2_ and VSe_2_ exhibited better electrochemical performance compared to TiS_2_ and VS_2_.^[^ ^119^ ^]^ At 25°C, both TiSe_2_ and VSe_2_ cathodes in the APC electrolyte exhibited a high initial discharge specific capacity of 130 mAh g^−1^ and stable cycling over 40 cycles. Liu et al. fabricated self‐supporting electrodes composed of WSe_2_ nanowires,^[^ ^120^ ^]^ maintaining a specific capacity of 200 mAh g^−1^ after 160 cycles at 50 mA g^−1^.

Beyond layered structures, spinel‐type chalcogenides and oxides represent another important class of cathode materials. Sulfide spinels such as Ti_2_S_4_ and Zr_2_S_4_ show reversible Mg^2+^ insertion with moderate diffusion barriers, whereas many oxide spinels (e.g., Mn_2_O_4_, Cr_2_O_4_) suffer from sluggish Mg^2+^ transport, limiting their reversibility unless nanostructured. Sun et al., for the first time, reported Ti_2_S_4_ as a sulfide spinel‐type cathode material for Mg batteries.^[^ ^121^ ^]^ Studies on spinel‐type oxides have gradually attracted great attention. Relevant calculations indicate a high Mg^2+^ diffusion barrier (600 to 800 meV) in spinel‐type oxides,^[^ ^105^ ^]^ such as Mn_2_O_4_, Cr_2_O_4_, Ni_2_O_4_, and Co_2_O_4_. In fact, spinel‐type Mn_2_O_4_ exhibits a very low Mg^2+^ insertion level (less than 3 at% Mg per mole of Mn_2_O_4_).^[^ ^122^, ^123^ ^]^ Due to the sluggish Mg^2+^ diffusion, only spinel‐type materials with nanoscale dimensions and porous structures can manifest noticeable Mg^2+^ insertion and extraction behaviors.^[^ ^124^, ^125^ ^]^

Olivine‐structured materials are also considered promising cathode material candidates. The migration barrier of Mg^2+^ in FePO_4_ is 580 meV,^[^ ^126^ ^]^ while the migration barrier of Mg^2+^ in Mg_0.5_FePO_4_ is 1025 meV, indicating that the extraction of Mg^2+^ from Mg_0.5_FePO_4_ is highly challenging. NuLi et al. showed that MgMnSiO_4_ cathode exhibited a reversible specific capacity of 253.8 mAh g^−1^ at 15.7 mA g^−1^.^[^ ^127^ ^]^ Orikasa et al. utilized a method of ion exchanging to fabricate the olivine‐type MgFeSiO_4_ material,^[^ ^128^ ^]^ which exhibited a reversible specific capacity exceeding 300 mAh g^−1^ at 100°C, with an average discharge voltage of 2.4 V. In general, olivine‐type structured materials are environmentally friendly and cost‐effective.

Conversion‐type cathodes, including chalcogenides, sulfur, selenium, and iodine, have drawn great attention in Mg batteries due to their high theoretical capacities and diverse redox chemistries.^[^ ^129^, ^130^, ^131^ ^]^ Nevertheless, these materials often suffer from large volume changes, sluggish kinetics, and intermediate dissolution, which limit their practical applications. Chalcogenides (e.g., sulfides, selenides) represent one of the most intensively studied categories because of their tunable electronic structures and morphologies.^[^ ^132^, ^133^, ^134^, ^135^ ^]^ As shown in Figure 5d–f, CuS undergoes transformation into Cu_2−x_S (such as Cu_2_S and Cu_1.94_S) phases, eventually releasing metallic Cu.^[^ ^136^, ^137^ ^]^ Its reversibility follows the Hard–Soft Acid–Base principle,^[^ ^138^ ^]^ where soft S^2−^ anions interact preferentially with Cu⁺ rather than Mg^2+^, thus allowing regeneration of Cu_2_S from MgS. To mitigate volume change and improve kinetics, nanoscale engineering has been employed. Hierarchical CuS “microflowers” with expanded interlayers maintained structural integrity over long‐term cycling, while CuS quantum dots embedded in carbon nanorods delivered 324 mAh g^−1^ after 100 cycles.^[^ ^139^ ^]^ Beyond sulfides, other selenides such as Cu_2−_ *_x_* Se,^[^ ^129^ ^]^ Ag_2_Se,^[^ ^140^ ^]^ and CoSe_2_ have also been investigated,^[^ ^141^ ^]^ showing similar conversion behaviors.

As an abundant and cheap conversion‐type material, sulfur has gained even greater attention due to its exceptionally high theoretical capacity (1675 mAh g^−1^, 3200 Wh L^−1^).^[^ ^142^, ^143^ ^]^ However, magnesium–sulfur batteries face substantial obstacles such as polysulfide dissolution, shuttle effects, and sluggish Mg^2+^ kinetics.^[^ ^144^ ^]^ Considerable progress has been achieved by designing non‐nucleophilic electrolytes, such as Mg(HMDS)_2_–AlCl_3_‐based electrolytes.^[^ ^145^ ^]^ At the same time, conductive hosts (e.g., mesoporous carbon CMK‐3, reduced graphene oxide composites) have been developed to confine active species and enhance cycling stability.^[^ ^26^ ^]^ Despite these advances, reversible capacities and lifetimes vary widely depending on sulfur loading, host design, and electrolyte formulation. Selenium provides an alternative with higher electronic conductivity and similar conversion chemistry.^[^ ^146^ ^]^ However, their lower theoretical capacity (670 mAh g^−1^) and limited natural abundance constrain large‐scale application. In addition to chalcogens, iodine has emerged as another promising conversion‐type cathode option. Elemental iodine undergoes a fast, reversible two‐electron redox reaction between I_3_^−^ and I^−^, offering low cost and a high specific capacity. Using Mg(HMDS)_2_/tetraglyme electrolytes, Mg–I_2_ batteries achieved a specific capacity of 140 mAh g^−1^.^[^ ^147^ ^]^ Yet most studies use very low iodine loadings (≈1 mg cm^−2^), which minimizes but does not eliminate shuttle effects and Mg anode passivation. At higher loadings, these issues are expected to worsen, underscoring the need for host confinement strategies or interfacial engineering to stabilize polyiodide intermediates. Taken together, conversion‐type cathodes present unique opportunities and challenges for Mg batteries. Chalcogenides showcase tunable structures and morphologies, sulfur offers unmatched capacity but suffers from polysulfide instability, selenium provides improved conductivity yet limited sustainability, and iodine features rapid kinetics but faces shuttle and passivation issues. Future development will rely on synergistic advances in morphology control, electrolyte design, and interfacial stabilization, aiming to balance high energy density with long‐term cycling stability.

Organic cathodes are gaining significant attention as alternatives to conventional inorganic cathodes in Mg batteries.^[^ ^148^ ^]^ Their advantages stem from structural tunability, sustainability, and the ability to accommodate multivalent Mg^2+^ through flexible redox‐active sites with weaker electrostatic interactions compared to inorganic compounds. Current researches have primarily focused on small organic molecules, conjugated polymers, and covalent organic frameworks (COFs), each offering unique opportunities and challenges. Small organic molecules are attractive due to their well‐defined redox centers and relatively high theoretical capacities. Quinone derivatives, in particular, have been extensively studied. For example, 2,5‐dimethoxy‐1,4‐benzoquinone (DMBQ) delivered 260 mAh g^−1^ in Mg(ClO_4_)_2_/γ‐butyrolactone electrolyte, demonstrating over 50% of its theoretical value.^[^ ^149^ ^]^ To address dissolution issues, large conjugated molecules have been investigated, such as vat dyes and pyrene derivatives. Debashis et al. showed that Vat Orange 11 exhibited long‐term cycling stability (≈1000 cycles) with reversible carbonyl/enolate conversion,^[^ ^150^ ^]^ while pyrene‐4,5,9,10‐tetraone (PTO) demonstrated a heterogeneous liquid–solid conversion mechanism and outstanding rate capability, achieving 210 mAh g^−1^ even at 20.4 A g^−1^ with specific power up to 30 kW kg^−1^.^[^ ^148^ ^]^ Ren et al. further demonstrated that the electrochemical performance of PTO was improved by using fluorine‐free magnesiated sulfonated poly(ether ether ketone) (Mg‐SPEEK) selective membranes, addressing the Mg_1_PTO dissolution in electrolytes (Figure 5g).^[^ ^103^ ^]^ Beyond quinones, rhodizonate salts such as Li_2_C_6_O_6_ and Na_2_C_6_O_6_ have emerged as high‐capacity candidates (theoretical capacity >500 mAh g^−1^).^[^ ^151^, ^152^, ^153^, ^154^, ^155^, ^156^ ^]^ Tian et al. demonstrated that the Mg||Na_2_C_6_O_6_ cell retained a capacity of 200 mAh g^−1^ even at a high current density of 1 A g^−1^,^[^ ^157^ ^]^ maintaining a reversible capacity of 125 mAh g^−1^ after 600 cycles. The output energy and power densities of 525 Wh kg^−1^ and 4490 W kg^−1^ surpassed those of conventional high‐voltage inorganic cathodes.

Polymeric organics could link redox‐active units into macromolecular frameworks, which reduces solubility and improves structural integrity. Conjugated polymers such as poly(anthraquinonyl sulfide) (PAQS) and polyimides provide multiple carbonyl/imide groups with electronic pathways for Mg^2+^ storage. Liao et al. used 1,4‐polyanthraquinone (14PAQ),^[^ ^158^ ^]^ finding capacity retention up to 99% after 100 cycles in Mg(HMDS)_2_‐based electrolytes, far outperforming PAQS (30%). Beyond carbonyl polymers, recent advances have expanded into anion‐storage polymers. Zhao‐Karger and colleagues developed a polytriphenylamine (PTPAn)/graphene composite capable of reversible [B(hfip)_4_]^−^ anion storage,^[^ ^159^ ^]^ delivering ≈105 mAh g^−1^ with energy density near 300 Wh kg^−1^, representing one of the highest voltages (≈3 V) achieved in Mg batteries. Sulfur‐containing polymers, such as poly‐2,2′‐dithiodianiline (PDTDA) and sulfurized polyacrylonitrile (SPAN), have also been explored, offering enhanced conductivity and higher redox potentials.^[^ ^160^ ^]^ Although capacities (≈70–120 mAh g^−1^) and cycling stability remain limited, these results illustrate the versatility of polymer chemistry in tailoring electrochemical properties. Overall, polymerization provides a powerful route to address solubility and stability issues of small molecules, while offering design flexibility for voltage and capacity optimization.

Covalent organic frameworks (COFs) represent a new class of crystalline, porous polymeric cathodes that combine molecular tunability with long‐range structural order. Their periodic channels facilitate ion transport while preventing dissolution. As shown in Figure 5h, Wang et al. demonstrated a triazine‐COF synthesized from 1,4‐dicyanobenzene, which delivered ultralong cycling stability (3000 cycles) with a decay rate as low as 0.0196% per cycle.^[^ ^104^ ^]^ The electrode achieved energy density of 146 Wh kg^−1^ and power density of 2.8 kW kg^−1^, with pseudocapacitive behavior enabling fast Mg^2+^ kinetics. By incorporating redox‐active motifs such as carbonyls, imines, or azo groups directly into the framework, COFs provide interconnected ion–electron pathways, balancing stability with high performance. Their modular chemistry also offers opportunities to integrate conductive π‐conjugated linkers or heteroatoms, further enhancing Mg^2+^ accessibility and reversibility. Overall, organic cathodes, from small molecules to polymers and COFs, hold great promise for overcoming the intrinsic challenges of Mg^2+^ storage. Continued efforts in molecular engineering, structural optimization, and electrolyte compatibility are crucial to realize their full potential in high‐energy and sustainable Mg batteries.

Beyond the intrinsic properties of cathode materials, the electrolyte plays a crucial role in interfacial stability through the formation of a cathode electrolyte interphase (CEI)_._^[^ ^57^ ^]^ Oxidative decomposition of electrolyte components at high voltages leads to CEI formation, which must simultaneously provide Mg^2+^ permeability and electronic insulation. The composition and morphology of the CEI are highly sensitive to the Highest Occupied Molecular Orbital (HOMO) energy levels of electrolyte components (Figure 3e). Accordingly, rational electrolyte design of high‐voltage‐stable solvents and tailored anions has become a key strategy to stabilize cathode interfaces and enable durable cycling performance.

In summary, advancing Mg cathodes demands a multi‐dimensional and integrated design philosophy that simultaneously optimizes lattice structures, reaction mechanisms, and interfacial chemistry. Intercalation, conversion, and organic cathodes each present unique challenges, yet also provide complementary insights into Mg^2+^ storage mechanisms. Future progress will rely on coupling materials innovation with electrolyte and interphase engineering, as well as leveraging in situ/operando characterization and theoretical modeling to capture the dynamic processes that govern Mg insertion and conversion reactions.

### 2.4 Fundamental Characterizations of Electrolytes

Comprehensive characterization of electrolytes requires the integration of spectroscopic, structural, and computational approaches to unravel solvation structures, ion transport, and interfacial chemistries (Figure 3f). Vibrational spectroscopies, including Fourier‐transform‐infrared‐spectroscopy (FTIR) and Raman spectroscopy, are fundamental for probing solvation structures and ion–solvent interactions through characteristic molecular vibrations.^[^ ^12^, ^161^ ^]^ Nuclear magnetic resonance (NMR) spectroscopy offers atomistic resolution of coordination environments, ion pairing, and dynamic exchange processes, while pulsed‐field gradient NMR provides direct measurements of ion diffusion coefficients.^[^ ^31^, ^162^ ^]^ UV–vis spectroscopy sensitively monitors electrolyte stability and the evolution of transient redox intermediates. Mass spectrometry (MS) complements these techniques by detecting volatile species, mapping degradation pathways, and tracking gas evolution during electrochemical reactions.^[^ ^6^ ^]^

To probe local and mesoscale structures inaccessible to conventional lab sources, synchrotron‐based X‐ray characterizations provide unparalleled opportunities.^[^ ^163^, ^164^ ^]^ Synchrotron radiation, generated from relativistic electrons in storage rings, delivers orders‐of‐magnitude higher flux, continuous tunability from soft to hard X‐rays, and high brilliance,^[^ ^12^, ^165^ ^]^ enabling element‐specific and time‐resolved studies. X‐ray absorption spectroscopy (XAS), including X‐ray absorption near edge structure (XANES) and Extended X‐ray Absorption Fine Structure (EXAFS), reveals oxidation states, electronic configurations, and coordination structures of Mg and other relevant species during cycling. X‐ray scattering techniques,^[^ ^166^ ^]^ such as wide‐angle X‐ray scattering (WAXS), small‐angle X‐ray scattering (SAXS), and pair distribution function (PDF) analysis,^[^ ^167^ ^]^ elucidate electrolyte nanostructures, solvation aggregates, and mesoscale heterogeneity. Synchrotron‐based photoelectron spectroscopies (X‐ray photoelectron spectroscopy, XPS) provide surface‐sensitive information on CEI and SEI chemistry, while synchrotron X‐ray microscopy and tomography allow visualization of deposit morphologies and 3D electrode architectures. Unique advantages of synchrotron‐based techniques include i) elemental specificity for disentangling multi‐component systems, ii) time resolution down to milliseconds for capturing transient species, and iii) operando compatibility for probing electrochemical interfaces under realistic cycling conditions. Nonetheless, careful experimental considerations are critical: beam‐sensitive electrolytes require minimized exposure or flow cells to mitigate radiolysis; path lengths must be optimized to avoid self‐absorption; detection modes (transmission, fluorescence, or electron yield) must be chosen based on concentration and matrix; and inert‐atmosphere handling is essential to prevent contamination.

Theoretical and computational methods complement experimental characterizations by offering molecular‐level understanding and predictive capability.^[^ ^168^, ^169^ ^]^ Density functional theory (DFT) calculations estimate electrolyte stability through analysis of HOMO–LUMO levels, electrostatic potential (ESP) distributions, and charge localization. Molecular dynamics (MD) simulations capture the dynamic solvation structure, ion transport pathways, and interfacial phenomena with temporal resolution beyond experimental reach. The synergy of multi‐modal spectroscopy, synchrotron‐based analysis, and theory‐guided modeling provides a holistic and quantitative framework for understanding electrolyte properties and electrolyte–electrode interfacial chemistry, which is indispensable for guiding rational electrolyte design in Mg batteries.

**Figure 6:**
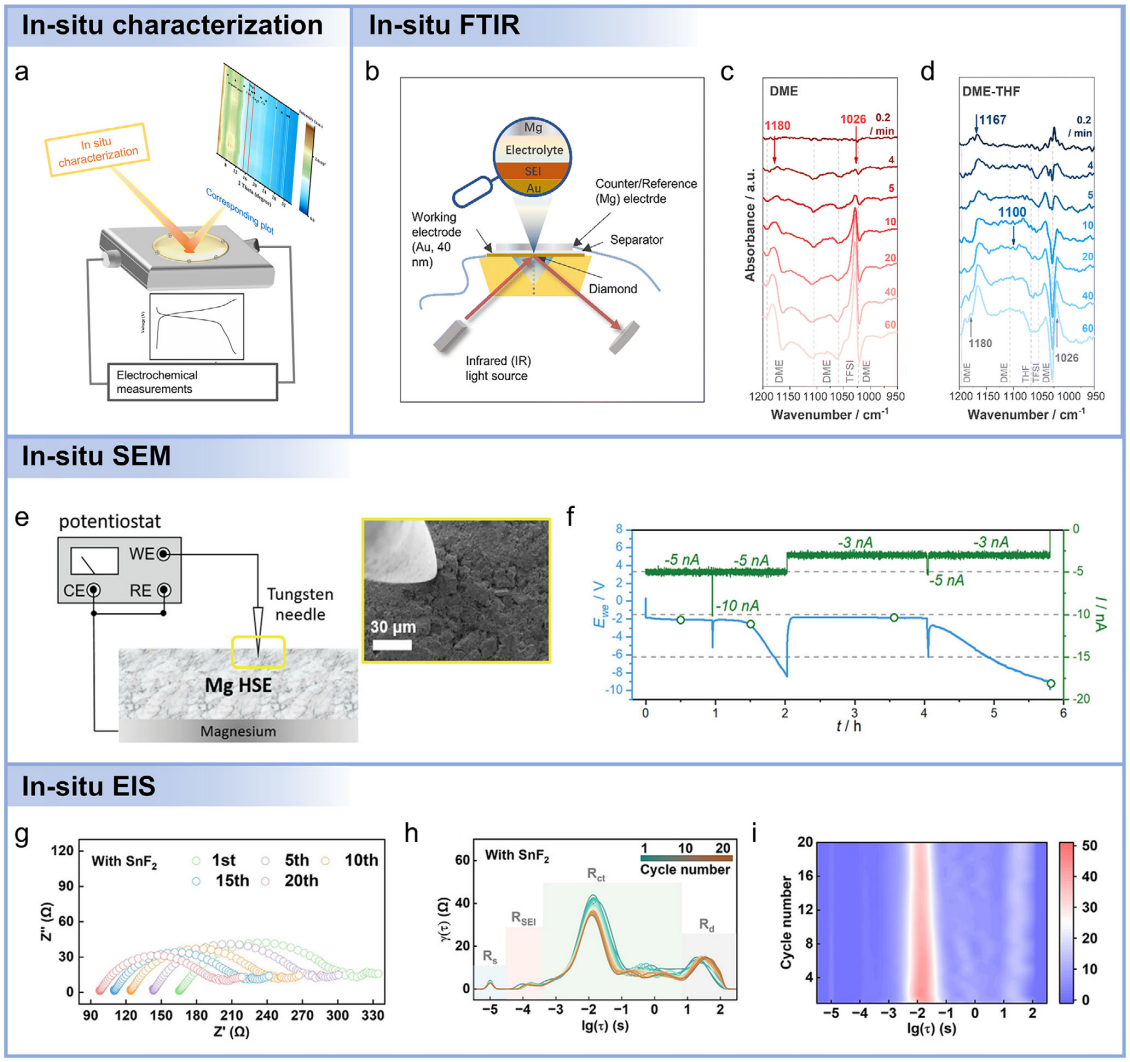
a) Illustration of in situ detection techniques. b) Cell configuration for in situ FTIR test. In situ FTIR spectra of Mg deposited from c) DME and d) DME‐THF electrolytes under 0.25 mA cm^−2^. Reproduced with permission.^[^ ^171^ ^]^ Copyright 2023, Royal Society of Chemistry. e) Schematic illustration for the setup of in situ SEM, adapted based on previous work. The inset shows the initial morphology of MgSP‐HSE. f) Potential profile with different applied currents during the Mg deposition. Reproduced with permission.^[^ ^172^ ^]^ Copyright 2023, WileyVCH. g) In situ EIS and h,i) corresponding DRT plots of Mg//Mg cells in the Mg(OTf)_2_+SnF_2_/MOPA electrolytes during 20 plating/stripping cycles. Reproduced with permission.^[^ ^173^ ^]^ Copyright 2025, WileyVCH.

Beyond the ex situ characterizations, in situ and operando counterparts provide critical advantages, as shown in **Figure** **6**a. Specifically, in situ measurements preserve the relevant electrolyte/electrode environment during controlled perturbations.^[^ ^58^ ^]^ Operando methods track dynamic changes synchronously under realistic cycling conditions, thus enabling correlation of spectral features with instantaneous electrochemical states.^[^ ^170^ ^]^ Reliable in situ/operando experiments demand careful cell design (optical or X‐ray transparent windows, minimized path length), synchronization with electrochemical signals, mitigation of beam/laser‐induced damage, and cross‐validation with complementary techniques and theoretical modeling to ensure reliability.^[^ ^170^ ^]^ While these methodological insights establish the capabilities and limitations of in situ and operando techniques, it is equally important to illustrate how such approaches have been applied to address specific challenges in magnesium electrochemistry. By examining representative studies, one can demonstrate how careful selection of electrolyte systems, measurement conditions, and spectroscopic methods enables direct observation of SEI formation, intermediate species, and dynamic interfacial processes.

For instance, Sun et al. designed a hybrid‐solvent electrolyte by dispersing a non‐nucleophilic DME solvent in a non‐fluorinated and weakly coordinating solvent (THF) to prevent DME decomposition and promote the formation of a stable SEI.^[^ ^171^ ^]^ In situ FTIR spectroscopy (Figure 6b) was employed to monitor the evolution of electrolyte decomposition products and surface film composition during Mg electrodeposition in real time. FTIR spectra were collected at 0.25 mA cm^−2^ using a 40 nm Au thin‐film working electrode, with pre‐deposition spectra serving as the reference background. This approach enabled the identification of both newly formed species (positive absorption peaks) and consumed or desorbed species (negative peaks). In the DME‐based electrolyte (Figure 6c), FTIR spectra revealed the rapid formation of Mg(OCH_3_)_2_, indicated by characteristic peaks at 1026 and 1180 cm^−1^ after only 4 min of deposition, signifying ether C–O bond cleavage. Simultaneously, negative peaks at 1023, 1060, 1105, and 1192 cm^−1^ corresponded to DME and TFSI^−^ consumption, confirming their involvement in parasitic reactions. An extended deposition (60 min) intensified these signals, indicating continued accumulation of decomposition products. Upon the introduction of THF (Figure 6d), in situ FTIR detected significant suppression of Mg(OCH_3_)_2_ formation and the appearance of peaks at 1167 and 1100 cm^−1^, characteristic of poly(tetrahydrofuran) (PTHF) formed via THF ring‐opening polymerization. These findings demonstrated that THF contributed directly to SEI formation, generating a more uniform polymeric surface layer that stabilized the interface.

While in situ FTIR provides molecular‐level insight into chemical transformations at the electrolyte/electrode interface, it does not directly capture the morphological evolution of Mg metal during deposition. To complement these spectroscopic observations, advanced imaging techniques such as in situ electrochemical Scanning Electron Microscopy (SEM) are necessary to visualize real‐time structural changes at the Mg‐electrolyte interface. By integrating chemical and morphological analyses, one can achieve a comprehensive understanding of both interfacial chemistry and deposition dynamics, enabling correlation of SEI formation with macroscopic growth behavior. As illustrated in Figure 6e, Wei et al. developed an in situ SEM electrochemical cell comprising a free‐standing and isostatically pressed Mg_0.5_Sn_2_(PO_4_)_3_‐ hybrid solid electrolyte (MSP‐HSE) pellet (≈1 mm thick, 1 cm diameter) coupled to a polished Mg foil serving as both Mg reservoir and reference electrode. A tungsten microneedle acted as the working electrode and current collector due to its chemical stability against Mg. This configuration enabled localized Mg deposition at the microneedle tip under controlled conditions, with SEM imaging capturing the deposition process. Galvanostatic deposition at −5 nA required an overpotential of ≈−1.9 V, attributed to constriction resistance at the small contact area and the associated iR drop. The overpotential increased steadily, reaching ≈−8.5 V after 2 h, consistent with gradual contact loss at the anodic interface (Figure 6f). SEM images revealed that Mg nucleation occurred at the contact spot and expanded laterally in a planar, conformal fashion, progressively filling cracks and surface irregularities. This in situ SEM methodology thus provided direct correlation between electrochemical parameters (current, overpotential) and morphological evolution, with the observation of planar, crack‐filling growth behavior at room temperature, suggesting that dendrite‐free Mg deposition can be achieved under optimized interfacial conditions.

To extract quantitative information regarding interfacial resistance and charge‐transfer kinetics, in situ Electrochemical Impedance Spectroscopy (EIS) can be utilized to monitor real‐time changes in interfacial properties throughout cycling. By combining morphological imaging with impedance measurements, one can achieve a more comprehensive perspective on how the structure and composition of the interphase affect Mg plating and stripping, effectively linking observed growth patterns with corresponding electrochemical behavior. For example, Xiao et al. developed a dual‐functional SnF_2_ additive for chlorine‐free Mg(OTf)_2_ electrolytes to enhance Mg plating/stripping performance.^[^ ^173^ ^]^ To elucidate interfacial evolution, they performed in situ EIS measurements and analyzed the data using the distribution of relaxation times (DRT) method (Figure 6g). DRT offers a model‐free approach that transforms impedance spectra from the frequency to the time‐constant domain, enabling clear separation of coincident electrochemical events. As shown in Figure 6h, four distinct time regions were identified, including bulk resistance (R_s_, ≈10^−5^ s), SEI resistance (R_SEI_, ≈10^−4^ s), charge‐transfer resistance (R_ct_, 10^−3^‐10^0^ s), and diffusion‐related impedance (R_d_, 10^1^–10^2^ s). In the SnF_2_‐containing electrolyte, both R_SEI_ and R_ct_ peaks were markedly lower than those in the blank electrolyte, indicating improved Mg^2+^ transport and faster interfacial charge‐transfer kinetics. Continued cycling further decreased these impedance contributions, signifying progressive interphase optimization (Figure 6i). Conversely, the blank electrolyte exhibited slightly increased SEI impedance, consistent with Mg surface corrosion and passivation. These findings confirm that SnF_2_ enables the in situ formation of a conductive and Sn‐rich hybrid SEI, suppressing parasitic reactions and facilitating kinetically favorable, reversible Mg plating/stripping.

Collectively, these in situ and operando studies highlight the power of complementary techniques to elucidate Mg‐electrolyte interfacial behaviors. Integrating these approaches provides a comprehensive understanding of the chemical, structural, and electrochemical processes governing Mg plating and stripping. The combination of multi‐modal in situ characterization with advanced modeling and high‐throughput experimentation enables rational design of electrolytes and artificial SEI layers. Expanding the repertoire of in situ techniques is expected to accelerate the development of stable and dendrite dendrite‐free Mg metal anodes with improved cycling performance. However, current studies are still limited in scope, and more diverse in situ and operando techniques are needed to fully capture the dynamic and multi‐scale processes at the Mg‐electrolyte interface.

In summary, Mg batteries present unique challenges due to the intrinsic physicochemical properties of Mg^2+^, such as its high charge density, strong solvation, and slow interfacial desolvation kinetics. Effective strategies to address these challenges involve designing electrolytes that not only provide robust electrochemical stability but also optimize solvation structures, ion–solvent interactions, and interfacial chemistries. This approach underpins the rational selection and engineering of electrolyte components, including solvents, salts, and functional additives. Complementary characterization of both the Mg anode and cathode, as well as the electrolyte and interphase, provides essential insights into the mechanisms of Mg plating/stripping, intercalation processes, and interfacial evolution, guiding the development of high‐performance Mg batteries.

## 3 Key Electrolyte Components for Non‐Aqueous Mg Batteries

**Figure 7:**
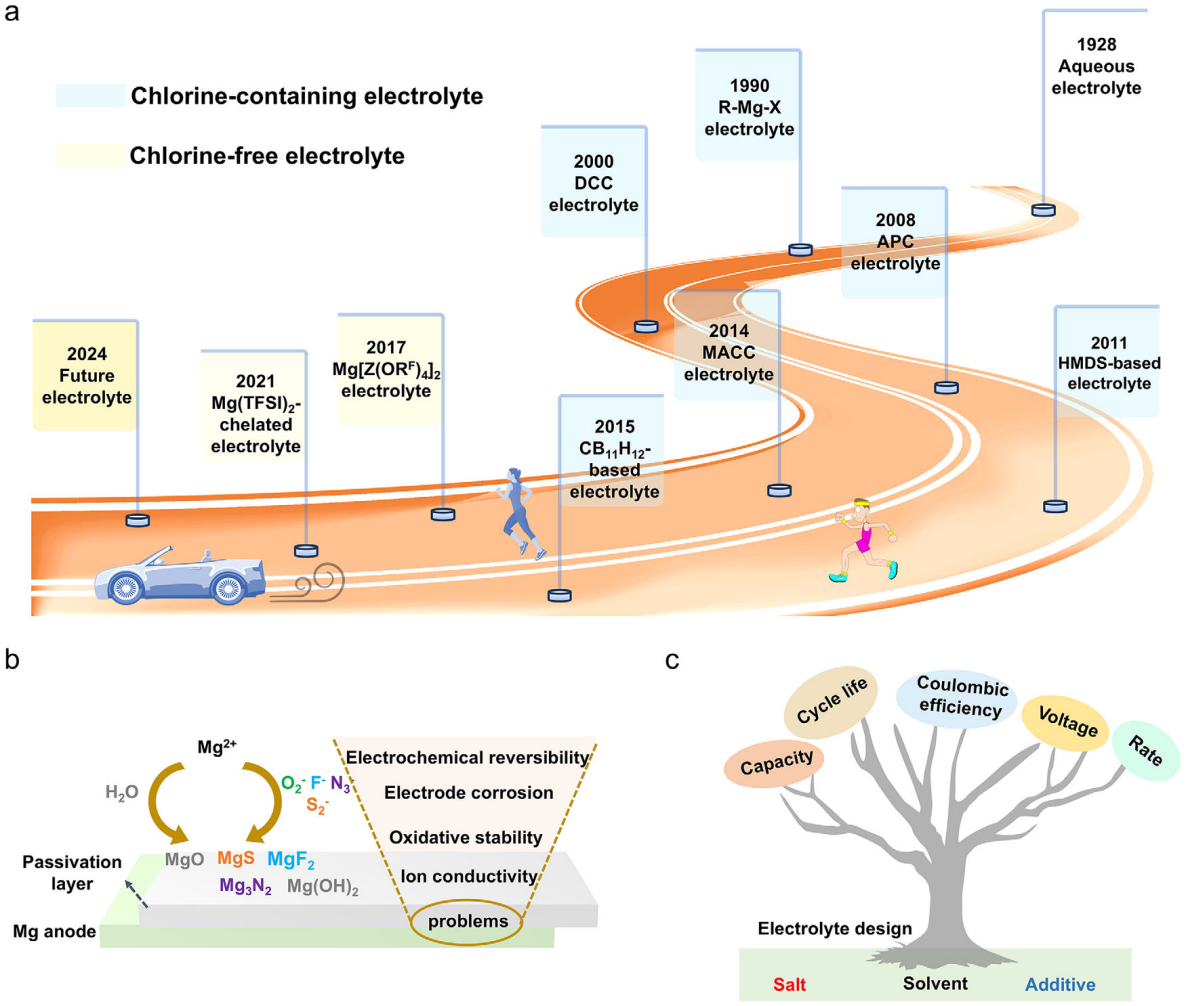
a) A schematic overview of the development of non‐aqueous Mg battery electrolytes. b) Schematic diagram of passivation phenomena. c) Schematic diagram of electrolytes influencing the electrochemical properties of Mg batteries.

Advances in electrolyte formulation are essential to improving performance, as suggested by **Figure** **7**a. As noted above, Mg battery electrolytes can be broadly categorized as chlorine‐containing and chlorine‐free types, each with distinct properties and performance characteristics. The initial chlorine‐containing electrolyte consisted of simple MgCl_2_ used in the molten form for electrolytic deposition of Mg metal. However, MgCl_2_ could not be used directly in non‐aqueous electrolytes as it offers poor solubility in organic solvents (ethers, esters, etc.), resulting in very low electrochemical activity. It was then revealed that Grignard reagents, well known in organic chemistry, can also serve as Mg battery electrolytes, allowing for reversible Mg plating/stripping.^[^ ^174^, ^175^, ^176^ ^]^ However, Grignard reagents are prone to oxidation during charging and extremely sensitive to air/moisture, leading to poor electrolyte stability and low Mg^2+^ conductivities. Later, it was discovered that the electrochemical stability of Grignard reagents could be dramatically enhanced by adding selected Lewis acids (haloaluminates), producing first‐generation dichloro‐complex (DCC) electrolytes.^[^ ^39^, ^177^, ^178^, ^179^ ^]^ DCC electrolytes broaden the accessible electrochemical potential to 2–3 V vs Mg^2+^/Mg (< 1.8 V vs Mg^2+^/Mg for Grignard reagents). However, the oxidation stability was still compromised, primarily due to the susceptibility of AlCl_4‐_ *_n_* R*_n_* ^−^ to undergo β‐H elimination reactions. Second‐generation electrolytes were developed based on PhMgCl, known as all‐phenyl complex (APC) electrolytes. Then, it was found that adding chloride salts (MgCl_2_, AlCl_3_, etc.) to the electrolytes based on traditional Mg salts like magnesium bis(trifluoromethanesulfonimide) [Mg(TFSI)_2_], magnesium bis(hexamethyldisilazide) [Mg(HMDS)_2_], and magnesium trifluoromethanesulfonate [Mg(Otf)_2_] can greatly improve electrolyte performance. Providing favorable ionic conductivity and stability, chlorine‐containing electrolytes still raise significant safety and environmental concerns.^[^ ^180^, ^181^, ^182^ ^]^ Their corrosive nature and potential to release chlorine‐containing toxic by‐products pose challenges for handling, storage, and disposal (recycle), limiting their suitability for large‐scale applications.^[^ ^14^ ^]^

In contrast, chlorine‐free electrolytes offer a safer and more environmentally friendly option,^[^ ^183^ ^]^ where common Mg salts including Mg(BF_4_)_2_, MgSO_4_, Mg(NO_3_)_2_, Mg(TFSI)_2_, Mg(Otf)_2_, and Mg(HMDS)_2_ have been explored. However, achieving reversible Mg plating/stripping in chlorine‐free electrolytes is typically challenging,^[^ ^184^ ^]^ especially in common solvents like acetonitrile and alkyl carbonate, etc. Mg(TFSI)_2_ represents the most widely studied Mg salt for chlorine‐free electrolytes.^[^ ^26^ ^]^ For example, Ha et al. reported one useful Mg(TFSI)_2_/glyme/diglyme electrolyte, showing a high anodic limit (4.0 V vs Mg^2+^/Mg) and permitting ready Mg stripping/deposition. However, TFSI^−^ ions were found to be unstable in ether electrolytes, where they react easily with magnesium metal to form passive surface films.^[^ ^11^ ^]^ Improvements in these systems were made through the addition of dimethylamine (DMA), diethylamine (DEA), and 1‐methoxy‐2‐propylamine (M4).^[^ ^185^ ^]^ These additives can not only preferentially adsorb on Mg surfaces, but also change the Mg^2+^ solvation structure and the reconstruction energy involved in electron transfer. However, these additives usually show nucleophilic behavior and exhibit poor oxidation stability. Other inorganic Mg salts like Mg(FSI)_2_, MgSO_4_, Mg(HMDS)_2_, and Mg(BF_4_)_2_ suffer from similar issues.

The choice between chlorine‐containing and chlorine‐free electrolytes in Mg batteries is primarily governed by the specific electrochemical requirements, including metal–electrolyte compatibility, oxidative stability, ionic conductivity, and the nature of the cathode reaction, rather than solely by the intended application. For example, Mg anodes are highly reactive with easily reduced compounds, including hydrocarbons, alcohols, phenols, and water. This reactivity, combined with electrolyte decomposition, generates electrode passivation layers consisting of compounds like MgO, Mg(OH)_2_, MgS, MgF_2_, and Mg_3_N_2_ (Figure 7b).^[^ ^186^ ^]^ These layers hinder electron and ion transport, thereby damaging the electrode and interfering with reversible Mg plating/stripping.

In addition, achieving high ionic conductivity in Mg battery electrolytes is challenging. The difficult desolvation process at the electrode–electrolyte interface also plays a critical role in limiting the rate capability. The strong coordination between Mg^2+^ and solvent molecules results in a high desolvation energy barrier, which retards interfacial charge transfer and exacerbates polarization under high current densities. Anions and their solvated structures most commonly determine Mg battery electrochemical characteristics greatly impacting operating voltage, capacity, cycle lifeability, Coulombic efficiency, and rate capabilities (Figure 7c). Therefore, it is essential to tailor the anions of Mg salts at the molecular level, select appropriate solvents, and incorporate specific additives.

### 3.1 Salts

**Figure 8:**
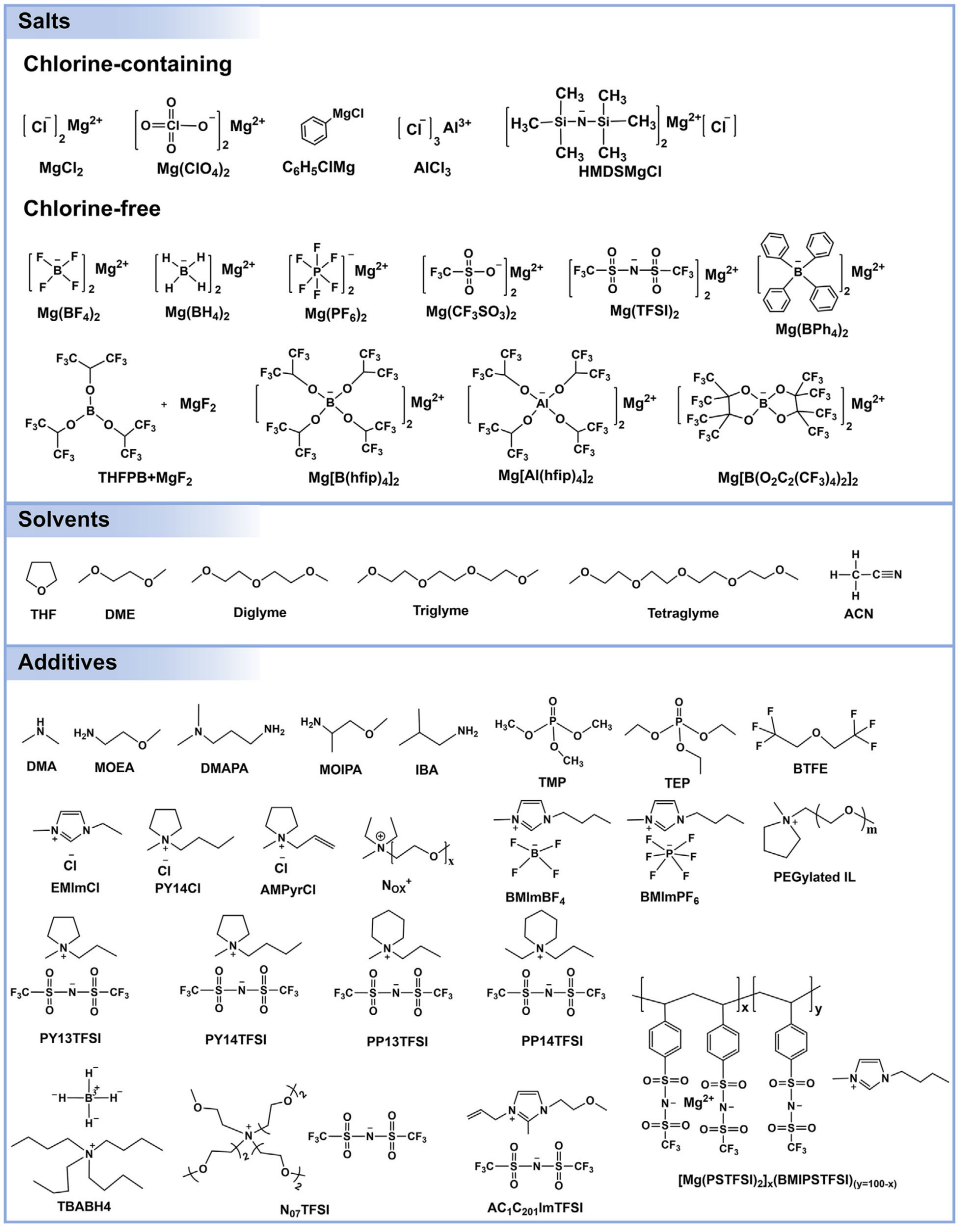
Chemical structures of representative salts, solvents, and additives for Mg battery electrolytes.

The selection of Mg salts is crucial, as it significantly impacts ionic conductivity, electrochemical stability, and compatibility with electrodes. **Figure** **8** displays representative Mg battery chlorine‐containing/free salts. Suitable Mg salts must meet several criteria: high solubility in the chosen solvent, excellent thermal stability to ensure safety and facilitate processing, and a wide redox potential window. Furthermore, the salt should enable reversible Mg plating/stripping and be cost‐effective. Typically used anions include high‐valence elements such as B, Al, P, S, Cl, and As with common functional groups (e.g., –F, –Cl, –OR, –O).^[^ ^19^ ^]^ Unfortunately, anions like PF_6_^−^ and AsF_6_^−^ decompose, forming insulating salts when Mg is reduced at the anode. Moreover, the solubility significantly impacts cycling performance, mandating sufficient concentrations of dissolved salts to maximize ionic conductivity for efficient ionic transport and redox reactions.

Multiple Mg electrolyte salts with diverse anionic structures have been developed to address the challenges of Mg^2+^ transport and Mg metal compatibility in Mg batteries. One of the earliest and most extensively studied systems is the MgCl_2_–AlCl_3_‐based electrolyte (MACC), which forms in ethereal solvents such as THF and supports reversible Mg plating and stripping.^[^ ^187^ ^]^ While it exhibits good ionic conductivity and metal compatibility, its practical application is hindered by a narrow electrochemical stability window (≈2.5 V vs Mg^2+^/Mg), strong corrosiveness, and sensitivity to moisture, limiting its scalability. In contrast, magnesium tetrakis(hexafluoroisopropyloxy)borate (Mg[B(hfip)_4_]_2_) represents a newer class of non‐nucleophilic, weakly coordinating borate‐based electrolytes.^[^ ^188^ ^]^ It offers excellent oxidative stability (>4 V vs Mg^2+^/Mg), efficient Mg cycling, and broad solvent compatibility, although its synthesis involves expensive fluorinated reagents that may impact large‐scale production. Similarly, magnesium fluorinated alkoxy‐aluminate (Mg[Al(hfip)_4_]_2_) features a highly fluorinated, weakly coordinating anionic structure, delivering an even broader electrochemical stability window (>4.3 V) and fast Mg^2+^ transport.^[^ ^189^, ^190^ ^]^ It demonstrates promising electrochemical performance and cathode compatibility, yet its reliance on complex and costly synthesis routes may limit widespread use. Mg(TFSI)_2_ is another widely explored salt known for its high oxidative stability and availability.^[^ ^191^ ^]^ However, it suffers from poor Mg plating/stripping performance when used alone, due to strong Mg^2+^‐solvent interactions and ion pairing, necessitating the use of additives or co‐salts for functional application. Lastly, Mg(HMDS)_2_ is an organometallic precursor that serves primarily as a modular building block for constructing active Mg electrolyte systems.^[^ ^145^ ^]^ Although it is soluble in ether‐based solvents and commercially available, it cannot support reversible Mg plating/stripping on its own and is typically combined with Lewis acids to generate electrochemically viable complexes.

### 3.2 Solvents

In addition, careful consideration of several factors should be taken for choosing electrolyte solvents for Mg batteries: (1) the capability to dissolve Mg salts in a given solvent, (2) the underlying mechanisms involved in efficient Mg plating/stripping, and (3) Mg^2+^ mobility in the solution. These aspects are closely governed by fundamental solvent properties such as viscosity, DN, polarity, dielectric constant,^[^ ^192^, ^193^, ^194^, ^195^ ^]^ and ESW.^[^ ^196^ ^]^

Commonly used electrolyte solvents for non‐aqueous battery electrolytes include ethers, carbonates, amines, and amides. Among these, ethers are the most widely adopted due to their ability to solvate Mg^2+^ effectively through coordination with lone pairs on oxygen atoms, forming stable solvated Mg complexes that support reversible electrochemistry. Examples include tetrahydrofuran (THF), ethylene glycol dimethyl ether (DME or G1), diethylene glycol dimethyl ether (G2), triglyceride dimethyl ether (G3), and tetraethylene glycol dimethyl ether (G4). **Table** **1** records their physicochemical properties, highlighting their intrinsic stability toward oxidation and reduction.^[^ ^197^ ^]^ THF, for instance, is a low‐viscosity ether (≈0.55 mPa s at 25°C) with a moderate DN (≈20 kcal mol^−1^) and good volatility, offering excellent Mg salt solubility and facile ion transport. Its relatively wide electrochemical stability window (0–3 V vs Mg^2+^/Mg) and compatibility with Mg metal make it a standard solvent. However, THF is susceptible to peroxide formation and degradation under extended cycling. G1 and G2 present close DNs and lower volatility than THF, with slightly higher viscosities (≈0.45‐0.92 mPa s). These linear ethers enable the dissolution of various Mg salts, support higher ionic conductivities, and exhibit relatively stable complexation behavior with Mg^2+^. G3 and G4, longer‐chain polyethers, offer improved thermal and oxidative stability and higher dielectric constants, but their increasing viscosity and steric bulk may hinder ion mobilities and reduce rate performance. Therefore, while glymes support a broader stability range and compatibility with high‐voltage cathodes, trade‐offs exist between ion transport efficiency and viscosity.

**Table 1:** Physicochemical properties of the common ether solvents in electrolytes.^[^ ^197^ ^]^

Solvent	Dielectric constant	Viscosity [mPa s, 25°C]	Donor number [kcal mol^−1^]	Boiling point [°C]	Melting point [°C]	Oxidation/reduction potential (V vs Mg^2+^/Mg)
THF	7.5	0.55	20.0	66	−108	8.0/−0.9
DME/G1	7.2	0.45	24.0	85	−69	8.1/−2.5
G2	7.2	0.92	19.5	162	−64	7.5/−2.3
G3	7.5	3.9		216	−45	‐/−2.5
G4	7.5	4.6	16.6	275	−30	7.6/−2.8

Additionally, carbonate solvents such as propylene carbonate (PC) and dimethyl carbonate (DMC) offer high dielectric constants, which can enhance the dissociation of Mg salts and increase ionic conductivity. However, these solvents typically exhibit poor compatibility with Mg metal due to the formation of passivation layers composed of decomposition products (e.g., MgCO_3_, Mg(OH)_2_), which block Mg^2+^ transport and hinder electrochemical reversibility. Their relatively low DNs and limited coordination ability further reduce their ability to stabilize Mg^2+^ species effectively, making them less favorable for Mg anodes despite their success in LIB systems. Other less commonly explored solvent classes include amides and amines, such as N,N‐dimethylformamide (DMF) or N‐methylpyrrolidone (NMP), which possess high DNs and strong coordinating ability. These can in theory stabilize Mg^2+^ effectively, but often suffer from high viscosity, narrow electrochemical stability, and potential reactivity with Mg metal.

### 3.3 Additives

Electrolyte additive engineering has emerged as a powerful strategy to address multiple intrinsic challenges associated with Mg anodes, particularly the sluggish kinetics of Mg^2+^ transport, the high tendency of salt‐solvent complexation, and poor interfacial compatibility with Mg metal. Moreover, additives also affect formation of efficient SEIs on anodes and CEIs on cathodes, closely reflected by HOMO and LUMO energy levels. The selection of additives for Mg electrolytes is guided by several criteria. First, additives must not react unfavorably with other electrolyte components, the Mg anode, or electrode materials. Electrolyte additives are expected to be stable in the bulk electrolyte during operation, while undergoing controllable interfacial decomposition at appropriate potentials to generate a robust and ion‐conductive SEI on the Mg anode. Second, the additive with functional effectiveness must perform its intended role, whether it is to enhance Mg deposition, stabilize the interface, or scavenge impurities. Lastly, practical considerations like cost and scalability are important for real‐world applications.

Therefore, just like the critical design of salts and solvents, the rational incorporation of additives is key to fine‐tuning electrolyte behavior and ensuring high performance. These additives can be systematically classified based on their function into four primary categories: SEI‐inducing additives, chelating additives, solvation‐tuning additives, and ionic liquid additives. The formation of a stable, ionically conducive, and electronically insulating SEI on Mg anodes is a fundamental requirement for reversible cycling. However, unlike lithium, magnesium does not naturally form a self‐passivating SEI in most conventional solvents. Additives that decompose before the solvent and deposit beneficial interphase layers are often introduced to solve this issue. Moreover, chelating additives function by coordinating with Mg^2+^ in solution, disrupting strongly bound ion pairs or aggregates that otherwise limit transport. Chelation can significantly lower the effective charge density of Mg^2+^, facilitating its movement across the electrolyte. Besides, solvation–tuning additives aim to regulate the solvation structure of Mg^2+^ in the electrolyte by either replacing primary solvent molecules or altering the solvent coordination number. In addition, ionic liquid additives offer several desirable properties such as low volatility, high thermal stability, wide electrochemical windows, and intrinsic ionic conductivity. When used as co‐solvents or additives, they can enhance Mg salt solubility and electrode compatibility.

### 3.4 Electrolyte Design Guidelines

The performance of Mg battery electrolytes is strongly influenced by the molecular properties of salts, solvents, and additives. Rational design requires understanding how these properties collectively regulate Mg^2+^ solvation, desolvation, and interfacial kinetics. The choice of Mg salts determines both ionic conductivity and electrode compatibility. Key factors include anion charge delocalization, size, and coordination behavior. Anions with delocalized charge, such as TFSI^−^ or fluorinated borates, reduce coulombic interaction with Mg^2+^, lowering desolvation barriers and facilitating smooth Mg plating/stripping. Bulky or weakly coordinating anions also enhance the electrochemical stability window by mitigating nucleophilic attack on the solvent or electrode. Conversely, strongly coordinating halide anions improve Mg^2+^ solubility but may promote corrosion or side reactions at the electrode. Optimizing the balance between coordination strength and chemical stability is thus essential.

Solvent selection critically affects salt solubility, ionic mobility, and Mg^2+^ desolvation energy. Solvents with high DN coordinate strongly with Mg^2+^,^[^ ^192^, ^193^, ^194^, ^195^ ^]^ stabilizing solvated complexes but potentially increasing the interfacial desolvation barrier. Moderate DN values ensure sufficient salt solubility while maintaining fast electrode kinetics. Dielectric constant influences ion dissociation; high dielectric constant enhances conductivity but may destabilize reactive anions or accelerate side reactions.^[^ ^192^, ^193^, ^194^, ^195^ ^]^ Solvent polarity, viscosity, and Lewis basicity must be carefully tuned to achieve high ionic conductivity, low overpotential, and stable electrochemical operation. Co‐solvents or mixed solvent systems can further fine‐tune solvation structures and interfacial behavior.

Electrolyte additives serve to enhance interfacial stability, suppress parasitic reactions, and improve Mg deposition morphology. Halide or Lewis‐acidic additives can promote in situ SEI formation on the Mg surface, enhancing reversibility. Fluorinated or boron‐containing additives stabilize both cations and anions, preventing decomposition and extending anodic stability. Redox mediators or chelating agents can modulate local Mg^2+^ concentration and deposition uniformity. Optimal additive design involves selecting species that act synergistically with the salt and solvent environment, ensuring long‐term chemical stability while maintaining high ionic conductivity and low plating/stripping overpotentials.

Overall, the performance of non‐aqueous Mg battery electrolytes is critically dependent on the synergistic design of salts, solvents, and additives. While chlorine‐containing electrolytes exhibit favorable Mg compatibility, they often raise concerns regarding stability and safety. In contrast, chlorine‐free alternatives offer improved oxidative stability but frequently suffer from sluggish Mg^2+^ transport kinetics. Thereby, rational solvent selection and strategic additive engineering are essential to optimize solvation structures and enhance interfacial stability. Such efforts are key to advancing practical, high‐performance magnesium electrolytes.

## 4 Advancement in Chlorine‐Containing Electrolytes

Research on Mg battery electrolytes can be traced back to the 1920s,^[^ ^176^ ^]^ focusing on Mg Grignard reagents. It was discovered that Mg is not able to deposit smoothly from solutions containing simple Mg salts in acetonitrile, propylene carbonate, thionyl chloride, or N,N‐dimethylformamide.^[^ ^184^, ^198^, ^199^ ^]^ It is mainly because of the formation of dense passivating layers that contain MgO, Mg(OH)_2_, MgCO_3_, and Mg(ROCO_2_)_2_ with very poor Mg^2+^ conductivity.^[^ ^200^, ^201^ ^]^ Furthermore, Mg plating/stripping efficiencies are sensitively affected by even ppm levels of water, which significantly raises the overpotential for Mg deposition. Mg dissolution can take place only via breaking down the passivating surface films when the electrodes are anodically polarized. Unfortunately, cathodic polarization would reform the passivation layers and even thicken them, causing difficulties in achieving efficient Mg deposition in reactive solvents, such as alkyl carbonates, acetonitrile, and esters.

To address these challenges, chlorine‐containing electrolytes have shown great promise in enabling reversible Mg deposition and stripping. **Table** **2** displays the properties of reported chlorine‐containing electrolytes. The presence of Cl^−^ ions facilitates the formation of soluble Mg–Cl complexes, which significantly lowers the desolvation barrier for Mg^2+^ at the electrode interface. These complexes not only reduce the effective charge density of Mg^2+^, improving its mobility, but also help prevent the formation of electronically insulating passivation layers on the Mg surface. Moreover, Cl^−^ anions can stabilize the electrode/electrolyte interface and enhance interfacial kinetics, allowing for smoother and more reversible Mg plating/stripping processes. As a result, Cl‐based electrolytes enable more efficient Mg cycling, even under ambient conditions, making them a critical component for practical Mg battery systems.

**Table 2:** The properties of Mg battery chlorine‐containing electrolytes.

Classification	Electrolyte	IC [mS cm^−1^]	ESW (V vs Mg^2+^/Mg) (CE)			Refs.
			Pt	Cu	Au	
Grignard reagents	1 M EtMgBr/THF	0.265	≈1.5			[202]
	1 M EtMgBr/THF				≈1.1	[203]
	1 M BuMgCl/THF				≈1.5	[204]
	0.25 M BuMgCl/THF					[205]
	2 M PhMgCl/THF	0.2				[206]
	1 M PhMgBr/THF	0.252	≈1.8			[202]
	1 M FPhMgBr/THF	0.398	≈2.4			[202]
Magnesium organo–haloaluminate based electrolytes	0.25 M Mg(AlCl)_2_BuEt/THF				≈2.5 (100%)	[204]
	0.25 M Mg(AlCl_2_BuEt)_2_/THF		> 2.5 (100%)			[39]
	0.25 M Mg(AlCl_2_BuEt)_2_/THF				≈2.1 (100%)	[35]
	0.25 M Mg(AlCl_3_Bu)_2_/THF				2.5 (100%)	[35]
	0.25 M AlCl_2_Et+Bu_2_R/THF				>2.1 (100%)	[207]
	0.25 M Bu_2_Mg+2AlCl_2_Et/THF	1.6			≈2.3 (97%)	[208]
	0.25 M Mg(AlCl_2_EtBu)_2_/THF	1.8	≈2.64			[205]
	0.4 M (PhMgCl)_2_–AlCl_3_/THF		>3 (100%)			[205]
Inorganic electrolytes	0.25 M Mg_2_AlCl_7_/DME	2	≈3.1 (99%)			[187]
	0.04 M (µ‐Cl)_3_Mg_2_(THF)_6_AlCl_4_/THF	0.26	≈3.4 (100%)			[209]
	0.67 M (µ‐Cl)_3_Mg_2_(THF)_6_AlEtCl_3_/THF	6.99	2.9 (100%)			[209]
	0.43 M (µ‐Cl)_3_Mg_2_(THF)_6_AlPh_3_Cl/THF	2.96	3.1 (100%)			[209]
	(µ‐Cl)_3_Mg_2_(THF)_6_AlPh_3_Cl/THF	0.96	≈3.0 (100%)			[210]
	0.4 M Mg_2_(µ‐Cl)_2_(DME)_4_ (AlEtCl_3_)_2_/DME	>6	≈3.5 (99%)			[211]
Mg(TFSI)_2_‐based electrolytes	0.4 M Mg(TFSI)_2_–MgCl_2_/DME		≈3.5 (80%)			[212]
	0.25 M (Mg(TFSI)_2_)_2_–MgCl_2_/THF		>3 (42%)			[191]
	0.5 M Mg(TFSI)_2_–MgCl_2_/THF		>3 (42%)			[191]
	0.25 M (Mg(TFSI)_2_)_2_–MgCl_2_/G2		>3.5 (93%)			[191]
	0.5 M Mg(TFSI)_2_–MgCl_2_/G2		>3.5 (75%)			[191]
	0.03 M (MgCl_2_)_2_–AlCl_3_–Mg(TFSI)_2_/THF		97%	>2.3		[213]
	0.03 M (MgCl_2_)_2_–AlCl_3_–Mg(TFSI)_2_/THF	0.28	3.1(>96%)			[214]
	0.25 M (MgCl_2_)_2_–AlCl_3_–Mg(TFSI)_2_/DME	6.82	3.2(>96%)			[214]
	0.2 M Mg(TFSI)_2_–(MgCl_2_)_2_/DME+THF (1:3 vol%)		>98%			[171]
	0.3 M Mg(TFSI)_2_–(MgCl_2_)_2_/DME+TFEB (95:5 vol%)		>3.0 (97.87%)			[54]
Mg(HMDS)_2_‐based electrolytes	0.5 M Mg(HMDS)_2_–2MgCl_2_/THF		≈2.75(95%)			[215]
	0.5 M Mg(HMDS)_2_–4MgCl_2_/THF	0.32	≈2.8 (99%)			[215]
	0.5 M 2 Mg(HMDS)_2_–MgCl_2_/THF		≈2.3(85%)			[215]
	1.44 M HMDSMgCl–AlCl_3_/THF		≈3.2 (100%)			[145]
	0.25 M (HMDS)_2_Mg–(AlCl_3_)_2_/THF	1.72	3.3 (98%)			[216]
	0.25 M (HMDS)_2_Mg–(AlCl_3_)_2_/G2	1.70	3.9 (99%)			[216]
	0.25 M (HMDS)_2_Mg–(AlCl_3_)_2_/2‐Me‐THF	1.28	2.8 (98%)			[216]
	0.25 M (HMDS)_2_Mg–(AlCl_3_)_2_/G4	0.70	3.7 (97%)			[216]
	0.25 M (HMDS)_2_Mg–(AlCl_3_)_2_/THF+G4 (1:1 vol%)	1.34	3.6(98%)			[216]
Boron‐containing salt‐based electrolytes	0.25 M Bu_2_Mg–BCl_3_/THF				1.30‐1.77 (68‐93%)	[207]
	0.5 M Mes_3_B–PhMgCl/THF	1.3				[206]
	0.5 M Mes_3_B–(PhMgCl)_2_/THF	2	≈3.5 (98%)			[206]
	(1‐(1,7‐C_2_B_10_H_11_)_2_MgCl)(Mg_2_Cl_3_)/THF	0.6	≈3.2 (>98%)			[217]

### 4.1 Grignard Reagent‐Based Electrolytes

The presence of acidic species can remove passivating layers and enable Mg dissolution at relatively low overpotentials.^[^ ^184^ ^]^ The discovery of Grignard reagents laid an important foundation for the study of electrolytes.^[^ ^174^, ^175^ ^]^ In particular, the THF solvent used in Grignard reagents belongs to a class of aprotic ether solvents, which are not reactive with Mg. Meanwhile, Grignard reagents of RMgX (R = alkyl, X = Cl, Br) can avoid the formation of passivating layers on Mg electrodes, thus facilitating reversible Mg plating/stripping. Grignard reagents are typically denoted as RMgX (X = F, Cl, and Br, R = methyl (Me), butyl (Bu), and benzyl (Ph), etc). Grignard reagents are highly nucleophilic and readily undergo reduction. As early as 1912, it was recognized that Mg could be effectively deposited using Grignard/ether complexes, predating their application in rechargeable Mg batteries.^[^ ^218^, ^219^, ^220^ ^]^ This is considered the earliest known electrolyte capable of enabling reversible Mg plating and stripping. Nelson et al. were the first to experimentally investigate the ionic conductivity of Grignard reagents.^[^ ^174^ ^]^ Later, Conno et al. found that Grignard reagent (EtMgBr) produced readily available white and metallic Mg deposits,^[^ ^218^ ^]^ albeit not entirely pure. The addition of LiBH_4_ greatly improved both electrolyte conductivities and the purity of the deposited Mg (90%).^[^ ^220^ ^]^ These results indicated that Grignard reagents might play a crucial role in Mg deposition and dissolution. Subsequently, Gaddum and French et al. proposed reaction sequences (Equations (4), (5), (6), (7), (8), (9)) to describe these processes.^[^ ^175^ ^]^

(4) $$C_{6}H_{5}C{H_{2}}^{-}+Mg^{2+}+Cl^{-}\to C_{6}H_{5}CH_{2}\mathrm{MgCl}$$

(5) $$C_{6}H_{5}CH_{2}\mathrm{MgCl}↔C_{6}H_{5}C{H_{2}}^{-}+Mg^{2+}+Cl^{-}$$

(6) $${\mathrm{Mg}}^{2+}+2e^{-}\to \mathrm{Mg}$$

(7) $$Mg^{2+}+2Cl^{-}\to \mathrm{MgC}l_{2}$$

(8) $$C_{6}H_{5}C{H_{2}}^{-}\to C_{6}H_{5}C{H_{2}}^{+}+2e^{-}$$

(9) $$C_{6}H_{5}C{H_{2}}^{-}+C_{6}H_{5}C{H_{2}}^{+}\to C_{6}H_{5}CH_{2}-C_{6}H_{5}CH_{2}$$

In 1999, Lu et al. noted that non‐protic ether solvents could eliminate the formation of problematic passivation layers during cycling.^[^ ^184^ ^]^ Additionally, Mg deposition from these solutions does not follow a simple double‐electron transfer mechanism. Instead, it was proposed that electron transfer occurred during the adsorption and desorption of species formed via the reversible dissociation of Grignard reagents salts, such as MgX_2_ (X = Cl, Br, and I) and MgR_2_ (R = Me, Bu, and Ph). Equations (10) and (11) describe the likely equilibrium reactions.^[^ ^221^ ^]^ Equations (12)–(15) illustrate the reactions involved in Mg deposition, while Equations (16) and (17) present further reactions in the solution. Finally, Equations (18) and (19) represent the Mg dissolution processes, with Equation (20) summarizing the overall reaction.

(10) $$2\mathrm{RMgX}↔\mathrm{Mg}R_{2}+\mathrm{Mg}X_{2}$$

(11) $$2\mathrm{RMgX}↔\mathrm{RM}g^{+}+\mathrm{RMg}{X_{2}}^{-}$$

(12) $$2\mathrm{RM}g^{+}+2e^{-}↔2\mathrm{RM}g_{\left(\mathrm{ad}\right)}$$

(13) $$2\mathrm{RM}g_{\left(\mathrm{ad}\right)}↔\mathrm{Mg}+\mathrm{Mg}R_{2(\mathrm{sol})}$$

(14) $$2\mathrm{Mg}R_{2}+2e^{-}↔2\mathrm{RM}g_{\left(\mathrm{ad}\right)}+2R^{-}$$

(15) $$2\mathrm{RM}g_{\left(\mathrm{ad}\right)}↔\mathrm{Mg}+\mathrm{Mg}R_{2(\mathrm{sol})}$$

(16) $$R^{-}+\mathrm{RM}g^{+}↔\mathrm{Mg}R_{2(\mathrm{sol})}$$

(17)

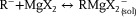



(18)

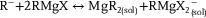

(19) $$\mathrm{Mg}+\mathrm{Mg}R_{2}↔2e^{-}+\mathrm{RM}g^{+}$$

(20) $$\mathrm{Mg}+2\mathrm{RMg}{X_{2}}^{-}↔2e^{-}+\mathrm{Mg}R_{2}+\mathrm{Mg}X_{2}$$

**Figure 9:**
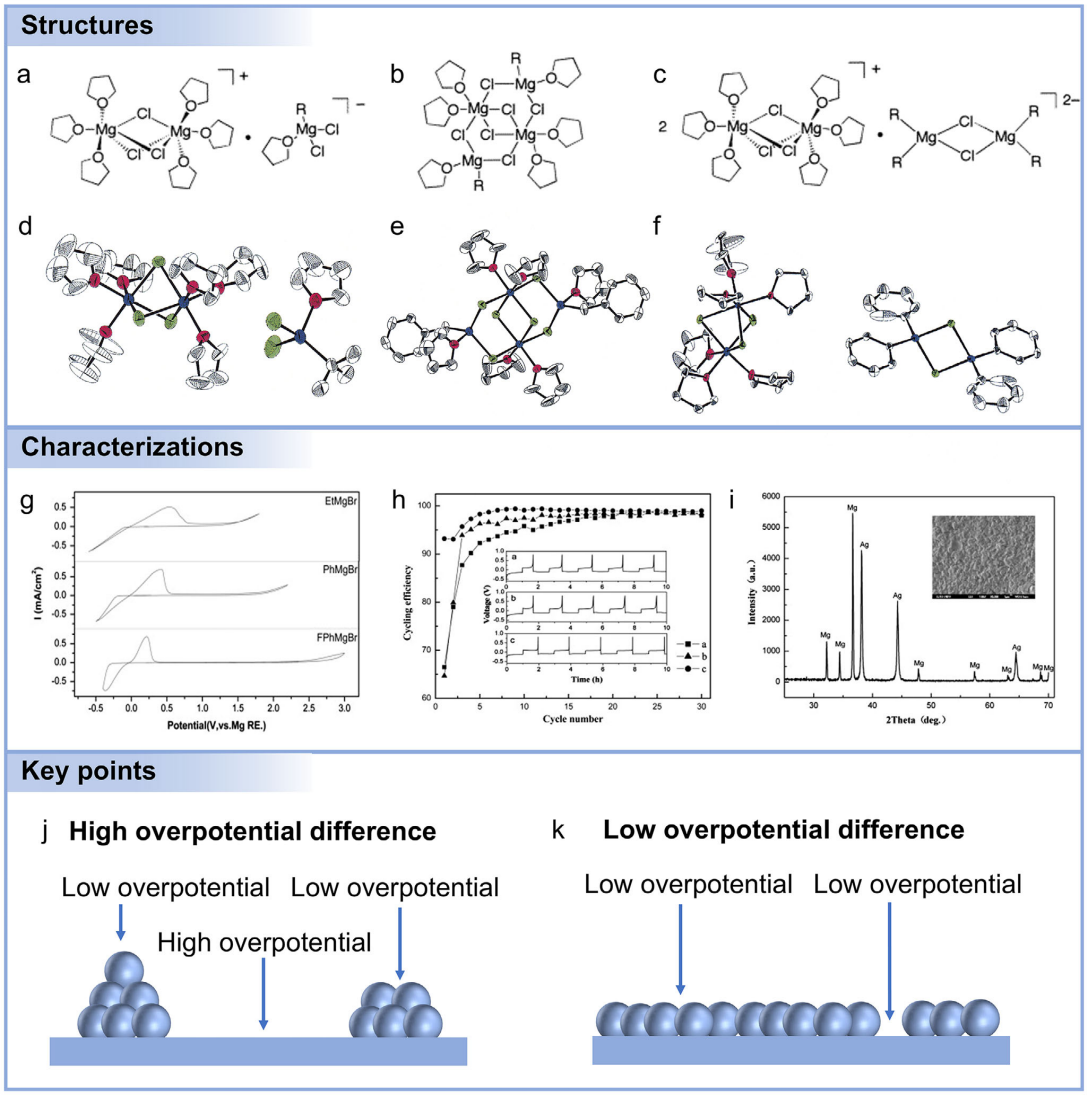
Crystal structures of a) [Mg_2_(µ‐Cl)_3_(THF)_6_][RMgCl_2_(THF)], b) R_2_Mg_4_Cl_6_(THF)_6_, and c) [Mg_2_(µ‐Cl)_3_(THF)_6_]_2_(R_4_Mg_2_Cl_2_). Oak Ridge thermal ellipsoid plot (ORTEP) of d) [Mg_2_(µ‐Cl)_3_(THF)_6_][t‐BuMgCl_2_(THF)], e) Ph_2_Mg_4_Cl_6_(THF)_6,_ and f) [Mg_2_(µ‐Cl)_3_(THF)_6_]_2_(Ph_4_Mg_2_Cl_2_). Reproduced with permission.^[^ ^222^ ^]^ Copyright 2001, American Chemical Society. g) Typical steady‐state voltammograms of Pt electrodes in 1 M RMgBr/THF solutions, scanning rate: 50 mV s^−1^. h) The cycling efficiencies and the first five chronopotentiograms of Mg deposition and dissolution on Cu in THF solutions containing 1 M EtMgBr [a], PhMgBr [b], and F‐PhMgBr [c]. i) XRD pattern of the electrodeposited Mg on Ag substrate from 1 M F‐PhMgBr/THF solution and its SEM image. The charge amount is 3.6 C cm^−2^. Reproduced with permission.^[^ ^202^ ^]^ Copyright 2010, Elsevier. Schematic diagram of Mg deposition on Pt from THF solution with j) high overpotential difference and (k) low overpotential difference on the Pt surface and Mg deposits.

Sakamoto et al. investigated the structural components of Grignard reagents using X‐ray crystallography and cold‐spray ionization mass spectrometry (CSI‐MS).^[^ ^222^ ^]^ As depicted in **Figure** **9**a–f, three distinct species, [Mg_2_(µ‐Cl_3_)(THF)_6_][RMgCl_2_(THF)], R_2_Mg_4_Cl_6_(THF)_6_, and [Mg_2_(µ‐Cl_3_)(THF)_6_]_2_(R_4_Mg_2_Cl_2_), were identified in RMgCl (R = Me, *t*‐Bu, Ph) solutions. In MeMgCl/THF solution, MeMg_2_(µ‐Cl_3_)(THF)_4‐6_ species were identified to be the predominant components. Then, Brennern et al. prepared electrolyte solutions through the reaction of MeMgCl with triethylboron,^[^ ^223^ ^]^ which facilitated the deposition of white and ductile Mg with Coulombic efficiencies approaching 100%. Genders et al. demonstrated that the Grignard reagent (EtMgBr/THF) enabled efficient electroplating of Mg onto Cu substrates and anodic redissolution with high Coulombic efficiency when using microelectrodes (Cu discs with diameters of 40 and 80 µm).^[^ ^219^ ^]^ Liebenow et al. reported that Grignard reagents offered low Mg^2+^ conductivities (≈0.4 mS cm^−1^) and limited oxidative stability (≈1.5 V vs Mg^2+^/Mg),^[^ ^224^, ^225^ ^]^ both of which were influenced by the nature of the R and X groups (R = alkyl; X = Br, Cl). Additionally, dendritic deposition was found to be highly substrate‐dependent. Guo et al. investigated the electrochemical behavior of Mg plating/stripping using three Grignard reagents, RMgBr (R = Et, Ph, 4‐fluorophenyl (4‐F‐Ph)), as shown in Figure 9g–i.^[^ ^202^ ^]^ Their study revealed that the use of the more stable 4‐fluorophenyl group significantly improved the electrochemical reversibility of Mg plating/stripping and enhanced the oxidative stability of the electrolyte.

According to Shunsuke Yagi and colleagues,^[^ ^226^ ^]^ trace levels of water in solutions could heavily decrease the reversibility of Mg plating/stripping, increase the reaction overpotential, cause Mg deposits with varying morphologies, and ultimately lead to the formation of an uneven passivation layer. As shown in Figure 9j–k, surface inhomogeneities result from substantial overpotential differences, causing Mg to deposit predominantly on the surfaces of Mg particles. In contrast, with minimals, Mg deposits uniformly on both Pt and Mg surfaces.

Although Mg has been successfully electro‐deposited with good reduction stability using Grignard reagents, difficulties in desolvation at the electrode/electrolyte interface arise due to the high charge of Mg^2+^. Moreover, Grignard reagents are easily oxidized during charging, resulting in poor electrolyte stability. Consequently, Grignard reagents are generally deemed unsuitable for use with high‐voltage cathode materials.

### 4.2 Magnesium Organo–Haloaluminate Salt‐Based Electrolytes

While Grignard‐based electrolytes laid the foundation for early Mg battery development, they were limited by narrow electrochemical windows and compatibility issues with high‐voltage cathodes. These challenges prompted the development of magnesium organo‐haloaluminate electrolytes, which combined enhanced ionic conductivity, broader electrochemical stability, and improved metal deposition characteristics.^[^ ^211^ ^]^ This evolution reflects a strategic shift from simple organomagnesium reagents toward more complex, tunable electrolyte systems capable of meeting the demands of next‐generation Mg batteries. Aurbach et al. developed a series of magnesium organo‐haloaluminate‐based electrolyte salts with a formula of Mg(AlX_4‐_ *_n_* R′_*n*′_R″_*n*″_)_2_,^[^ ^39^ ^]^ where X is F, Cl, or Br, and R′_n_ and R″ were aryl or alkyl groups with 0 < *n* < 4 (*n*′ + *n*″ = *n*). In comparison to Grignard reagents, these electrolyte salts exhibited superior electrochemical performance, including high Coulombic efficiency (100%), a broader electrochemical window (2‐3 V vs Mg^2+^/Mg), and a lower Mg deposition overpotential. Additionally, they were relatively cost‐effective and readily available at 99% purities.^[^ ^177^ ^]^ And, two categories can be defined: (1) first‐generation DCC electrolytes and (2) second‐generation APC electrolytes.

#### 4.2.1 Dichloro‐Complex Electrolytes

The DCC electrolyte is formulated via the reaction of AX_3‐_ *_n_* R*_n_* Lewis acids with MgR_2_ Lewis bases, resulting in a primary component of Mg(AX_4‐_ *_n_* R*_n_*)_2_, where X represents F, Cl, or Br, and R denotes an aryl or alkyl group. These electrolytes exhibit highly complex compositions, often existing as mixtures.^[^ ^35^ ^]^ For example, the Mg(AlCl_3‐_ *_n_* R*_n_*_+1_)_2_ complex solutions contain various Mg*_x_* Cl*_y_* R*_z_* (*n*THF)^(2^ *^x^* ^‐^ *^y^* ^)+^ species,^[^ ^35^ ^]^ as shown in Equations (21) and (22).

(21) $$\mathrm{Mg}{\left(\mathrm{AlC}l_{3-n}{R_{n}}_{+1}\right)}_{2}↔\mathrm{Mg}R_{2}+\mathrm{AlC}l_{3-n}R_{n}$$

(22) $$\mathrm{Mg}{\left(\mathrm{AlC}l_{3-n}{R_{n}}_{+1}\right)}_{2}↔\left(\mathrm{AlC}l_{3-n}{R_{n}}_{+1}\right)Mg^{+}+{\left(\mathrm{AlC}l_{3-n}{R_{n}}_{+1}\right)}^{-}$$

**Figure 10:**
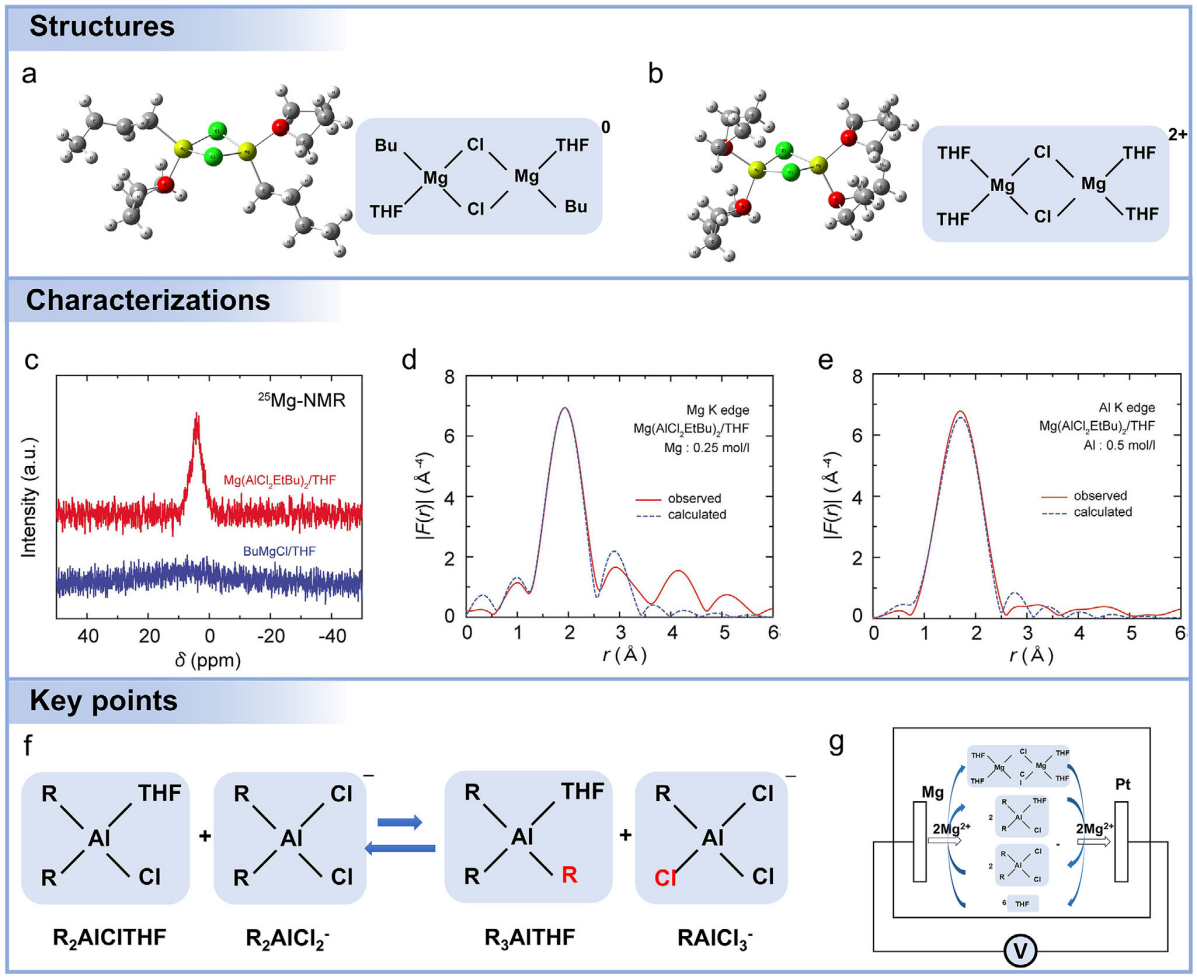
Tetrahedral dimmer structure of neutral Mg complex in a) 0.25 mol L^−1^ BuMgCl/THF: Mg_2_Cl_2_Bu_2_(THF)_2_, and b) tetrahedral dimmer structure of dicationic Mg complex in 0.25 mol L^−1^ Mg(AlCl_2_EtBu)_2_/THF: (Mg_2_Cl_2_THF_4_)^2+^. The atomic structures were visualized using Gaussian View software. c) NMR spectra of 0.25 mol L^−1^ BuMgCl/THF and 0.25 mol L^−1^ Mg(AlCl_2_EtBu)_2_/THF. d) Mg K‐edge EXAFS spectra for 0.25 mol L^−1^ Mg(AlCl_2_EtBu)_2_/THF. Structure used for the calculation was a tetrahedral dimmer [Mg_2_Cl_2_(THF)_4_]^2+^. e) FT of Al K‐edge EXAFS for 0.25 mol L^−1^ Mg(AlCl_2_EtBu)_2_/THF. Mixture of tetrahedral monomers. Reproduced with permission.^[^ ^205^ ^]^ Copyright 2008, IOP Publishing. f) Tetrahedral monomer structures of Al complexes in 0.25 mol L^−1^ Mg(AlCl_2_EtBu)_2_/THF, and their equilibrium, where R = Et and Bu. g) Schematic drawing of the reaction mechanism of Mg deposition on the Pt electrode in 0.25 mol L^−1^ Mg(AlCl_2_EtBu)_2_/THF. In the case of dissolution, the opposite reaction proceeds.

Raman spectra reveal the species present in ethereal electrolyte solutions formed by the reaction of R*_x_* MgCl_2‐_ *_x_* with R’*_y_* AlCl_3‐_ *_y_* (*x* = 0–2, *y* = 0–3),^[^ ^40^, ^177^ ^]^ where the composition, including MgCl_2_, Mg_2_Cl_3_^+^, AlCl_3‐_ *_n_* R*_n_*, and AlCl_4‐_ *_n_* R*_n_* ^−^ (*n* = 1–3), is primarily governed by the ratio of Lewis base to Lewis acid. Utilizing NMR and XAS, Nakayama et al. examined the precise equilibrium configurations in the Mg(AlCl_2_BuEt)_2_/THF electrolyte.^[^ ^205^ ^]^ Consistent with previous studies, tetrahedral Mg dimers, Mg_2_Cl_2_Bu_2_(THF)_2_, were identified in BuMgCl/THF, where two Cl atoms bridge two Mg atoms (**Figure** **10**a).^[^ ^205^, ^227^ ^]^ As shown in Figure 10b, [Mg_2_Cl_2_(THF)_4_]^2+^ is formed alongside other species in 0.25 mol L^−1^ Mg(AlCl_2_EtBu)_2_/THF solution. Moreover, Figure 10c presents the ^25^Mg NMR spectra of 0.25 mol L^−1^ Mg electrolytes, including Mg(AlCl_2_EtBu)_2_/THF and BuMgCl/THF solutions. The absence of a ^25^Mg NMR peak for BuMgCl/THF is ascribed to the lower symmetry of Mg_2_Cl_2_Bu_2_(THF)_2_ compared to [Mg_2_Cl_2_(THF)_4_]^2+^ in the Mg(AlCl_2_BuEt)_2_/THF electrolyte. Mg K‐edge EXAFS spectra for 0.25 mol L^−1^ Mg(AlCl_2_BuEt)_2_/THF and 0.25 mol L^−1^ Mg(AlCl_2_EtBu)_2_/THF are displayed in Figure 10d,e. The Al species are believed to primarily exist as two tetrahedral monomers in a 1:1 ratio of R_2_AlCl(THF):(R_2_AlCl_2_)^−^. Consequently, the electrolyte contains [Mg_2_Cl_2_(THF)_4_]^2+^, R_3_Al(THF), and R_2_AlCl(THF), along side with anionic species such as (R_2_AlCl_2_)^−^ and (RAlCl_3_)^−^ (Figure 10f). In particular, Figure 10g depicts Mg deposition on a Pt electrode in this electrolyte. For the reaction to proceed continuously and reversibly, neutral compounds play a crucial stabilizing role.

Then, AlCl_3_ was introduced into EtMgCl/THF solutions,^[^ ^211^ ^]^ enabling the deposition of regular hexagonal Mg crystallites. Building on this approach, Aurbach and coworkers synthesized Mg(AlCl_2_BuEt)_2_/THF electrolytes.^[^ ^39^ ^]^ Studies demonstrated that this electrolyte exhibited superior oxidation stability compared to Grignard reagents while also achieving reversible Mg plating/stripping.^[^ ^178^ ^]^ One possible explanation for the improved oxidation stability is the electron‐withdrawing effect of halogen ligands, which reduces electron density around the organic aluminum salt core. Indeed, the electrochemical stability window of the 0.25 mol L^−1^ Mg(AlCl*_x_* BuR_3‐_ *_x_*)_2_/THF solution showed a monotonic rise in anodic stability as *x* increased from 0 to 3. Aurbach et al. further investigated electrolytes derived from reaction between MgBu_2_ with a range of Lewis acids (TaF_3_, SbCl_3_, SbCl_5_, AsPh_3_, FeCl_3_, PPh_3_, PEt_2_Cl, BPhCl_2_, BPh_2_Cl, BEt_3_, BF_3_, and BBr_3_) in a variety of ether solvents, such as THF, 2Me‐THF, 1,3‐dioxolane (DO), G1, G2, G3, and diethylether (DEE).^[^ ^207^ ^]^ However, none of these systems supported reversible Mg plating/stripping. In contrast, an electrolyte based on the interaction between MgBu_2_ and AlCl_2_Et at a 1:2 acid‐base molar ratio demonstrated a high Coulombic efficiency (95%) and good oxidation stability (2.1 V vs Mg^2+^/Mg).

Al‐containing anions (AlCl_4‐_ *_n_* R*_n_*)^−^ and Mg^2+^ dimers or oligomers (Mg_2_R_3‐_ *_n_* Cl*_n_*)^+^ form as a result of metal transfer between the Lewis acid and Lewis base. [Mg_2_(µ‐Cl)_3_(THF)_6_]^+^ cations and (EtAlCl_3_)^−^ anions precipitate from this solution as single crystals. However, these species were found to be electrochemically inactive, as they did not support reversible Mg deposition or dissolution. In contrast, the residual solution remaining after crystal precipitation may still facilitate Mg plating and stripping. The presence of Lewis acids introduces electron‐withdrawing interactions, as oxidation of the R–Mg bond limits the anodic stability of these solutions. As the Lewis acid concentration increases, oxidation of the R–Mg bond becomes more difficult. This suggests that the acid stabilizes complex salts against electrochemical oxidation by increasing their electron‐withdrawing effect.

The effect of the R‐group on electrochemical performance was also investigated,^[^ ^179^ ^]^ and the following oxidation stability sequence for electrolytes with different R‐groups was reported: “ethyl‐butyl complex electrolyte” (2.45 V vs Mg^2+^/Mg) < “all‐ethyl complex electrolyte” (2.5 V vs Mg^2+^/Mg) < “all‐methyl complex electrolyte” (2.6 V vs Mg^2+^/Mg). These results indicate that as the R‐group chain length decreases (Bu > Et > Me), the stability of the Al–R bond increases, enhancing oxidative stability. Despite these findings, the DCC electrolyte exhibited very high resistance when using Mg electrodes. This was initially attributed to the formation of a solid passivation layer.^[^ ^35^ ^]^ However, in situ Scanning Tunneling Microscope (STM) studies following Mg plating/stripping in the Mg(AlCl_2_)BuEt/THF electrolyte did not support this statement.^[^ ^204^ ^]^ Further analysis using in situ FTIR and XPS spectra confirmed the absence of passivating surface films in EtMgCl, BuMgCl, PhMgCl/THF, Mg(AlCl_2_)BuEt/THF, and Mg(BPh_2_Bu_2_)_2_/THF solutions.^[^ ^36^, ^228^ ^]^ In summary, these electrolytes enable effective Mg plating/stripping, reduced overpotentials during Mg deposition, and good electrochemical windows.

#### 4.2.2 All‐Phenyl Complex Electrolyte

**Figure 11:**
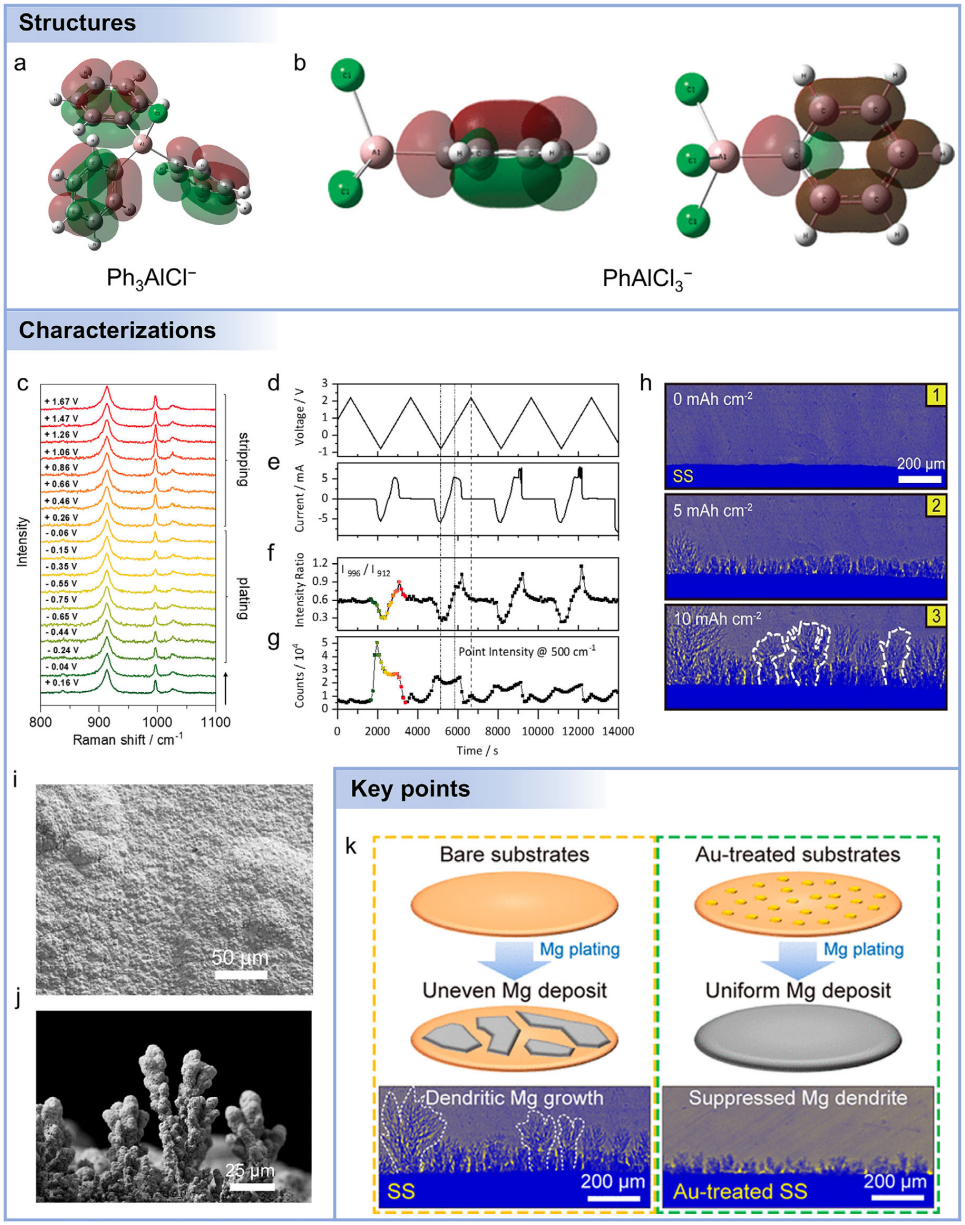
Visualization of the HOMO for a) Ph_3_AlCl^−^/THF and b) PhAlCl_3_^−^/THF. Reproduced with permission.^[^ ^229^ ^]^ Copyright 2011, American Chemical Society. c) Operando Raman spectra at different cell voltages. d) Voltage and e) current variations with cycling time during operando experiment. Raman spectroscopy metrics derived from spectra obtained during operando experiments showing f) the intensity ratio of the 996/912 cm^−1^ peak and g) point intensity at 500 cm^−1^, reflecting background intensity variations. Vertical lines are included in the second cycle to indicate the position of maximum voltage (dashed line), maximum current (dotted line), and minimum voltage and current (dash‐dot line). Reproduced with permission.^[^ ^230^ ^]^ Copyright 2023, American Chemical Society. h) Cross‐sectional images of Mg surface at a current density of 10 mA cm^−2^ by operando X‐ray microscopy. Each image was captured at three stages of 0 (OCV), 5, and 10 mAh cm^−2^ as indicated by colored boxes. Ex situ SEM images after the Mg depositions at i) 2 mA cm^−2^ and j) 10 mA cm^−2^. k) Schematic diagram of Mg deposition on different current collectors. Reproduced with permission.^[^ ^231^ ^]^ Copyright 2021, American Chemical Society.

Although the first‐generation DCC electrolyte demonstrates good reversibility in Mg stripping and plating, its oxidation stability remains a challenge due to its susceptibility to β‐H elimination reactions. To address this issue, Aurbach et al. developed second‐generation APC electrolytes in 2008 by reacting PhMgCl with AlCl_3_ at a 2:1 molar ratio in THF.^[^ ^182^ ^]^ In this system, the Al–C (phenyl) bond is stronger than the Al–C (alkyl) bond, improving the stability. The main reaction products include Ph*_x_* MgCl_2‐_ *_x_* and Ph*_y_* AlCl_3‐_ *_y_* with active species in the APC electrolyte consisting of MgCl^+^, Mg_2_Cl_3_^+^, AlPh_4_^−^, and AlPh_4‐_ *_n_* Cl*_n_* (*n* = 1–3), as shown in **Figure** **11**a,b. These species were confirmed using a combination of Raman spectroscopy, DFT calculations, NMR, and single‐crystal X‐ray Diffraction (XRD).^[^ ^229^ ^]^ As expected, the molar ratio of PhMgCl to AlCl_3_ significantly influences the electrolyte composition and the distribution of active species. When the molar ratio of PhMgCl to AlCl_3_ is 4:3, the main reactions are:

(23) $$\begin{array}{ccc} 3\mathrm{AlC}l_{3}+4\mathrm{PhMgCl} & \to & 2{\mathrm{Ph}}_{2}\mathrm{AlC}{l_{2}}^{-}+0.5{\mathrm{Al}}_{2}{\mathrm{Cl}}_{6}\\ & & +3\mathrm{MgC}l^{+}+\mathrm{MgC}l_{2}\left(>95\%\right) \end{array}$$

(24) $$3\mathrm{AlC}l_{3}+4\mathrm{PhMgCl}\to Ph_{4}Al^{-}+Al_{2}Cl_{6}+\mathrm{MgC}l^{+}+3\mathrm{MgC}l_{2}\left(>5\%\right)$$

When the molar ratio of PhMgCl to AlCl_3_ is 1:1, the main reactions are:

(25) $$2\mathrm{AlC}l_{3}+2\mathrm{PhMgCl}\to {\mathrm{Ph}}_{2}\mathrm{AlC}{l_{2}}^{-}+0.5{\mathrm{Al}}_{2}{\mathrm{Cl}}_{6}+\mathrm{MgC}l^{+}+\mathrm{MgC}l_{2}\left(>95\%\right)$$

(26) $$2\mathrm{AlC}l_{3}+2\mathrm{PhMgCl}\to {\mathrm{Ph}}_{4}{\mathrm{Al}}^{-}+\mathrm{AlC}l_{3}+2\mathrm{MgC}l_{2}\left(<5\%\right)$$

When the molar ratio of PhMgCl to AlCl_3_ is 3:2, the main reaction is:

(27) $$4\mathrm{AlC}l_{3}+6\mathrm{PhMgCl}\to 2{\mathrm{Ph}}_{3}\mathrm{AlC}l^{-}+{\mathrm{Al}}_{2}{\mathrm{Cl}}_{6}+2\mathrm{MgC}l^{+}+4\mathrm{MgC}l_{2}$$

At a molar ratio of PhMgCl to AlCl_3_ of 2:1, the main reactions are:

(28) $$\mathrm{AlC}l_{3}+2\mathrm{PhMgCl}\to {\mathrm{Ph}}_{2}\mathrm{AlC}{l_{2}}^{-}+\mathrm{MgC}l^{+}+\mathrm{MgC}l_{2}\left(\mathrm{or}{\mathrm{Mg}}_{2}{{\mathrm{Cl}}_{3}}^{+}\right)$$

(29) $$\begin{array}{ccc} 2\mathrm{AlC}l_{3}+4\mathrm{PhMgCl} & \to & \mathrm{PhMgCl}+\mathrm{AlP}{h_{4}}^{-}\\ & & +0.5{\mathrm{Al}}_{2}{\mathrm{Cl}}_{6}+\mathrm{MgC}l^{+}+3\mathrm{MgC}l_{2} \end{array}$$

Consequently, the molar ratio of PhMgCl to AlCl_3_ strongly affects the electrochemical performance. Ratios such as 4:3, 1:1, 3:2, and 2:1 led to different dominant species (e.g., Ph_2_AlCl_2_^−^, Ph_4_Al^−^, Ph_3_AlCl^−^) and varying Mg^2+^ complexes ([Mg_2_(µ‐Cl)_3_]^+^, MgCl^+^, MgCl_2_). Overpotential measurements and cycling efficiencies demonstrate that Mg plating/stripping reversibility, current density, and overpotential depend sensitively on these ratios. Electrolytes with Mg:Al ≈2:1 (the concentration of Mg is 0.8 M) achieve nearly 100% Coulombic efficiency after proper conditioning, whereas ratios above 2:1 or below 1:1 with different concentrations exhibit decreased performance due to precipitation of MgCl_2_ or formation of AlCl_4_^−^, respectively.

Operando Raman spectroscopy provided direct insight into the electrolyte dynamics of APC electrolyte during Mg plating and stripping.^[^ ^230^ ^]^ As shown in Figure 11c–f, the [AlPh_4_]^−^ band at 996 cm^−1^ exhibits a pronounced decrease in relative intensity during Mg deposition and recovers upon stripping, closely following the current response. Notably, the spectral changes lag behind the electrochemical signal by ≈200 s, giving rise to characteristic hysteresis loops around zero current (Figure 11d,e). This behavior is attributed to migration–driven redistribution of (AlPh_4_)^−^ anions at the electrode surface, where electrostatic repulsion during plating depletes the local concentration, while stripping restores it. In contrast, the THF solvent band at 1028 cm^−1^ shows only minor variations, underscoring the dominant role of anion transport in the interfacial response. At lower frequencies, subtle changes appear near 210 cm^−1^ (Mg–Cl vibration) and in the 250–400 cm^−1^ region, although interpretation is limited by signal‐to‐noise. More strikingly, the raw spectra reveal a broad baseline feature centered at ≈500 cm^−1^ that evolves periodically with cycling (Figure 11g). This background intensity rises sharply during plating, reaches a maximum at the most negative potential (−0.75 V), and gradually decreases, returning to baseline only after stripping is complete and current ceases. The effect is fully reversible but diminishes in magnitude with repeated cycles. Taken together, the electrochemical data (Figure 11d–e) and Raman‐derived metrics (Figure 11f,g) highlight the coupling between interfacial electron transfer and local ionic redistribution.

When using the APC electrolyte, Mg deposition typically does not lead to dendrite formation on the Mg surface. However, Kwak et al. demonstrated that Mg surfaces are capable of forming dendrites at high current densities.^[^ ^231^ ^]^ At 2 mA cm^−2^, minimal dendritic growth is observed. In contrast, at 10 mA cm^−2^, noticeable dendritic structures emerge (Figure 11h).^[^ ^232^ ^]^ Ex situ SEM images further illustrate this phenomenon. At 2 mA cm^−2^, only tiny spherical Mg seeds are observed (Figure 11i). However, at 10 mA cm^−2^, Mg dendrites appear sporadically, forming micron‐sized branches that predominantly grow upward (Figure 11j). To mitigate dendrite formation, Au magnesiophilic sites have been introduced on various metal substrates (Mg, Cu, and SS), effectively suppressing needle‐like dendrite growth (Figure 11k). This modified growth process decreases nucleation polarization, thereby limiting Mg dendrite formation and lowering the risk of short circuit. The ability of APC to suppress dendrite formation at low current densities is also closely related to its unique ionic structure. The dynamic arrangement of these ionic species, including [Mg_2_(µ‐Cl)_3_(THF)_6_]^+^ and Al‐centered chloroaluminate anions, facilitates uniform Mg^2+^ transport and deposition across the electrode surface, minimizing local ion concentration gradients that typically trigger dendrite nucleation. Furthermore, bridging chloride ions reduces the Mg^2+^ desolvation barrier, promoting homogeneous plating and enhancing overall deposition stability. This structure–performance relationship highlights the critical role of electrolyte speciation in controlling interfacial phenomena and ensuring dendrite‐free Mg deposition.^[^ ^39^, ^187^, ^205^, ^213^, ^214^ ^]^

Despite their ability to support reversible Mg plating/stripping, magnesium organo‐haloaluminate‐based electrolytes, particularly DCC and APC electrolytes, still exhibit limited compatibility with cathode materials, necessitating further optimization.

### 4.3 Inorganic Salt‐Based Electrolytes

**Figure 12:**
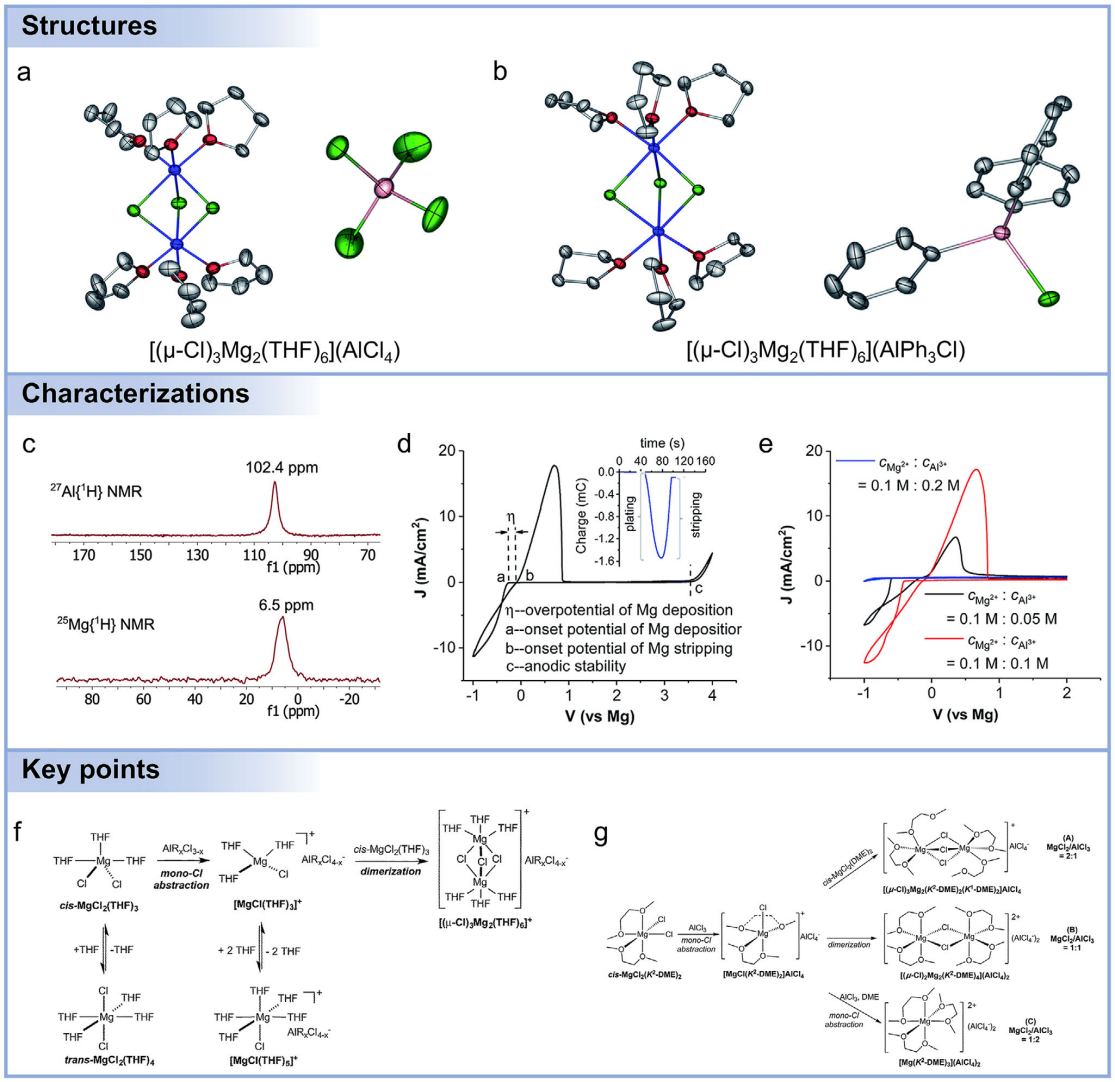
Molecular structures of a) [(µ‐Cl)_3_Mg_2_(THF)_6_](AlCl_4_) and b) [(µ‐Cl)_3_Mg_2_(THF)_6_](AlPh_3_Cl) (Mg, blue; Cl, green; Al, pink; C, gray; O, red). c) ^27^Al{^1^H} NMR and ^25^Mg{^1^H} NMR spectra of the MgCl_2_‐AlCl_3_ electrolyte recorded in THF at 22°C. Reproduced with permission.^[^ ^209^ ^]^ Copyright 2014, Royal Society of Chemistry. d) Cyclic voltammetry (CV) curves of the freshly prepared MgCl_2_–AlCl_3_/DME electrolytes with different MgCl_2_:AlCl_3_ ratios obtained at 30°C. CV curves of 0.1 M MgCl_2_–AlCl_3_/DME (0.1 M/0.1 M) with labels of onset potentials of Mg deposition (a) and stripping (b), overpotential (η), and anodic stability (c). Inset gives the plot of charge over time for the deposition and stripping. e) CV curves of MgCl_2_/AlCl_3_ = 0.1 M/0.05 M, 0.1 M/0.1 M, and 0.1 M/0.2 M in DME. Reproduced with permission.^[^ ^234^ ^]^ Copyright 2017, Royal Society of Chemistry. f) Proposed reaction sequences to form [(µ‐Cl)_3_Mg_2_(THF)_6_]^+^ via mono‐Cl^−^ abstraction. Reproduced with permission.^[^ ^209^ ^]^ Copyright 2014, Royal Society of Chemistry. g) The reaction scheme of the MgCl_2_–AlCl_3_/DME electrolytes at different reactant ratios. Reproduced with permission.^[^ ^234^ ^]^ Copyright 2017, Royal Society of Chemistry.

Common inorganic Mg salts like MgCl_2_, Mg(ClO_4_)_2_, Mg(BF_4_)_2_, MgSO_4_, Mg(PF_6_)_2_, and Mg(NO_3_)_2_ have been widely used in Mg battery electrolytes. However, Schechter et al. reported that reversible Mg plating/stripping is challenging in electrolytes containing ClO_4_^−^ and BF_4_^−^.^[^ ^184^ ^]^ Among these salts, MgCl_2_ shows significant potential for Mg battery electrolytes. Nevertheless, its low solubility in common organic solvents like THF imposes a major challenge for efficient Mg plating/stripping.^[^ ^233^ ^]^ One effective approach to enhance the solubility of MgCl_2_ in organic solvents is the use of additives. In particular, combining MgCl_2_ with AlCl_3_ leads to the formation of electrochemically active species with improved reactivity. For instance, the MgCl_2_–AlCl_3_/THF electrolyte can generate electrochemically active [Mg_2_(µ‐Cl)_3_(THF)_6_]^+^, [Mg_2_(µ‐Cl)_2_]^2+^, MgCl_2_(THF)_4_, [MgCl(THF)_5_]^+^, and MgCl_2_(THF)_3._^[^ ^211^ ^]^ As shown in **Figure** **12**a, the molecular structure of the electrolyte consists of the Mg‐dimer monocation, [(µ‐Cl)_3_Mg_2_(THF)_6_]^+^, and AlCl_4_^−^. The cation adopts a pseudo‐D3h symmetry with three bridging Cl^−^ ligands between two Mg^2+^ and three terminal THF ligands on each Mg^2+^. Each Mg^2+^ is arranged in an octahedral configuration. The ^27^Al{^1^H} NMR spectrum of the resulting solution shows a resonance at 102.4 ppm, confirming the formation of AlCl_4_^−^(Figure 12c). The ^25^Mg{^1^H} NMR spectrum displays a singlet at 6.5 ppm, consistent with the formation of Mg^2+^ species (Figure 12b), compared to the 7.9 ppm resonance observed for MgCl_2_ in THF. The effective components include [Mg_2_(µ‐Cl)_3_(THF)_6_]^+^ cations, and their formation process is depicted in Figure 12f. Additionally, AlCl_4_^−^, AlPh_3_Cl^−^, and AlEtCl_3_^−^ anions are observed. Despite having a high chlorine concentration and poor solvent solubility, the electrolyte with AlCl_4_^−^ shows the highest oxidative stability among all the tested electrolytes.^[^ ^209^ ^]^

Doe et al. obtained MACC electrolytes by reacting MgCl_2_ with AlCl_3_ in THF, G1, and G4 solvents.^[^ ^187^ ^]^ The oxidative potential of the resulting (MgCl_2_)_2_–AlCl_3_/DME electrolyte is 3.1 V vs Mg^2+^/Mg, with Coulombic efficiencies exceeding 99%. Regarding the structure and composition of [(µ‐Cl)_3_Mg_2_(THF)_6_](AlR*_x_* Cl_4−_ *_x_*) (*x* = 0–3, R = alkyl or aryl group), the dimer salts can be deconvoluted into three simple synthons: two equivalents of MgCl_2_, six equivalents of THF, and one equivalent of an Al Lewis acid, AlR*_x_* Cl_3−_ *_x_*. Liu et al. reacted MgCl_2_ with different aluminum‐based Lewis acids (AlCl_3_ AlEtCl_2_, AlPh_3,_ and AlCl_3_) to obtain a series of electrolytes,^[^ ^209^ ^]^ which were more atom‐efficient (free of undesired byproducts), more cost‐effective and operationally easier. The 0.04 M (MgCl_2_)_2_–AlCl_3_/THF electrolyte demonstrated high oxidative stability (3.4 V vs Mg^2+^/Mg), an ionic conductivity of 0.26 mS cm^−1^, and excellent electrochemical reversibility (Coulombic efficiency up to 100%), also exhibiting dendrite‐free, smooth and uniform Mg deposition on Pt. However, the concentration of MgCl_2_ in THF is lower than that in the DME solvent.^[^ ^187^ ^]^

Moreover, changing the ratio of MgCl_2_ to AlCl_3_ resulted in decreased Mg plating/stripping current densities and increased Mg plating/stripping overpotentials (> 0.6 V). When the Mg:Al ratio exceeded 2:1, insoluble MgCl_2_ appeared in the electrolyte. Conversely, when the Mg:Al ratio was less than 2:1, AlCl_3_ would react with the active species [Mg_2_(µ‐Cl)_3_]^+^ to form AlCl_4_^−^, resulting in decreased ion concentrations in the electrolyte and reduced rates of Mg deposition.^[^ ^235^ ^]^ As seen in Figure 12d,e, the 1 : 1 MgCl_2_–AlCl_3_/DME electrolyte demonstrated unparalleled capabilities in Mg plating/stripping, such as reversibility (up to 100% CE), ionic conductivity (>8.5 mS cm^−1^), and good anodic stability (3.5 V vs Mg^2+^/Mg).^[^ ^234^ ^]^ A comprehensive reaction scheme for the MgCl_2_–AlCl_3_/DME electrolytes at different reactant ratios is proposed in Figure 12g. Nevertheless, freshly produced solutions do not initially show reversible Mg plating and stripping, regardless of the starting Mg:Al proportion. Specifically, electrolytes with 1:1 and 2:1 Mg:Al ratios showed reversible Mg plating and stripping after enough cycling,^[^ ^236^ ^]^ needing an electrolytic conditioning. This composition of cycled electrolytes approaches a Mg:Al ratio of (2.59 ± 0.08):1 in both scenarios. According to this discovery, irreversible deposition of Al or Mg takes place until the electrolyte reaches the desirable composition. However, the MgCl_2_–AlCl_3_/DME electrolyte with a Mg:Al ratio of (2.7 ± 0.1):1 could not be conditioned even after extensive cycling, and the Mg:Al ratio diverges to 3.5:1 after cycling. Additionally, the MgCl_2_–AlCl_3_/DME electrolyte with an Mg:Al ratio of 2.6:1 differs from the crystallized electrolyte, [Mg_2_(µ‐Cl)_3_(THF)_6_][AlCl_4_], where a 2:1 Mg:Al ratio is suggested. It is most likely that neither the electroactive species nor the overall composition of the electrolytes is reflected in the crystallized electrolyte.

Apart from the need for an electrolytic conditioning step to initiate the MACC electrolyte, another drawback is the low Mg plating/stripping rates, which range from 1.8 to 2.0 mA cm^−2^ at a CV scan rate of 5 mV s^−1^. See et al. evaluated the concentration effect on the Mg electrodeposition current densities using THF.^[^ ^235^ ^]^ As revealed, increases in concentrations have a substantial impact on the conditioning procedure in addition to producing a noticeable shift in the Mg electrodeposition metrics. Significantly larger plating/stripping current densities, substantially easier plating/stripping kinetics, a much lower deposition overpotential (60 mV), and quicker electrolytic conditioning were the outcomes of increasing the component salt concentration in the MACC. MACC consisted of 300 mM MgCl_2_ + 150 mM AlCl_3_ in THF achieved cathodic current densities up to ≈7.5 mA cm^−2^ at 5 mV s^−1^, as well as a high anodic stability of >3.2 V vs Mg^2+^/Mg and Coulombic efficiency of >99%.

One likely scenario is that AlCl_2_^+^‐containing solutions of MgCl_2_–AlCl_3_/G4 corroded Mg metal, leaving Al deposits through a spontaneous cementation reaction mechanism.^[^ ^38^ ^]^ AlCl_2_^+^ is reduced to metallic Al, whereas Mg is oxidized and dissolved as Cl^–^ coordinated Mg^2+^ species. Since corrosion of Mg only occurs in AlCl_2_^+^‐containing electrolytes, the “conditioning” of AlCl_2_^+^–free solutions would primarily be associated with the elimination of reducible impurities. When MgBu_2_ (10 mM) was added to the AlCl_2_^+^–free 0.2 M MgCl_2_–AlCl_3_/G4 solution, a “conditioning”‐free Mg deposition and dissolution behavior was observed. The 1^st^ cycle of Mg plating and stripping proceeded at very low overpotentials and showed a high Coulombic efficiency of 87%. At the 100th cycle, the Coulombic efficiency further increased to 94%. Hoon Ha et al. attempted to catalyze Mg metal dissolution using CrCl_3_ in the presence of THF and AlCl_3_, forming an electrolyte consisting entirely of inorganic species,^[^ ^237^ ^]^ where Coulombic efficiency of ≈100% was obtained for Mg plating/stripping in the first cycle, with a stable electrochemical window up to 3.1 V vs Mg^2+^/Mg. It is clear that adding CrCl_3_ during electrolyte preparation generates stable ionic species for charge transport, limiting the need for complex electrolytic conditioning due to the high initial Mg to Al ratio (≈13) in the electrolyte. Unfortunately, Cr is tetrogenic and poses multiple real‐life problems.

### 4.4 Weakly Coordinating Anion‐Based Electrolytes

Recent advances also highlight the use of Mg salts such as Mg(TFSI)_2_, Mg(HMDS)_2_, and Mg[B(hfip)_4_]_2_, which incorporate large, weakly coordinating anions to further enhance the reversibility of Mg anodes and electrochemical stability of electrolytes. Their large molecular architectures and minimized ion‐pairing tendencies fundamentally modify the solvation environment, also enabling enhanced Mg^2+^ mobility and improved electrolyte stability, even in chlorine‐containing systems. These salts have been extensively applied across various electrolyte formulations, demonstrating superior anodic stability, ionic conductivity, and chemical compatibility.

#### 4.4.1 Mg(TFSI)_2_‐Based Electrolyte

Mg(TFSI)_2_ (Mg[N(SO_2_CF_3_)_2_]_2_) has been widely used in the development of Mg battery electrolytes due to its high solubility in organic solvents, good ionic conductivity, and chemical stability.^[^ ^238^, ^239^, ^240^ ^]^ However, its reductive decomposition leads to the formation of a passivation layer composed of Mg(OH)_2_, MgS, and MgO, causing a Mg stripping overpotential of ≈2 V.^[^ ^238^ ^]^ Moreover, electrolytes containing only Mg(TFSI)_2_ often exhibit Coulombic efficiencies below 60%, significantly limiting their practical application.^[^ ^238^ ^]^ To address these issues, researchers have explored the introduction of various additives to improve electrolyte performance.^[^ ^72^, ^241^ ^]^ Notably, adding MgCl_2_ to the Mg(TFSI)_2_ electrolyte can significantly enhance Coulombic efficiency and reduce the overpotential for Mg plating/stripping. This improvement is primarily due to Cl^−^ ions, which not only interact with Mg^2+^ and the electrolyte solvent to form unique complexes but also adsorb onto the Mg electrode surface, preventing unwanted surface reactions and inhibiting the formation of passivation layers.

**Figure 13:**
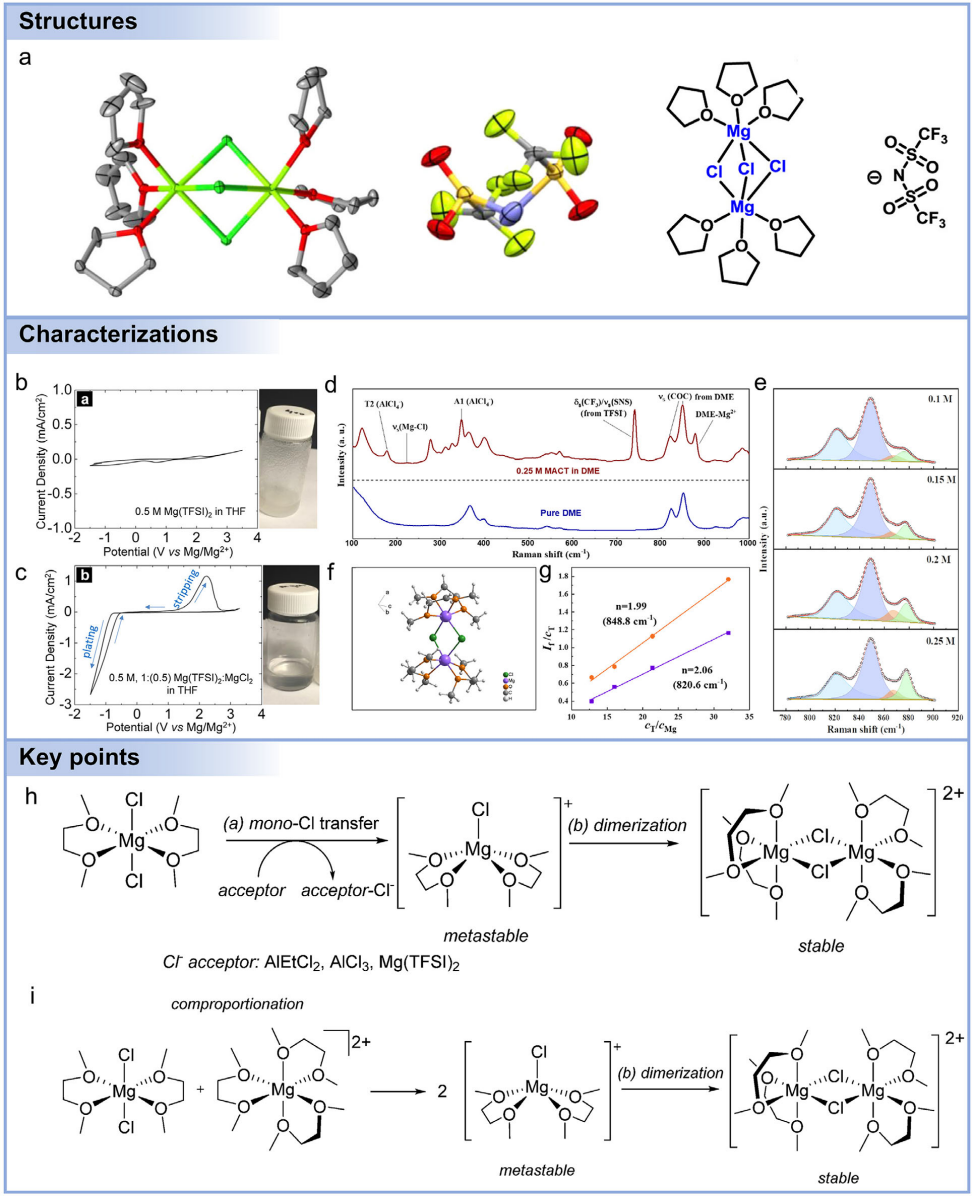
a) Displacement of ellipsoid representation of [Mg_2_Cl_3_(THF)_6_](TFSI). CV curves of Mg(TFSI)_2_‐based electrolyte: b) 0.5 M Mg(TFSI)_2_ in THF, where Mg(TFSI)_2_ is not fully dissolved (see picture on right); c) 0.5 M Mg(TFSI)_2_ with addition of MgCl_2_ at a mixing ratio of 1:0.5 in the THF solvent (molar ratio referenced with the TFSI salt). A clear and homogeneous solution is observed (see picture on right). Reproduced with permission.^[^ ^191^ ^]^ Copyright 2016, American Chemical Society. d) Raman spectra of MACT/DME and pure DME. e) Deconvoluted Raman spectra and comparison of MACT/DME at different concentrations. f) Crystal structure of the MACT/DME electrolyte. g) Fitting curves of (I_t_/C_t_)‐(C_t_/C_Mg_) at 820.6 and 848.8 cm^−1^. Reproduced with permission.^[^ ^214^ ^]^ Copyright 2021, American Chemical Society. h) Proposed reaction path to [Mg_2_(µ‐Cl)_2_(DME)_4_]^2+^ via mono‐Cl transfer. i) Proposed reaction path to [Mg_2_(µ‐Cl)_2_(DME)_4_]^2+^ via comproportionation. Reproduced with permission.^[^ ^37^ ^]^ Copyright 2015, Royal Society of Chemistry.

For example, Sa et al. prepared a 0.25 M [Mg(TFSI)_2_]_2_–MgCl_2_/THF electrolyte,^[^ ^191^ ^]^ successfully resolving its structure using single‐crystal XRD. Their analysis revealed the presence of an ion pair, [Mg_2_Cl_3_(THF)_6_]^+^(TFSI)^−^ (**Figure** **13**a). The cation structure, [Mg_2_Cl_3_(THF)_6_]^+^, is composed of a dimagnesium core bridged by three chlorides, further coordinated with six THF molecules. More importantly, the active [Mg_2_Cl_3_(THF)_6_]^+^ species at elevated concentrations assist in improving the full cell performance. An inhomogeneous solution was observed for a 0.5 M Mg(TFSI)_2_/THF electrolyte (Figure 13b), even after 48 h of stirring. When this combination was used as the electrolyte, no reversible Mg plating/stripping was seen. By contrast, the Mg(TFSI)_2_/THF system appears as a clear and uniform solution after adding MgCl_2_, and the resulting electrolyte allows for reversible Mg plating/stripping (Figure 13c).

The primary species present in the Mg(TFSI)_2_–MgCl_2_/DME electrolyte have also been identified.^[^ ^37^ ^]^ Due to the large size and weak Lewis basicity of TFSI^−^, the predominant solvated structure in solution is [Mg(DME)_3_]^2+^. Upon adding MgCl_2_ to the Mg(TFSI)_2_/DME electrolyte, [MgCl(DME)_2_]^+^ cations form. However, the stability of [MgCl(DME)_2_]^+^ is relatively low in the electrolyte, leading to dimerization and the subsequent formation of stable and electrochemically active [Mg_2_(µ‐Cl)_2_(DME)_4_]^2+^. At equilibrium, the solution contains a dynamic mixture of [MgCl(DME)_2_]^+^, [Mg_2_(µ‐Cl)_2_(DME)_4_]^2+^, and [Mg(DME)_3_]^2+^.^[^ ^191^ ^]^ In contrast, when the Mg(TFSI)_2_–MgCl_2_/THF electrolyte is prepared using THF, the predominant structure shifts to [Mg_2_(µ‐Cl)_3_(THF)_6_]^+^. The addition of MgCl_2_ further enhances the electrochemical performance of the Mg(TFSI)_2_–MgCl_2_/THF electrolyte. The reaction pathways leading to [Mg_2_(µ‐Cl)_2_DME_4_]^2+^ via mono‐Cl transfer and comproportionation have also been proposed (Figure 13h,i). Shterenberg et al. further demonstrated that Mg(TFSI)_2_/DME electrolyte could dissolve a substantial amount of MgCl_2_,^[^ ^212^ ^]^ partially overcoming the issue of low MgCl_2_ solubility in ether solvents. They speculated that the increased solubility might be attributed to the formation of Mg/Cl/TFSI complexes upon the introduction of Mg(TFSI)_2_. Additionally, the resulting Mg(TFSI)_2_/MgCl_2_/DME electrolyte exhibited enhanced electrochemical performance, achieving 98% reversibility, a deposition overpotential of 0.2 V, and an electrochemical window of 3.1 V vs Mg^2+^/Mg. It is suggested that chloride anions may adsorb onto Mg surfaces, inhibiting the reduction of TFSI^−^ anions in these systems. While MgCl_2_ may also serve as an effective additive in other solvents such as di‐ and tri‐glyme, its solubility remains limited. Furthermore, Sa et al. attempted to prepare a 0.5 M Mg(TFSI)_2_/THF electrolyte, but the solution became inhomogeneous after 48 h of stirring.^[^ ^191^ ^]^ No reversible Mg plating/stripping was achieved. Nevertheless, the addition of MgCl_2_ led to the successful formation of a 0.25 M (Mg(TFSI)_2_)_2_–MgCl_2_/THF electrolyte, which enabled reversible Mg plating/stripping.

To mitigate DME decomposition while preserving its beneficial properties, Yue et al. added THF as a non‐fluorinated weakly coordinating co‐solvent into the Mg(TFSI)_2_–MgCl_2_/DME solution, leveraging the higher solubility of DME for Mg(TFSI)_2_.^[^ ^171^ ^]^ The resulting Mg(TFSI)_2_–(MgCl_2_)_2_/DME–THF (75 vol% THF) electrolyte demonstrated a significantly improved Coulombic efficiency of 98.8%, compared with Mg(TFSI)_2_–(MgCl_2_)_2_/DME (95.2%) and Mg(TFSI)_2_–(MgCl_2_)_2_/THF (90.3%). This enhancement is attributed to the formation of easily‐migrating DME–rich solvation shells surrounded by weakly coordinating THF, which facilitates the development of a stable SEI without promoting excessive ion association. This protective film effectively suppresses electrolyte‐induced parasitic reactions, leading to improved electrochemical stability. Additionally, in situ optical microscopy (OM) and in situ atomic force microscopy (AFM) were used to investigate the dynamic nucleation, growth, and stripping of Mg at the electrode/electrolyte interface in the Mg(TFSI)_2_–MgCl_2_/DME electrolyte.^[^ ^242^ ^]^ Observations revealed that the slow Mg deposition and its tendency to desorb from the electrode during dissolution could be attributed to the point contact between the deposited Mg and the electrode. In contrast, the Mg(HMDS)_2_–MgCl_2_–AlCl_3_/G3 electrolyte exhibited a significantly higher Mg nucleation rate, faster deposition dynamics, and superior reversibility.

Despite the improvements in the Mg(TFSI)_2_–MgCl_2_/DME electrolyte, the decomposition of dissociated TFSI^−^ anions remains a challenge, as it can passivate the Mg surface. Fan et al. identified the formation of [Mg_3_(µ‐Cl)_4_(DME)*_m_* (TFSI)_2_] (*m*  =   3, 5) inner‐shell solvation clusters, which decompose easily, leading to the development of a MgO– and MgF_2_–rich SEI.^[^ ^54^ ^]^ This passivation layer contributes to uneven Mg deposition and poor electrochemical performance. To counteract this issue, the tri(2,2,2‐trifluoroethyl) borate (TFEB), an electron‐deficient additive, was introduced to inhibit the formation of electron‐rich MgO and MgF_2_ in the SEI. The addition of TFEB significantly improved the electrolyte performance, enabling stable cycling for over 400 h with a low average overpotential of ≈150 mV and a high Coulombic efficiency of 97.87% even after 200 cycles.

Introducing AlCl_3_ to Mg(TFSI)_2_/MgCl_2_ solutions has been another effective strategy to enhance the electrochemical performance of Mg anodes.^[^ ^213^ ^]^ The resulting 0.03 M (MgCl_2_)_2_–AlCl_3_–Mg(TFSI)_2_/THF (MACT/THF) electrolyte offered a Coulombic efficiency of 93%, which is higher than that of 0.03 M (MgCl_2_)_2_‐AlCl_3_/THF (89 %). This hybrid electrolyte also exhibited remarkable tolerance to air, water, and impurities. Crystalline species isolated from the hybrid electrolyte include [Mg_2_(µ‐Cl)_3_(THF)_6_](AlCl_4_) and [Mg_2_(µ‐Cl)_3_(THF)_6_](TFSI). The addition of Mg(TFSI)_2_ likely contributed to converting more inert MgCl_2_ into the electrochemically active species [Mg_2_(µ‐Cl)_3_(THF)_6_]^+^, eliminating the need for a conditioning process. The 0.03 M MACT/THF electrolyte maintained its activity even in the presence of water, with tested water contents of 500, 1000, and 2000 ppm. The best performance was achieved with 1000 ppm water content (0.03 M MACT/THF–1000), where the coordination‐hydrolysis process increased the effective electrolyte concentration from 0.03 to 0.21 M.^[^ ^243^ ^]^ In 0.03 M MACT/THF‐1000, the active Mg species was identified as [Mg_2_(µ‐Cl)_3_(THF)_6_]^+^. However, at a higher concentration of 0.18 M MACT/THF–2000, a complex of [MgCl(THF)_5_]^+^ appeared. This electrolyte exhibited exceptional stability, with a CE exceeding 99% over 800 cycles. Moreover, it provided stable Mg plating/stripping with overpotentials of ≈240 mV, even at a current density of 4 mA cm^−2^. These findings suggest that rather than passivating the Mg anode, the 0.18 M MACT/THF–2000 electrolyte might actively enhance its performance.

However, the limited solubility of MgCl_2_ in THF significantly limits ionic conductivity (up to 0.28 mS cm^−1^) and results in slow Mg dynamics, leading to poor rate capabilities and cycling stability. To address this issue, Lanlan et al. developed a 0.25 M (MgCl_2_)_2_–AlCl_3_–Mg(TFSI)_2_/DME (MACT/DME) electrolyte,^[^ ^214^ ^]^ which demonstrated significantly improved performance. This electrolyte exhibited high ionic conductivity (6.8 mS cm^−1^), low Mg plating/stripping overpotentials of ≈120 mV, and a Coulombic efficiency of 96%. Single‐crystal XRD and Raman spectroscopy (Figure 13d–g) revealed that the dominant electroactive species in MACT/DME consists of two Mg atoms bridged by two Cl atoms, with each Mg atom additionally coordinated by two bidentate DME molecules, forming a hexahedral [Mg_2_(µ‐Cl)_2_(DME)_4_]^2+^ complex. This structure is accompanied by (AlCl_4_)^−^ and TFSI^–^ species, which contribute to the enhanced electrochemical properties of the electrolyte.

In conclusion, the addition of MgCl_2_ and AlCl_3_ allows Mg(TFSI)_2_‐based electrolytes to undergo controlled structural modifications, facilitating the reduction of Mg^2+^. Furthermore, the presence of additional Cl^−^ anions modulates the Mg surface, effectively lowering the overpotential for Mg plating/stripping and improving overall electrolyte performance.

#### 4.4.2 Mg(HMDS)_2_‐Based Electrolytes

**Figure 14:**
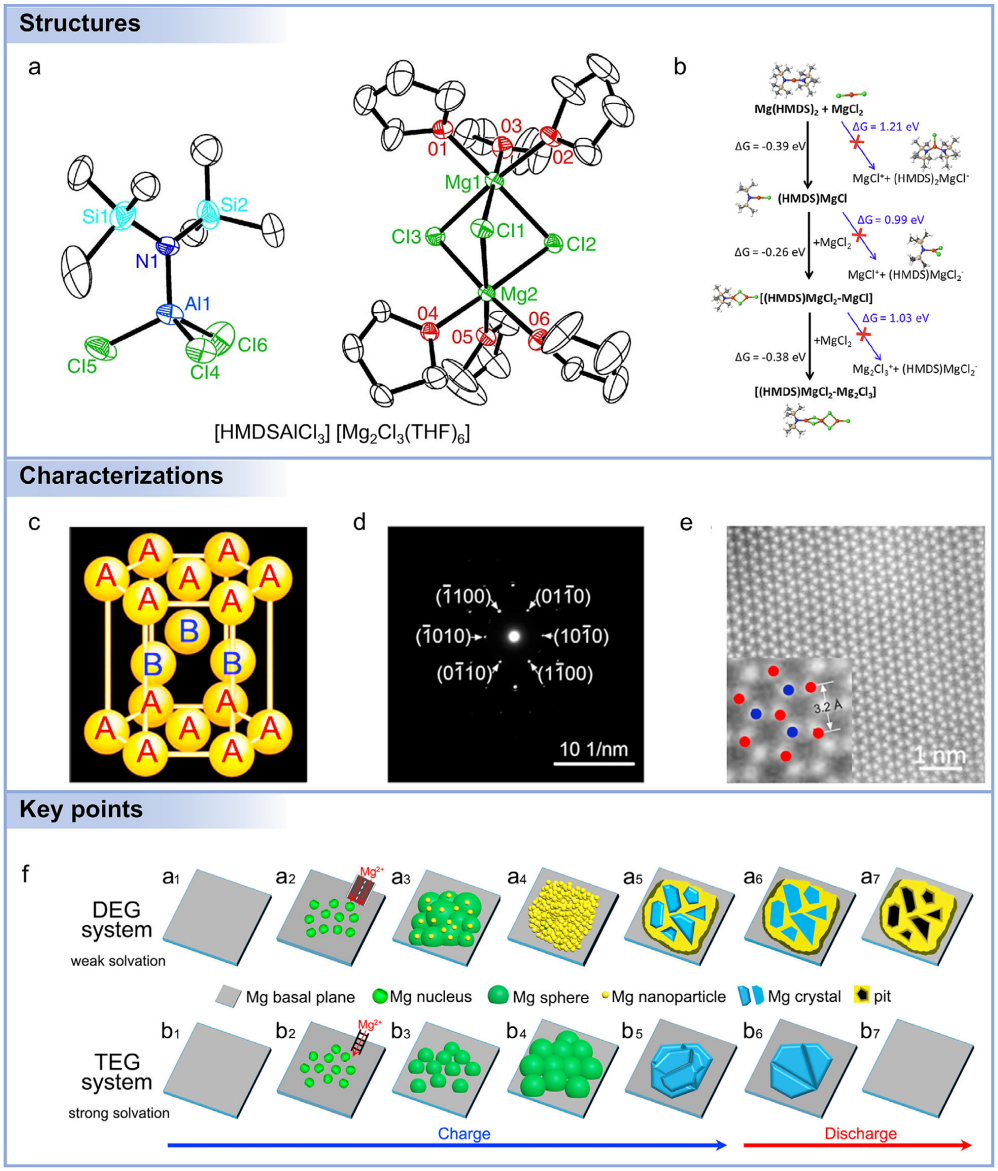
a) ORTEP plot (25% thermal probability ellipsoids) of [Mg_2_Cl_3_(THF)_6_](HMDSAlCl_3_). Hydrogen atoms, THF of crystallization and second component of disorder are omitted for clarity. Reproduced with permission.^[^ ^145^ ^]^ Copyright 2011, Springer Nature. b) Main reaction pathway (in black arrow) and other less possible reactions we calculated. Reaction free energies (corresponding to 1 mole of each reactant) of these steps are reported. Reproduced with permission.^[^ ^215^ ^]^ Copyright 2015, Royal Society of Chemistry. c) Schematic illustration of hexagonal‐close‐packed (HCP) Mg crystal atomic structure. d) Selected area electron diffraction (SAED) of a crystal. e) Annular bright field scanning transmission electron microscopy (ABF‐STEM) image of Mg crystal viewed from the [0001] direction. f) Schematic illustration of interfacial processes of the Mg plating/stripping in G2 system (a1‐a7) and G4 system (b1‐b7). (a1) OCP, (a2) bowl‐like Mg nucleation, (a3) Mg spheres and NPs emergence, (a4) NPs stack upon charge, (a5) crystals growth, (a6) crystals stripping, and (a7) pits emergence; (b1) OCP, (b2) bowl‐like Mg nucleation, (b3) Mg spheres emergence, (b4) Mg spheres growth, (b5) crystals growth, (b6) crystals stripping, and (b7) the complete stripping of crystals. Reproduced with permission.^[^ ^246^ ^]^ Copyright 2018, Elsevier.

The organomagnesium compound, Mg(HMDS)_2_ [HMDS = N(Si(CH_3_)_3_)_2_], possesses key properties that make it suitable for magnesium metal battery (Mg battery) electrolytes. These include strong basicity, easy solubility in nonpolar solvents, and low nucleophilicity. Inspired by the non‐nucleophilic base properties of potassium hexamethyldisilazide,^[^ ^225^ ^]^ Kim et al. developed hexamethyldisilazide magnesium chloride (HMDSMgCl)‐based electrolytes for magnesium sulfur batteries.^[^ ^145^ ^]^ This electrolyte was prepared by reacting HMDSMgCl with AlCl_3_, with optimal electrochemical performance achieved at a HMDSMgCl:AlCl_3_ molar ratio of 3:1 and a reaction time of 24 h. The obtained electrolyte demonstrated high oxidative stability (3.2 V vs Mg^2+^/Mg) and 100% Coulombic efficiency. Single‐crystal XRD analysis revealed that the electrolyte contained a cationic species composed of two octahedrally coordinated Mg centers bridged by three chlorine atoms, with three remaining coordination sites on each Mg occupied by THF molecules. The overall crystal structure of [Mg_2_(µ‐Cl)_3_(THF)_6_][HMDSAlCl_3_] is illustrated in **Figure** **14**a. The counterion consists of one HMDS ligand and three chlorine atoms tetrahedrally coordinating an Al atom. The reactions involved in the electrolyte formation are detailed in Equations (30)–(34). Experimental results showed that electrolyte crystallization improved anodic stability, likely due to the oxidation susceptibility of unreacted HMDSMgCl. Additionally, crystallization enhanced the Coulombic efficiency of Mg plating/stripping, increasing it from 95 to 100%, further solidifying its potential as a high‐performance Mg battery electrolyte.

(30) $$\left(\mathrm{HMDS}\right)\mathrm{MgCl}+\mathrm{AlC}l_{3}\to \mathrm{MgC}l^{+}+\left(\mathrm{HMDS}\right)\mathrm{MgC}{l_{3}}^{-}$$

(31) $$2\mathrm{HMDSMgCl}↔\mathrm{Mg}{\left(\mathrm{HMDS}\right)}_{2}+\mathrm{MgC}l_{2}$$

(32) $$\mathrm{MgC}l^{+}+\mathrm{MgC}l_{2}↔Mg_{2}C{l_{3}}^{+}$$

(33) $$3\mathrm{HMDSMgCl}+\mathrm{AlC}l_{3}\to Mg_{2}C{l_{3}}^{+}+\mathrm{HMDSMgC}{l_{3}}^{-}+\mathrm{Mg}{\left(\mathrm{HMDS}\right)}_{2}$$

(34) $$\mathrm{HMDSAlC}{l_{3}}^{-}+\mathrm{HMDSMgCl}\to \mathrm{HMDSAlC}{l_{2}}^{-}+\mathrm{MgC}l_{2}$$

(35) $$\mathrm{Mg}{\left(\mathrm{HMDS}\right)}_{2}+\mathrm{MgC}l_{2}↔2\left(\mathrm{HMDS}\right)\mathrm{MgCl}$$

(36) $$\left(\mathrm{HMDS}\right)\mathrm{MgCl}+\mathrm{MgC}l_{2}\to \left[\left(\mathrm{HMDS}\right)\mathrm{MgC}l_{2}\right]\left(\mathrm{MgCl}\right)$$

(37) $$\left[\left(\mathrm{HMDS}\right)\mathrm{MgC}l_{2}\right]\left(\mathrm{MgCl}\right)+\mathrm{MgC}l_{2}\to \left[\left(\mathrm{HMDS}\right)\mathrm{MgC}l_{2}\right]{\left(Mg_{2}Cl_{3}\right)}^{+}$$

Liao et al. prepared a Mg(HMDS)_2_‐(MgCl_2_)_4_/THF electrolyte, where the active species was proposed to be [(HMDS)MgCl_2_](Mg_2_Cl_3_).^[^ ^215^ ^]^ Density functional theory (DFT) calculations were employed to assess the thermodynamic feasibility of the reaction sequence in the electrolyte (Figure 14b), revealing an energetically favorable pathway. As shown in Equation (35), the reversible equilibrium between Mg(HMDS)_2_ and MgCl_2_ yields the intermediate (HMDS)MgCl with a free energy of −0.39 eV. As shown in Equation (36), transmetallation between (HMDS)MgCl and MgCl_2_ forms the [(HMDS)MgCl_2_‐MgCl] complex, an exothermic process by 0.26 eV. Equation (37) shows that the [(HMDS)MgCl_2_‐MgCl] complex could further react with MgCl_2_ and form [(HMDS)MgCl_2_‐Mg_2_Cl_3_] as the final product, with an exothermic free energy of −0.38 eV. The calculated free energies confirm that this reaction pathway, depicted by the black arrow in Figure 14b, is the most thermodynamically favorable, shedding light on the electrolyte's stability and structural evolution.

The electrochemical properties of a series of electrolytes were also investigated by adjusting the ratio of Mg(HMDS)_2_ and AlCl_3_ in the reactions.^[^ ^216^, ^244^ ^]^ The Mg(HMDS)_2_‐(AlCl_3_)_2_/THF electrolyte, synthesized with a Mg(HMDS)_2_:AlCl_3_ molar ratio of 1:2, exhibited the best electrochemical performance with an oxidative stability of 3.3 V vs Mg^2+^/Mg, an ionic conductivity of 1.7 mS cm^−1^, and a Coulombic efficiency of 98%. Additionally, a 0.9M Mg(HMDS)_2_‐(AlCl_3_)_2_‐MgCl_2_/diglyme electrolyte was synthesized,^[^ ^245^ ^]^ with active species potentially including (Mg_2_Cl_3_)^+^, (MgCl)^+^, MgCl_2_, (HMDSAlCl_3_)^−^_,_ and HMDSAlCl_2_. Liao et al. further explored a Mg(HMDS)_2_‐(MgCl_2_)_4_/THF electrolyte by reacting Mg(HMDS)_2_ and MgCl_2_ in a molar ratio of 1:4 in THF.^[^ ^215^ ^]^ In this electrolyte, Mg(HMDS)_2_ enhanced the solubility of MgCl_2_ in THF, achieving a Mg^2+^ solubility of 1.25 M. This electrolyte demonstrated an oxidative stability of 2.8 V (vs Mg^2+^/Mg) and an impressive Coulombic efficiency of 99.9%, requiring no additional conditioning or activation.

The influence of solvents G2 and G4 in the Mg(HMDS)_2_‐MgCl_2_‐AlCl_3_ electrolyte was explored using in situ AFM and optical microscope,^[^ ^246^ ^]^ revealing distinct deposition behaviors. The deposition process in both solvents begins with the formation of bowl‐like nuclei, which subsequently evolve into spherical structures. Over time, nanoparticles (NPs) progressively emerge from these deposited spheres, contributing to the transition from an amorphous to a more defined crystalline structure. Notably, regular crystals strip more efficiently than adjacent amorphous sediments, indicating that the G4‐based electrolyte facilitates better reversibility than the G2 system. Structural characterization using a spherical aberration‐corrected electron microscope confirmed the hexagonal close‐packed (HCP) Mg crystal structure (Figure 14c), viewed along the [0001] direction, as verified by the SAED pattern (Figure 14d). Aberration‐corrected annular bright‐field scanning transmission electron microscopy (ABF‐STEM) further visualized Mg atomic grids, displaying crystallographic planes (0001) and (0002), where two distinct Mg atom layers were observed (Figure 14e). The electrochemical performance of these electrolytes is closely linked to interfacial stages and dynamic transformations during Mg plating and stripping (Figure 14f).

The formation of electroactive Mg_x_Cl_y_^z+^ cations in Mg(HMDS)_2_/AlCl_3_‐based and related electrolyte formulations has also been explored,^[^ ^38^ ^]^ highlighting the impact of composition on electrochemical performance. In the Mg(HMDS)_2_‐(AlCl_3_)_2_‐TEGDME electrolyte, the Coulombic efficiency for Mg plating/stripping was initially low. However, adjusting the Mg:Al molar ratio and introducing an equivalent amount of MgCl_2_ to the MgCl_2_/AlCl_3_/G4 electrolyte prevented the formation of a cationic Al species, which otherwise contributed to performance degradation. As proof, the Mg(HMDS)_2_/(AlCl_3_)_2_/MgCl_2_/G4 electrolyte exhibited “conditioning‐free” Mg plating/stripping. Furthermore, optimizing the stoichiometry of Mg and Al species can mitigate Al depletion and Mg corrosion, which are common challenges in Al‐containing electrolytes. Additionally, Xie et al. introduced phenyl disulfide (PD) as a film‐forming additive in the Mg(HMDS)_2_/MgCl_2_/THF electrolyte to regulate Mg plating/stripping.^[^ ^247^ ^]^ During electrochemical cycling, PD molecules dissociate into phenyl thiolate anions, which contribute to forming an organic interfacial layer on Mg anodes, effectively preventing excessive passivation layer growth and enhancing reversibility.

Mg(HMDS)_2_‐based chlorine‐containing electrolytes have demonstrated the ability to enable reversible Mg plating/stripping with high Coulombic efficiencies and ionic conductivities, making them suitable for Mg batteries. Building on this, researchers have extended these electrolytes to bromine‐containing systems. For instance, Dongmo et al. developed MgHMDSBr‐based electrolyte (Mg(HMDS)Br, Mg(HMDS)Br–BEt_3_, and Mg(HMDS)Br–AlEt_3_ dissolved in the THF).^[^ ^248^ ^]^ These bromine‐containing electrolytes maintained a comparable electrochemical stability window while exhibiting high ionic conductivity (≈1.2 mS cm^−1^), a relatively high anodic stability of 2.4 V vs Mg^2+^/Mg, excellent long‐term cycling stability (1000 cycles), and extremely efficient Mg plating/stripping (99%).

#### 4.4.3 Boron‐Containing Salt‐Based Electrolytes

Boron salt‐based Mg battery (molecular modified boron) electrolytes incorporate a range of boron‐containing anions, including tetrafluoroborate (BF_4_^−^), tetracyanoborate (B(CN)_4_^−^), and polyhedral borates such as closo–dodecaborate ((B_12_H_12_)^2−^) and its functionalized derivatives. The large, delocalized charge distributions and weak coordinating ability of boron‐based anions effectively reduce ion‐pair formation with cations (e.g., Li^+^, Na^+^, Mg^2+^), leading to increased ionic conductivity and improved cation transference numbers. Furthermore, the structural rigidity and high thermal stability of polyhedral borates contribute to enhanced thermal and electrochemical stability of the electrolyte. Closo–borates, in particular, possess exceptional oxidative stability (up to 5 V vs Li^+^/Li), making them suitable for high‐voltage applications. In addition, boron‐based anions often facilitate the formation of robust and uniform SEIs on electrode surfaces. This stabilizes electrode–electrolyte interfaces, mitigates side reactions, and extends cycling life of batteries.

Although boron salt‐based Mg battery electrolytes were first discovered in 1957,^[^ ^249^, ^250^, ^251^ ^]^ their development was slow due to challenges such as the co‐deposition of boron and magnesium. Recently, they have gained attention for their excellent air stability, compatibility with various substrates, high corrosion resistance, and strong reversibility in Mg plating/stripping.^[^ ^252^, ^253^, ^254^ ^]^ Despite these advantages, boron‐based salts generally exhibit low solubility in ether solvents like THF and suffer from poor oxidation stability due to the presence of alkyl groups, which are prone to β‐H elimination reactions.^[^ ^253^, ^255^ ^]^ Among them, Mg(BH_4_)_2_ has emerged as a promising high‐performance electrolyte when dissolved in ether–like solvents.^[^ ^256^, ^257^ ^]^ However, its inherently poor oxidation stability limits its compatibility with high‐voltage cathode materials, posing a challenge for its broader application in Mg batteries.

**Figure 15:**
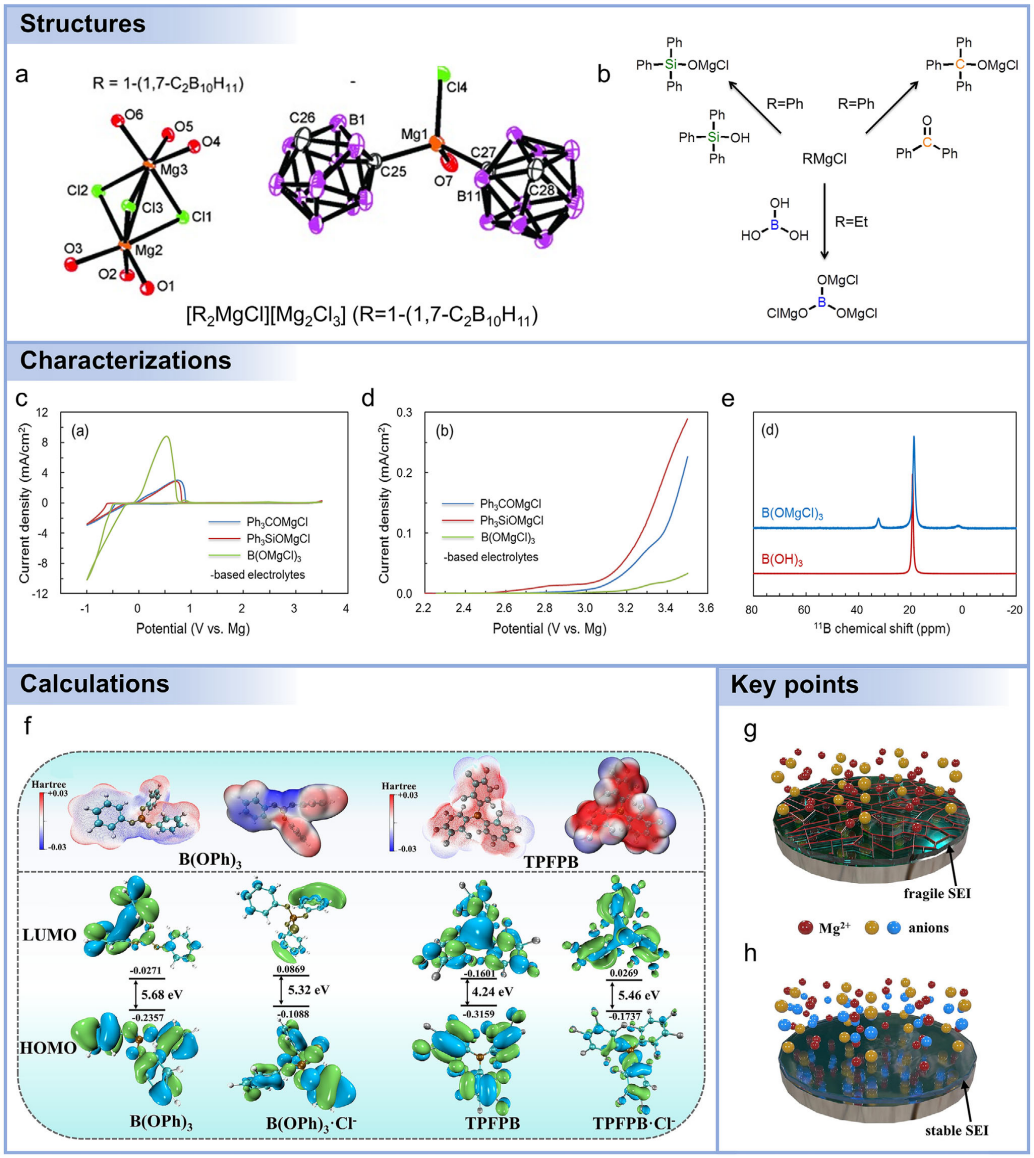
a) X‐ray crystal structure of [R_2_MgCl][Mg_2_Cl_3_] (R = 1‐(1,7‐C_2_B_10_H_11_) Reproduced with permission.^[^ ^217^ ^]^ Copyright 2014, WileyVCH. b) Schematic synthetic routes of various Mg salts. c) CV curves of Ph_3_COMgCl– (blue), Ph_3_SiOMgCl– (red), and B(OMgCl)_3_‐based (green) electrolytes during the 100th cycle. Measurements were taken at a scan rate of 5 mV s^−1^ at 20°C using Pt, Mg, and Mg foils as the working, reference, and counter electrodes, respectively, in a three‐electrode cell. d) LSV curves of Ph_3_COMgCl– (blue), Ph_3_SiOMgCl– (red), and B(OMgCl)_3_‐based (green) at a scan rate of 5 mV s^−1^ at 20 °C. (e) ^11^B NMR spectra of B(OMgCl)_3_ (blue) and B(OH)_3_ (red) dissolved in CD_3_OD/CD_3_CO_2_D (50/50 vol%). Reproduced with permission.^[^ ^258^ ^]^ Copyright 2020, Springer Nature. f) Visualization of the DFT calculations. ESP iso‐surface of B(OPh)_3_ and TPFPB with different degrees. The negative charge is represented in blue and the positive is represented in red. Visualized LUMO/HOMO orbitals and transition gap energy of B(OPh)_3_ and TPFPB molecules; [B(OPh)_3_Cl]^−^ and [TPFPBCl]^−^ anions. Schematic illustration of proposed effect and morphology modulation by TPFPB modification, g) without TPFPB, h) with TPFPB. Reproduced with permission.^[^ ^259^ ^]^ Copyright 2023, Elsevier.

When Mg(BH_4_)_2_ is used as a hydrogen storage material, it often leads to the byproduct formation, namely magnesium dodecahydro‐closo‐dodecaborate (MgB_12_H_12_). Inspired by this observation, Carter et al. explored carbon borane‐based magnesium compounds as potential electrolyte salts for Mg batteries.^[^ ^217^ ^]^ As illustrated in **Figure** **15**a, these electrolytes exhibited an ionic conductivity of 0.6 mS cm^−1^, oxidation stability exceeding 3 V vs Mg^2+^/Mg, and a Coulombic efficiency of 98.2%. Regrettably, despite these promising properties, their low solubility in conventional ether solvents rendered them unsuitable for Mg battery applications, highlighting a critical challenge in the development of boron‐containing salts.

As an example, Mes_3_B–(PhMgCl)_2_/THF electrolytes have received extensive attention due to their straightforward preparation by reacting tris(3,5‐dimethylphenyl)borane (Mes_3_B) with PhMgCl in THF.^[^ ^252^ ^]^ This reaction leads to the formation of various Mg species, including Mg_2_Cl_3_^+^, MgCl^+^, Ph_2_Mg, and the tetrahedral boron anion (Mes_3_BPh)^−^. This electrolyte demonstrates a wide operating voltage window (3.5 V vs Mg^2+^/Mg) and achieves a high cycling efficiency (100%) for Mg plating/stripping. Fluorescence and Raman spectroscopy analyses indicated that the remarkable stability was attributed to non‐covalent interactions between (Mes_3_BPh)^−^ and MgPh_2_.^[^ ^206^ ^]^ However, the electrode remains vulnerable to corrosion caused by chlorine, presenting a challenge for long‐term applications.

Subsequently, a boron–centered anion‐containing electrolyte (BCM) was synthesized by reacting tri(heptafluoroisopropyl)borate [B(OC_3_HF_6_)_3_] with MgF_2_ in DME,^[^ ^260^ ^]^ offering a simple synthesis process, high ionic conductivity (1.1 mS cm^−1^), and a wide potential window (3.5 V vs Mg^2+^/Mg). The electrolyte primarily consists of [BF(OC_3_HF_6_)_3_]^−^ and [Mg(DME)_n_]^2+^, with new [B(OC_3_HF_6_)_4_]^−^ borate anions forming after multiple cycles. Building on this development, a novel boron‐based electrolyte (OMBB) was formulated by reacting tri(heptafluoroisopropyl)borate [B(OC_3_HF_6_)_3_], MgCl_2_, and Mg powder in DME, yielding [B(OC_3_HF_6_)_4_]^−^ and [Mg_4_Cl_6_(DME)_6_]^2+^. This electrolyte exhibits a high oxidation stability (3.3 V vs Mg^2+^/Mg), a high ionic conductivity (5.6 mS cm^−1^), low plating/stripping overpotentials (0.11 V), and a relatively high Coulombic efficiency (> 98%).

Following research led to the development of an efficient boron‐based electrolyte through a simple one‐step reaction involving MgCl_2_, triphenyl borate [B(OPh)_3_], and tris(pentafluorophenyl)boron (TPFPB) in THF.^[^ ^259^ ^]^ The strong electron‐withdrawing effect of TPFPB facilitates its preferential reaction with MgCl_2_ due to the substitution of –H by –F on the benzene ring, resulting in the formation of the active cation (MgCl)^+^ and the tetra‐coordinated boron‐based anion (TPFPBCl)^−^. Additionally, the remaining MgCl_2_ reacts with B(OPh)_3_, generating [B(OPh)_3_Cl]^−^. This electrolyte exhibits a wide electrochemical window (>3 V vs Mg^2+^/Mg), a low polarization voltage (150 mV), and an average Coulombic efficiency above 98%. As presented in Figure 15f, charge distribution analysis reveals that negative charges accumulate primarily on the terminal benzene ring of B(OPh)_3_, while fluorine substitution enables a more uniform distribution of negative charges. Furthermore, TPFPB exhibits a lower HOMO level, enhancing oxidative stability and making this electrolyte a promising candidate for high‐performance Mg batteries. Since [TPFPBCl]^−^ possesses a higher transition gap energy and a lower HOMO than the [B(OPh)_3_Cl]^−^ anion, its presence in the electrolyte likely enhances oxidative stability. In contrast, the absence of TPFPB leads to the formation of an unstable SEI due to side reactions and electrolyte decomposition (Figure 15g). As a strong Lewis acid, TPFPB can react with the Mg anode to produce MgF_2_ when included in the electrolyte (Figure 15h). The resulting stable and electrically insulating MgF_2_–rich SEI layer effectively prevents electrolyte decomposition and extends battery cycle life. Additionally, the high ionic conductivity remains unaffected, ensuring the fast diffusion of Mg^2+^ and maintaining optimal electrochemical performance.

In related work, Ph_3_COMgCl–, Ph_3_SiOMgCl–, and B(OMgCl)_3_‐based electrolytes were developed in G3.^[^ ^258^ ^]^ These Mg salts were synthesized by reacting benzophenone, triphenylsilanol, and boric acid, respectively, with a Grignard reagent, followed by the addition of AlCl_3_, stirring, and cooling to room temperature to obtain the desired electrolytes (Figure 15b). CV results revealed reversible cathodic and anodic currents, with the B(OMgCl)_3_‐based electrolyte displaying the highest reversible current density for Mg plating/stripping (Figure 15c). The Coulombic efficiencies were 75.7, 81.5, and 64.0% for Ph_3_COMgCl–, Ph_3_SiOMgCl–, and B(OMgCl)_3_‐based electrolytes, respectively. Linear sweep voltammograms (LSV) curves (Figure 15d) indicated that the Ph_3_COMgCl‐based electrolyte exhibited anodic stability close to 3.0 V vs Mg^2+^/Mg, while the Ph_3_SiOMgCl‐based electrolyte showed a minor oxidation current at 2.6 V vs Mg^2+^/Mg. The B(OMgCl)_3_‐based electrolyte displayed an anodic current near 3.0 V vs Mg^2^⁺/Mg at the electrolyte decomposition potential, demonstrating that anodic stability is influenced by the central element. Although Mg plating/stripping with high anodic stability was observed in the B(OMgCl)_3_‐based electrolyte, the Coulombic efficiency was only ≈60% at the 100th cycle. The presence of BO_3_^3−^ was evidenced by ^11^B NMR spectra of B(OH)_3_ and B(OMgCl)_3_ (Figure 15e). The predominant species in this system included [Mg_2_(µ‐Cl)_2_]^2+^, [Mg_2_(µ‐Cl)_3_]^+^, glyme‐solvated Mg^2+^, and AlCl_4_^−^. Notably, since the B(OMgCl)_3_‐based electrolytes lacked AlCl_2_^+^, they were expected to be non‐corrosive. Additionally, a novel boron‐based and non‐nucleophilic electrolyte was synthesized by reacting tris(2,2,2‐trifluoroethyl)borate [B(TFE)_3_] with MgCl_2_, CrCl_3_, and Mg powders in DME.^[^ ^261^ ^]^ The active species in this electrolyte were identified as [B(TFE)_4_]^2−^ and [Mg_2_(µ‐Cl)_2_(DME)_4_]^2+^, where two Cl atoms bridged the Mg centers, and four oxygen atoms from two DME molecules occupied the remaining coordination sites. The [B(TFE)_4_]^2−^ anion consists of a B center coordinated to four TFE groups. Although this electrolyte shows a slightly reduced overpotential to 140 mV at 0.1 mA cm^−2^ for Mg plating/stripping and a slightly improved Coulombic efficiency (≈97%), the anodic stability can be largely extended to approx≈3.5 V vs Mg^2+^/Mg.

In conclusion, the combination of boron‐based salts with chlorine‐containing compounds enables the construction of electrolytes with considerable electrochemical performance. The introduction of Cl^−^ sources, such as AlCl_3_ or MgCl_2_, into boron‐based electrolyte systems induces structural reorganization of the boron‐centered anionic framework, facilitating more efficient Mg^2+^ transport. The presence of additional Cl^−^ anions also promotes the formation of a stable and uniform interphase on Mg anodes, effectively lowering the overpotential for Mg plating/stripping processes. This synergistic effect between boron‐centered anions and chloride species results in improved ionic conductivity, extended electrochemical stability windows, and enhanced interfacial compatibility.

## 5 Advancement in Chlorine‐Free Electrolytes

Electrolytes containing chloride often induce corrosion, prompting recent efforts to develop chlorine‐free alternatives that enable reversible magnesium deposition without forming a passivation layer. These electrolytes typically include MgX_2_, salts, where X = PF_6_^−^, BH_4_^−^, TFSI^−^, and OTf^−^, along with large and weakly coordinating anions.^[^ ^41^ ^]^ Despite the achieved advancements, there are still several critical challenges to address, particularly in terms of ionic conductivity, electrochemical stability, and compatibility with electrode materials. To overcome these obstacles, future research should prioritize optimizing the performance of chlorine‐free electrolytes, aiming to achieve a balance between efficiency, safety, and environmental sustainability. **Table** **3** summarizes the properties of reported chlorine‐free Mg battery electrolytes.

**Table 3:** The properties of Mg battery chlorine‐free electrolytes.

Classification	Electrolyte^a^ ^–^ ^f^ ^)^	IC [mS cm^−1^]	ESW [V vs Mg^2+^/Mg] (CE)			Refs.
			Pt	SS	Au	
Mg(TFSI)_2_‐based chlorine‐free electrolytes	0.3 M Mg(TFSI)_2_/DME+G2	3.03	–	4.0	–	[26]
	0.1 M Mg(TFSI)_2_/G2+DMAPA	1.02	–	3.7	–	[262]
	0.5 M Mg(TFSI)_2_/DME+M1	4.7	–	(≈20%)	–	[185]
	0.5 M Mg(TFSI)_2_/DME+M2	4.4	–	(≈50%)	–	[185]
	0.5 M Mg(TFSI)_2_/DME+M3	5.3	–	(≈99%)	–	[185]
	0.5 M Mg(TFSI)_2_/DME+M4	4.0	–	3.8 (99.5%)	–	[185]
	0.5 M Mg(TFSI)_2_/DME+TMP	–	–	4.0	–	[263]
	Mg(BH_4_)_2_/MPEG_7_PyrTFSI	–	–	≈2.5 (90%)	–	[264]
	0.4 M Mg(TFSI)_2_–4.0 M IBA/G2	–	–	3.92 (90%)	–	[265]
	0.4 M Mg(TFSI)_2_/DME	1.7	–	–	–	[266]
	0.4 M Mg(TFSI)_2_/DME+TEP	2.8	–	–	–	[266]
	0.4 M Mg(TFSI)_2_/DME+G2+BTFE+TEP	6.4	–	–	–	[266]
Mg(HMDS)_2_‐based chlorine‐free electrolytes	0.2 M Mg(HMDS)_2_–20 mM TBABH_4_/DME	0.32	1.98	3.18	–	[267]
	0.125 M Mg(OTf)_2_–0.125 M Mg(HMDS)_2_–0.5 M TBAOTf/DME+G3	–	4.43	3.63	–	[268]
Boron‐based chlorine‐free electrolytes	0.5 M Mg(BH_4_)_2_/THF	–	≈1.7 (40%)	–	≈2.2 (40%)	[256]
	0.5 M Mg(BH_4_)_2_/DME	–	≈1.7 (67%)	–	≈2.2 (67%)	[256]
	0.01 M Mg(BH_4_)_2_/G2	–	≈1.9 (77%)	–	–	[250]
	0.01 M Mg(BH_4_)_2_/THF	–	≈1.9 (34%)	–	–	[250]
	0.01 M Mg(BH_4_)_2_/DME	–	≈1.9 (67%)	–	–	[250]
	0.18 M Mg(BH_4_)_2_–0.6 M LiBH_4_/G2	–	≈1.7 (94%)	–	≈2.2	[256]
	0.1 M Mg(BH_4_)_2_–1.5 M LiBH_4_/G2	3.27	≈1.9 (100%)	–	–	[250]
	0.1 M Mg(BH_4_)_2_–0.6 M LiBH_4_/THF	2.61	≈1.9 (92%)	–	–	[250]
	0.1 M Mg(BH_4_)_2_–1.5 M LiBH_4_/DME	2.07	≈1.9 (85%)	–	–	[250]
	0.5 M Mg(BH_4_)_2_–1.5 M LiBH_4_/G4	9.66	2.0	–	–	[253]
	0.35 M Mg(BBu_2_Ph_2_)_2_/THF+DME	1 × 10^−3^	–	–	–	[211]
	0.25 M MgMe_3_BPh_2_/THF	1.5	≈2.6	–	–	[254]
	0.25 M Bu_2_Mg–BPh_3_/THF	–	–	–	1.20‐1.75 (71%‐93%)	[269]
	0.75M Mg(CB_11_H_12_)_2_/G3	2.9	3.4 (79.6%)	–	–	[42]
	0.75M Mg(CB_11_H_12_)_2_/G4	1.8	3.8 (94.4%)	–	–	[42]
	0.5 M Mg(BH_4_)_2_‐1.0 M THFPB/G2	3.72	–	2.8	–	[270]
	0.05 M Mg(BArF)_2_/THF	1.5	–	4.0	–	[255]
	0.5 M Mg(CB_11_H_12_)_2_/DME+G2	6.1	–	–	–	[148]
	0.6 M MgBOR(hfip)/DME	6.8	3.5 (98%)	4.3	–	[271]
	0.3 M Mg[B(HFIP)_4_]_2_/DME	≈11	4.5	≈4.0	–	[188]
	0.5 M Mg[B(O_2_C_2_(CF_3_)_4_)_2_]_2_/G2	3.95	–	≈4.0 (81%)	–	[272]
	0.3 M Mg[B(HFIP)_4_]_2_/G3	3.3	–	–	–	[273]
	0.75M Mg[B(Otfe)_4_]_2_/THF	0.401	–	3	–	[274]
	0.25M MBA–B(Otfe)_3_/THF	–	≈1.8	2.8	–	[274]
	MgF_2_–B(Otfe)_3_/THF	–	≈3.0	≈2.5 (80%)	–	[275]
	0.05 M MgF_2_–0.5 M THFPB/DME	1.1	–	3.5	–	[260]

^a)^ M1: diaza‐18‐crown‐6;

^b)^ M2: bis(2‐methoxyethyl)amine;

^c)^ M3: 2‐methoxyethylamine;

^d)^ MPEG_7_PyrTFSI: methoxypolyethylene glycol (MW 350) bis(trifluoromethylsulfonyl)imide;

^e)^ TEP: triethyl phosphate;

^f)^ BTFE: bis(2,2,2‐trifluoroethyl) ether.

### 5.1 Mg(TFSI)_2_‐Based Electrolytes

TFSI^−^ has long been a reference anion due to its dispersed negative charge, providing exceptional structural and electrochemical stability.^[^ ^276^ ^]^ Around 2010, Mg(TFSI)_2_ gained considerable attention as a straightforward electrolyte salt due to its high solubility in various solvents, excellent ionic conductivity, and strong oxidation resistance.^[^ ^277^ ^]^ When dissolved in DME or G2, Mg(TFSI)_2_ enables reversible Mg plating/stripping and demonstrates excellent anodic stability on Cu, stainless steel (SS), and Al collectors.^[^ ^26^ ^]^ Electrolytes based solely on Mg(TFSI)_2_ are incompatible with Mg metal because of surface passivation caused by the decomposition products, which severely hinders reversible Mg plating/stripping.

**Figure 16:**
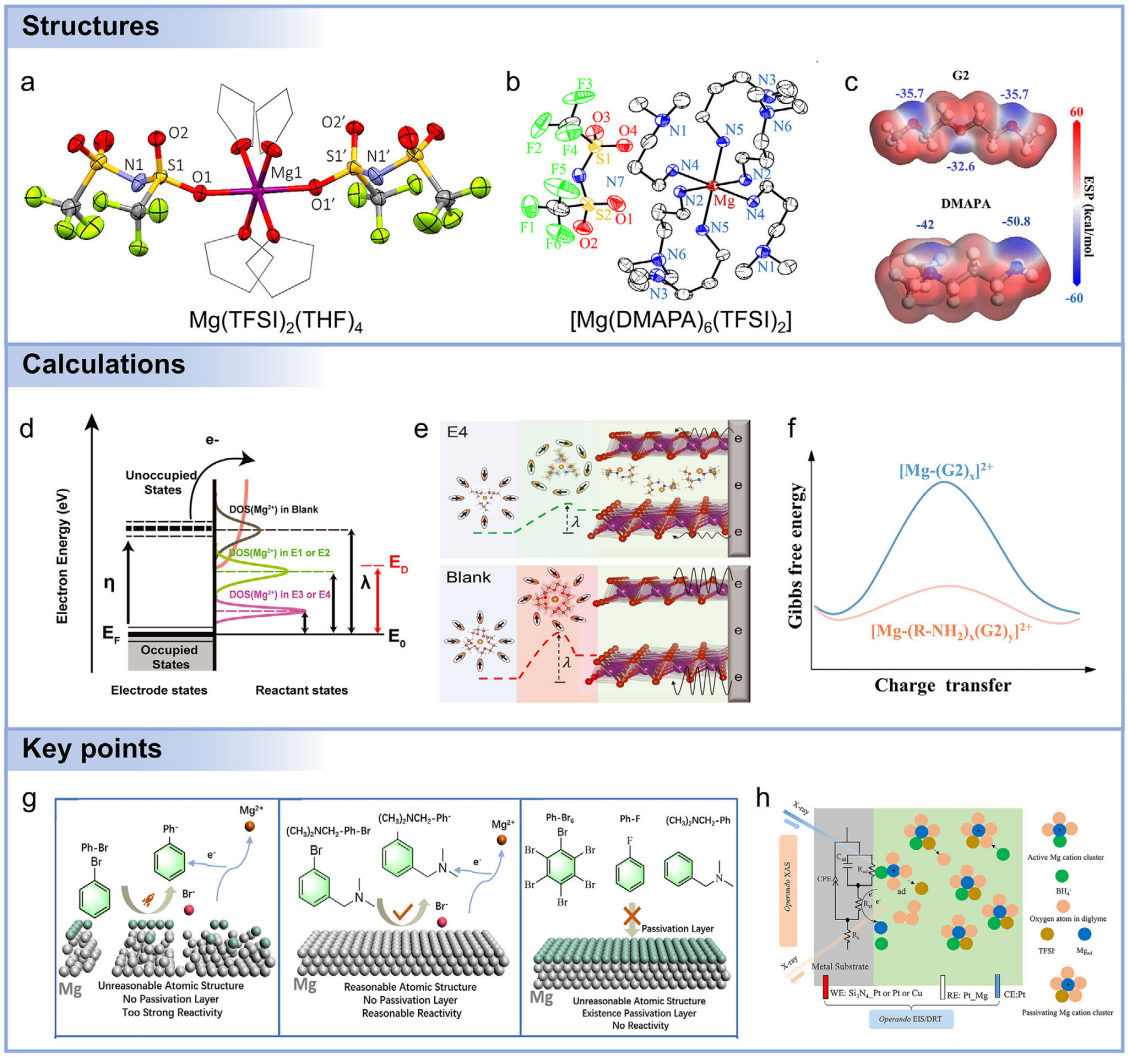
a) Single crystal structure of the Mg(TFSI)_2_(THF)_4_ electrolyte. Displacement ellipsoid representation (50% probability level). For clarity, four solvated THF molecules are in wireframe style. Selected bond distances (Å): Mg1–O1, 2.074 Å; S1–O1, 1.448 Å; S1–O2, 1.422 Å. Reproduced with permission.^[^ ^278^ ^]^ Copyright 2020, American Chemical Society. b) The ORTEP of the single‐crystal [Mg(DMAPA)_6_(TFSI)_2_] structure (50% thermal probability ellipsoid). The hydrogen atom and the disordered second component are omitted for the sake of clarity. c) The ESP distribution in G2 and DMAPA solvents. Reproduced with permission.^[^ ^262^ ^]^ Copyright 2024, The Royal Society of Chemistry. d) The Marcus–Gerischer diagram of electron transfer at the metal electrode–electrolyte interface. e) The scheme of concerted ion and electron transfer in the cathode host is limited by the solvation sheath reorganization. Reproduced with permission.^[^ ^185^ ^]^ Copyright 2021, The Authors, published by AAAS. f) Mechanistic comprehension of the amine solvent's role in traditional Mg(TFSI)_2_/ether electrolytes. Reproduced with permission.^[^ ^262^ ^]^ Copyright 2024, The Royal Society of Chemistry. Molecular structure design of component modifiers for rechargeable Mg batteries in Mg(TFSI)_2_/ether electrolyte. g) Molecular structure design of component modifiers for rechargeable Mg batteries in Mg(TFSI)_2_/ether electrolyte. Schematic illustrations: (left) serious intergranular corrosion fracture of Mg‐metal anodes due to violent reactions with highly reactive component modifiers, such as bromobenzene, p‐dibromobenzene, and 2‐bromoethylbenzene; (middle) cleaning passivation layer on Mg‐metal anodes with optimal BPDMA component modifier; (right) passivation layer formation on Mg‐metal anodes with nonreactive component modifiers, such as hexabromobenzene, fluorobenzene and N,N‐dimethylbenzylamine. Reproduced with permission.^[^ ^279^ ^]^ Copyright 2024, WileyVCH. h) Schematic illustration of probing the electrified interface involving adsorption of active Mg^2+^ clusters. Reproduced with permission.^[^ ^280^ ^]^ Copyright 2020, American Chemical Society.

Several studies have explored the effects of solvent systems on Mg(TFSI)_2_‐based electrolytes. For instance, 0.3 M Mg(TFSI)_2_/DME+G2 electrolytes support reversible Mg plating/stripping on Cu collectors but they exhibit high potential polarization and unstable potential profiles.^[^ ^26^ ^]^ Persson et al. employed first‐principles calculations and MD simulations across various electrolytes, identifying a unique phenomenon related to concentration‐dependent ion pair formation and anion reactivity.^[^ ^23^ ^]^ Mg salts tend to form ion pairs and undergo partial reduction (Mg^2+^ → Mg^+^), which activates TFSI^−^ and makes it susceptible to decomposition. In 2019, Sa's group proposed introducing a nitrogen‐containing chelating agent to enhance electrolyte performance.^[^ ^278^ ^]^ Dimethylamine (DMA), when added as a cosolvent, facilitated Mg(TFSI)_2_ dissolution in THF, enabling reversible Mg plating/stripping with a Coulombic efficiency of up to 90%. Structural analyses, including single‐crystal XRD, PDF, and NMR, reveal that Mg^2+^ coordinate with six oxygens, two from TFSI^−^ and four from THF, while the nitrogen of DMA is present in the first solvation shell (**Figure** **16**a). These findings contrast with earlier studies on magnesium electrolytes containing halogens, which consistently identified Mg cations in the form of [Mg_2_(µ‐Cl)_3_(THF)_6_]^+^ dimers as the primary active species.

Traditional perspectives on direct solvation sometimes fall short in explaining the implicit relationships among solvent, active ionic species, and electrode–electrolyte interfaces. To control electrochemically active species and minimize adverse interactions with the Mg anode, Cui et al. demonstrated that ether/amine co‐solvents facilitated both direct coordination and partial ionization.^[^ ^262^ ^]^ Specifically, they developed the Mg(TFSI)_2_/(G2+DMAPA) (DMAPA = 3‐dimethylaminopropylamine) electrolyte. As shown in Figure 16b, this system forms a distinct single‐crystal structure of [Mg(alkylamine)_6_](TFSI)_2_. The superior coordination ability of amines is further supported by ESP distributions of the two solvents, shedding light on the coordination reactions occurring within the electrolyte (Figure 16c).

Co‐solvents or anionic additives are effective strategies for preventing ion pair formation and addressing interface passivation issues. Wang's group developed a versatile electrolyte by adding methoxyethyl‐amine chelants to Mg(TFSI)_2_‐based electrolytes with different solvents, significantly enhancing interfacial charge transfer kinetics and suppressing side reactions through solvation sheath restructuring.^[^ ^185^ ^]^ In an electrochemical process, the overpotential (η) is the potential needed to adjust the electrode's Fermi level (E_F_) for each electron transported, while the reorganization energy (λ) is the energy required to rearrange the solvation sheath to accommodate electron transfer (Figure 16d). The inclusion of 1‐methoxy‐2‐propylamine (M4) in the solvation sheath notably improved charge transfer kinetics, indicating that solvation sheath restructuring also occurs at the cathode, thereby limiting interfacial reaction kinetics (Figure 16e). Similarly, adding trimethyl phosphate (TMP) to the Mg(TFSI)_2_/DME electrolyte softens solvation sheath deformation and rearranges the coordination structure from [Mg(DME)_3_]^2+^ to [Mg(DME)_2_TMP]^2+^.^[^ ^263^ ^]^ These coordination interactions involve electron redistribution, where strong binding to DME results in a transfer of 0.25 e^−^ per DME molecule to Mg^2^⁺, deforming the DME structure. The decomposition of organophosphorus molecules on the Mg surface significantly enhances Mg^2+^ transport while increasing electrical resistance by three and one order of magnitude, respectively. As illustrated in Figure 16f, a low charge transfer barrier was achieved through reorganization with the newly‐formed amine solvated Mg^2+^, facilitated by using ether/amine co‐solvents or additives.^[^ ^262^ ^]^ Additionally, ionic liquids (ILs) have been incorporated into the Mg(TFSI)_2_‐based electrolytes to enhance Mg battery performance. Buttry et al. demonstrated that chelating ILs containing polyether chains on pyrrolidinium cations could displace TFSI^−^ from the Mg^2+^ coordination sphere, enabling efficient Mg plating/stripping with excellent Coulombic efficiencies in these IL media.^[^ ^264^ ^]^

Recent investigations have clarified the mechanisms by which co‐solvents and anionic additives enhance electrolyte performance. Li et al. introduced isobutylamine (IBA) into a standard Mg(TFSI)_2_/G2 electrolyte, facilitating formation of [Mg^2+^(IBA)_5_]^2+^ and protonated amine‐based cations [(IBA)H]⁺.^[^ ^265^ ^]^ The direct solvation of IBA with Mg^2+^ prevents the decomposition of G2 and TFSI^−^ anions by generating neutral [(IBAH⁺)(TFSI^−^)]° complexes. Additionally, this electrolyte promotes uniform and dendrite‐free Mg electrodeposits, forming a thin, non‐passivated interphase containing MgH_2_ on the Mg surface. Nazar et al. introduced a novel co‐ether phosphate electrolyte (CEPE) system to address ion pair dissociation challenges and enhance Mg nucleation and growth.^[^ ^266^ ^]^ Using Mg(TFSI)_2_ as the salt, they selected a family of methyl phosphates ((CH_3_)_3_(CH_2_)*_n_* PO_4_, *n* = 0–4) with high dielectric constants and DNs as electrolyte additives to disrupt [Mg^2+^–TFSI^−^] contact ion pairs (CIPs). This approach enabled dendrite‐free Mg plating/stripping for over 10 months at a practical areal capacity of 2 mAh cm^−^2. Moreover, Mg||polyaniline full cells operated at voltages up to 3.5 V for more than 400 cycles at a fast 2 C rate, demonstrating outstanding capacity retention.

Additionally, Cui et al. proposed a molecular structure design approach to identify bromophenyl complex‐based component modifiers, considering the effects of electron‐donating and electron‐withdrawing substituents on Br–C bond dissociation reactivity.^[^ ^279^ ^]^ These electrolytes undergo in situ activation through spontaneous reactions between bromophenyl complexes and Mg anodes, facilitated by the hard Mg^2+^ cations from Mg(TFSI)_2_. As illustrated in Figure 1, 16‐(3‐bromophenyl)‐N,N‐dimethylmethanamine (BPDMA) exhibits moderate corrosion and passivation reactivity toward Mg metal anodes, making it an optimal component additive. Consequently, the optimized MgGB electrolyte, comprising Mg(TFSI)_2_ salt and G2+BPDMA mixed solvent, demonstrates stable Mg plating/stripping reversibility for ≈250 days. Comprehensive analyses revealed the formation of unique electrochemically active Br‐containing ion pairs, such as [(Mg^2+^)_2_(TFSI^−^)Br^−^]^2+^ and [(Mg^2+^)_2_(TFSI^−^)(Br^−^)(G2)_2_]^2+^, which contribute to significantly thinner Br^−^‐containing and organic–inorganic mixed interphases on Mg anodes.

Co‐salts can also enhance the performance of Mg(TFSI)_2_‐based electrolytes. Liu et al. demonstrated that the coordination of BH_4_^−^ to Mg^2+^ is significantly stronger than that of TFSI^−^. This strong coordination facilitates highly reversible Mg plating/stripping with high Coulombic efficiency by neutralizing the initial solvation shell of Mg^2+^ clusters formed between Mg^2^⁺ and TFSI^−^, while also enhancing the reductive stability of free TFSI^−^.^[^ ^280^ ^]^ As shown in Figure 16h, DRT analyses from operando EIS, together with XAS, NMR, and theoretical calculations identified a significant adsorption phase between Mg atoms and active Mg cation clusters involving BH_4_^−^ as primary facilitators for reversible Mg plating/stripping.

### 5.2 Mg(HMDS)_2_‐Based Electrolytes

**Figure 17:**
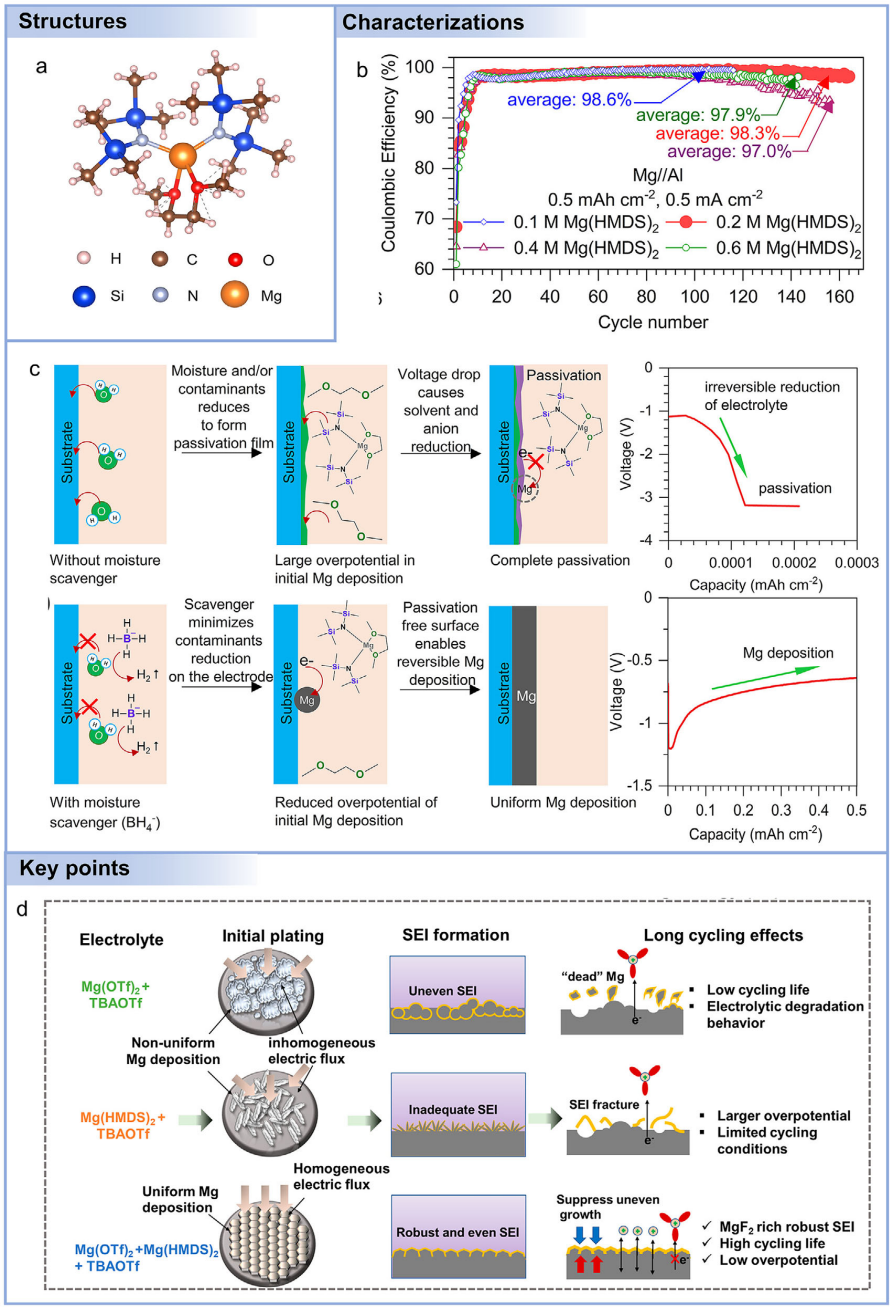
a) Molecular structure of Mg(HMDS)_2_(DME) complex. b) Coulombic efficiency of the Mg plating/stripping with varying amounts of Mg(HMDS)_2_. c) Schematic illustration of Mg deposition process without and with moisture scavenger in the electrolyte, respectively. The voltage limit for the plating process is set at −3.25 V vs Mg^2+^/Mg. Upon reaching this limit, the constant voltage was held at −3.25 V vs Mg^2+^/Mg until the end of plating. Reproduced with permission.^[^ ^267^ ^]^ Copyright 2021, American Chemical Society. d) Schematic representation of the formation of SEI in different electrolytes and related effects. Reproduced with permission.^[^ ^268^ ^]^ Copyright 2023, American Chemical Society.

The potential of Mg(HMDS)_2_‐based electrolytes to improve the electrochemical performance of Mg batteries has garnered significant interest. These electrolytes offer several advantages, including high ionic conductivity, compatibility with Mg metal anodes, and a broad electrochemical stability window. However, HMDS‐based electrolytes typically consist of Hauser base HMDSMMgCl and Lewis acid AlCl_3_, raising concerns related to chlorine‐containing systems. With increasing interest in chlorine‐free electrolytes, various electrolyte additives have been investigated. In 2021, Zhi et al. developed a chlorine‐free Mg(HMDS)_2_/DME electrolyte with tetrabutylammonium borohydride (TBABH_4_) as the additive, exhibiting noncorrosive to cell components.^[^ ^267^ ^]^ As shown in **Figure** **17**a, Mg in the Mg(HMDS)_2_(DME) structure exhibits a coordination number of four, associating with two HMDS^−^ anions and coordinating with a DME molecule through Mg–O bonds. By introducing TBABH_4_ to regulate moisture levels, this electrolyte achieved reversible Mg plating/stripping with an average Coulombic efficiency of 98.3% over 150 cycles (Figure 17b). The authors proposed that, in the absence of a moisture scavenger, the Mg anode reacts with residual moisture, leading to passivation and a significant voltage drop. In contrast, BH_4_^–^ effectively mitigates moisture, preventing passivation (Figure 17c). Additionally, surface and depth profiling analysis revealed the formation of robust SEIs at the anode–electrolyte interface, enabling stable Mg plating/stripping.

In 2023, the same research group added tetrabutylammonium trifluoromethanesulfonate (TBAOTf) into a mixed‐salt electrolyte comprising of Mg(HMDS)_2_ and magnesium triflate (Mg(OTf)_2_).^[^ ^268^ ^]^ This chlorine‐free electrolyte enhanced the solubility of the Mg salt and promoted the formation of MgF_2_‐rich SEIs during cycling. Consequently, it improved reversible Mg plating/stripping and achieved high anodic stability (4.43 V vs Mg^2+^/Mg). Notably, the electrolyte decomposition product (CF_3_–SO_3_), known for its Mg^2+^ diffusion resistance was found exclusively on the electrode surface. A schematic illustration of SEI formation and its impact on cycling performance is presented in Figure 17d. In a Mg(OTf)_2_+TBAOTf electrolyte, the anode suffered from uneven SEI formation, which led to partial “dead” Mg formation and unsatisfactory cycling performance. Meanwhile, while Mg(HMDS)_2_+TBAOTf system exhibited insufficient SEI formation, causing cracks and increased overpotential. However, the Mg(OTf)_2_+Mg(HMDS)_2_+TBAOTf electrolyte facilitated the development of uniform and robust SEIs, effectively suppressing uneven Mg growth and enabling highly reversible Mg plating/stripping. This optimized electrolyte demonstrated exceptional cycling performance, even under high current densities and large areal capacities.

### 5.3 Boron‐Containing Salt‐Based Electrolytes

The exploration of boron‐containing salt‐based chlorine‐free electrolytes dates back to 1957 when Mg electrodeposition was first observed in Mg(BH_4_)_2_/ether solutions.^[^ ^251^ ^]^ Mohtadi et al. later reported the first example of Mg deposition/stripping using a halide‐free inorganic salt in both THF and DME solvents, though with relatively low CEs (<40% for THF, <70% for DME).^[^ ^256^ ^]^ To enhance the electrochemical performance of Mg(BH_4_)_2_‐based electrolytes, tris(2H‐hexafluoroisopropyl)borate (THFPB) was introduced as a multifunctional additive in a 0.5 M Mg(BH_4_)_2_/THFPB+diglyme electrolyte.^[^ ^270^ ^]^ This formulation exhibited high ionic conductivity (3.7 mS cm^−1^ at 25 °C) and excellent Coulombic efficiency (>99%), attributed to the strong electron–acceptor properties of THFPB. In 2013, Muldoon et al. developed a chlorine‐free electrolyte based on the [B(Ph)_4_]^−^, demonstrating that magnesium organoborates were non‐corrosive at voltages above 4.0 V vs Mg, providing a crucial reference for future B‐based electrolytes and high‐voltage cathode development.^[^ ^255^ ^]^ Additionally, 10‐vertex closo‐carborane anions have been explored for Mg battery electrolytes, offering excellent electrochemical stability, reversible Mg deposition/stripping, and halide‐free, non‐nucleophilic characteristics.^[^ ^281^ ^]^

**Figure 18:**
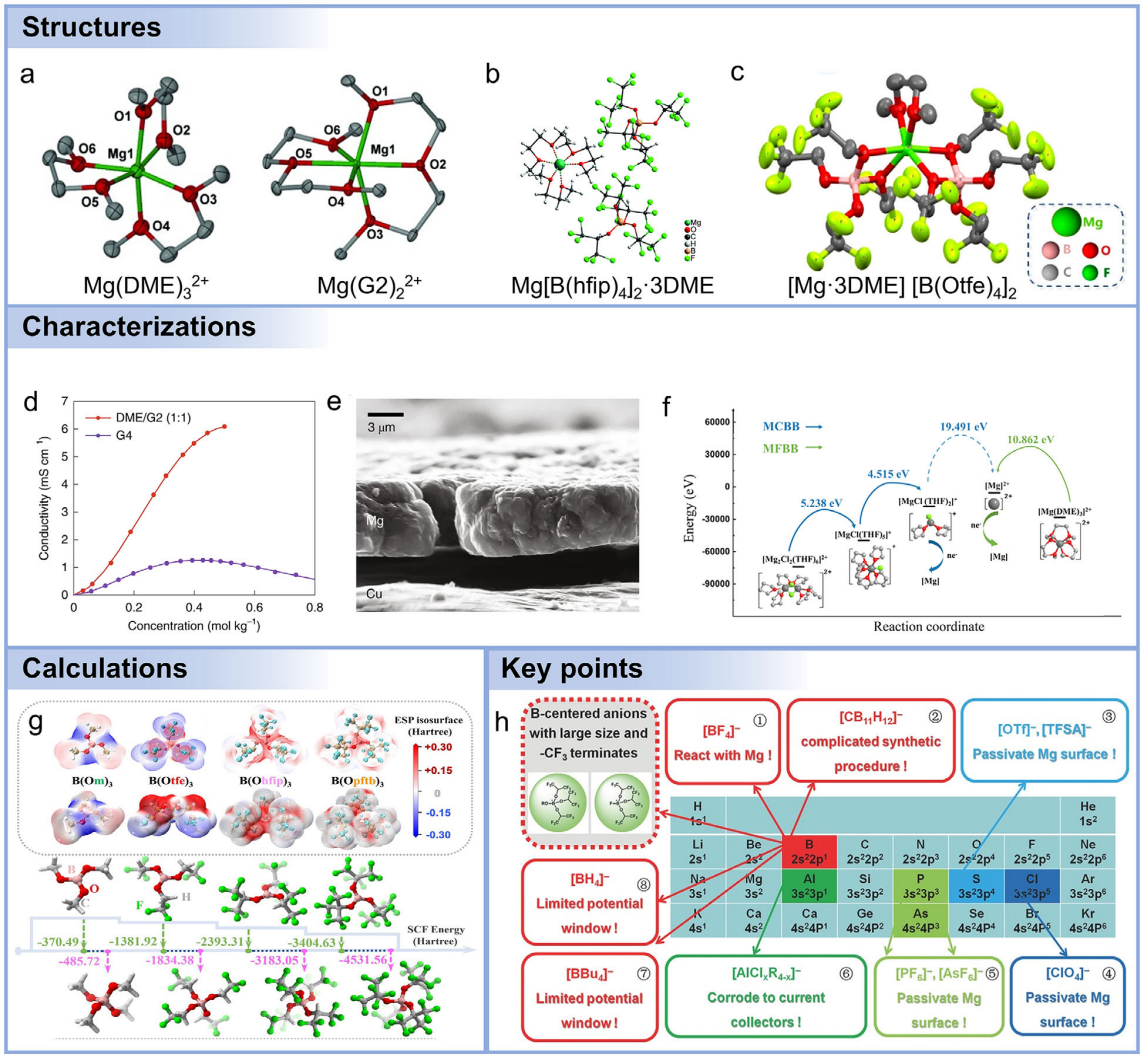
a) Mg cation coordination environment in [Mg(DME)_3_](CB_11_H_12_)_2_ and [Mg(G2)_2_](CB_11_H_12_)_2_(G2). Ellipsoids are set at 50 % probability; hydrogen atoms are omitted for clarity. Reproduced with permission.^[^ ^42^ ^]^ Copyright 2015, WileyVCH. b) Ball‐and‐stick representation of the Mg[B(hfip)_4_]_2_(DME)_3_ crystal. Reproduced with permission.^[^ ^271^ ^]^ Copyright 2017, The Royal Society of Chemistry. c) Metal complex structure of [Mg(DME)][B(Otfe)_4_]_2_ confirmed by single‐crystal XRD. Reproduced with permission.^[^ ^274^ ^]^ Copyright 2021, American Chemical Society. d) Ionic conductivity dependence on concentration of MMC solutions in G4 and DME/G2 mixture at 25 °C, e) cross‐sectional scanning electron microscopy image of Mg plated on a Cu substrate at 20 mA cm^−2^ with an areal capacity of 3 mAh cm^−2^. Reproduced with permission.^[^ ^148^ ^]^ Copyright 2020, The Authors, published by Springer Nature. f) Predicted scheme for the dissociation of divalent cationic groups on electrodes and reaction energy required for each step in the MFBB and MCBB electrolytes. Reproduced with permission.^[^ ^275^ ^]^ Copyright 2022, Elsevier. g) Visualization of the DFT theoretical calculation: electrostatic potential (ESP) iso‐surface of B(OR)_3_ borates with different degrees of −CF_3_ group substitution; the negative charge is represented in blue and the positive represented in red; SCF total energy distribution of structure‐optimized B(OR)_3_ borates and corresponding B(OR)_4_^–^ anions. Reproduced with permission.^[^ ^274^ ^]^ Copyright 2021, American Chemical Society. h) Decorated periodic table for directing efficient Mg battery electrolytes. Reproduced with permission.^[^ ^260^ ^]^ Copyright 2017, WileyVCH.

Boron clusters offer a promising approach to creating Mg battery electrolytes that meet stringent performance requirements. The bulky dianion cluster (B_12_H_12_)^2−^, which carries two highly delocalized negative charges, serves as a weakly coordinating anion for divalent Mg^2+^. Nevertheless, its poor solubility in ethers limits its electrochemical application. Accordingly, researchers have explored the substitution of B–H by C–H to enhance charge delocalization and reduce charge density. In 2015, Mohtadi et al. introduced the magnesium monocarborane salt Mg(CB_11_H_12_)_2_/tetraglyme (MMC/G4) electrolyte, forming solvent‐separated ion pairs (SSIPs), indicative of significant salt dissociation, a rare feature in simple Mg salts (**Figure** **18**a).^[^ ^42^ ^]^ This electrolyte demonstrated high ionic conductivity (1.8 mS cm^−1^), excellent Coulombic efficiency (>99 %), and anodic stability (3.8 V vs Mg^2+^/Mg).

Yao et al. investigated various solvent blends to enhance the solubility and ionic conductivity of MMC, achieving conductivities of 6.1 mS cm^−1^ in DME+G2 at 25 °C (Figure 18d).^[^ ^148^ ^]^ Remarkably, MMC/(DME+G2) allowed for Mg deposition/stripping with a CE of 99.7% even at 50 mA cm^−2^. Mg deposited on a Cu substrate at a rate of 20 mA cm^−2^ with an area capacity of 3 mAh cm^−2^ exhibited a smooth, dense, and dendrite‐free morphology (Figure 18e). Additionally, Mg||Mg symmetric cells demonstrate stable cycling, sustaining a cumulative capacity of 833 mAh cm^−2^, significantly surpassing previously reported values. The anodic stability of closo‐carborane salts is influenced by the electronic effects of carbon‐vertex substituent groups, with electron‐withdrawing groups (e.g., –CF_3_, –F, –NO_2_) enhancing anodic stability. Theoretical calculations showed that replacing hydrogen in C–H with strong electron‐withdrawing groups like –F or –CF_3_ increases the oxidation onset potential by ≈200 mV.^[^ ^282^ ^]^

Despite their excellent ionic conductivities and anodic stabilities, boron‐centered electrolytes with closo‐carborane cluster anions still require further improvements, particularly in simplifying synthesis methods. Among the most promising electrolytes for practical Mg batteries are magnesium fluorinated alkoxyl organoborates (Mg[B(OR^F^)_4_]_2_ (R^F^ can be OCH_2_CF_3_, OCH(CF_3_)_2_, etc.). Zhao‐Karger et al. made significant progress by in situ synthesizing Mg[B(ORF)_4_]_2_ through a single‐step dehydrogenation reaction between Mg(BH_4_)_2_ and fluorinated alkyl alcohols (H‐ORF).^[^ ^271^ ^]^ X‐ray crystallography confirmed the crystal structure of Mg[B(hfip)_4_]_2_(DME)_3_, as shown in Figure 18b. This electrolyte displayed high anodic stability (> 4.5 V vs Mg^2+^/Mg on Pt) and excellent ionic conductivity (≈11 mS cm^−1^).^[^ ^188^ ^]^ Building on this design, a Mg–FPB‐based electrolyte (FPB = B[O_2_C_2_(CF_3_)_4_]_2_) was developed, showing high anodic stability up to 4.0 V vs Mg^2+^/Mg on stainless steel.^[^ ^272^ ^]^ Alternatively, Mandai et al. proposed an ex situ synthesis method via a transmetalation reaction between Mg(OR^F^)_2_ and B(OR^F^)_3_.^[^ ^273^ ^]^ Although more complex, the Mg plating/stripping current density of Mg[B(Ohfip)_4_]_2_ synthesized ex situ was three times higher than that of the in situ method.

The search for new sources of magnesium and boron to develop effective electrolytes using straightforward synthesis techniques remains a key area of research. A novel electrolyte, Mg[B(Otfe)_4_]_2_ (Mg[B(OCH_2_CF_3_)_4_]_2_), was successfully synthesized by Nuli et al., showcasing the advantages of bulky B‐centered anions with low cost‐effectiveness and coordination.^[^ ^274^ ^]^ Single‐crystal XRD proved its metal complex structure (Figure 18c). This work systematically examined borates with non‐(B(Om)_3_) [B(OCH_3_)_3_], mono‐(B(Otfe)_3_), bis(B(Ohfip)_3_), and tri(B(Opftb)_3_) [B[OC(CF_3_)_3_]_3_] substitution of –CF_3_ and their subsequently formed anions using DFT calculations. As shown in Figure 18g, the electrostatic potential (ESP) iso‐surface for B(OR)_3_ borates shows positive (red) and negative (blue) charge distributions, with negative charge initially concentrated on the O atoms before fluorination. Substituting H with –CF_3_ shifts the negative charge to the electron‐accepting group, increases dipole moments, and decreases the self‐consistent field (SCF) total energy for each additional −CF_3_ substituent. Electrochemical performance studies on stainless steel demonstrated oxidation stability above 3 V vs Mg^2+^/Mg, low overpotentials of 0.2 V, and an average Coulombic efficiency of 99%. To further explore the benefits of magnesium chloride–complexed cationic clusters, Cheng et al. synthesized two non‐nucleophilic boron‐containing salts, MgF_2_–B(Otfe)_3_ (MFBB) and MgCl_2_–B(Otfe)_3_ (MCBB).^[^ ^275^ ^]^ DFT calculations revealed that cations containing chloride exhibited a lower desolvation energy barrier (Figure 18f). The unique electrochemical properties of these electrolytes were attributed to the active cationic species, further reinforcing the potential of boron‐based electrolytes in magnesium‐ion battery development.

Given the expensive synthesis procedures, Cui et al. examined the fundamental challenges of Mg battery electrolytes, focusing on the role of anions in the electrolyte systems based on organoboron and Mg salts (Figure 18h).^[^ ^260^ ^]^ Boron‐based chlorine‐free electrolytes have been deemed among the most promising solutions for Mg batteries due to their high ionic conductivity, broad electrochemical stability window, and compatibility with Mg anodes. These properties contributed to enhanced battery performance while minimizing corrosion. However, the synthesis of boron‐containing salt‐based electrolytes remains complex and costly. Ongoing research aims to optimize their composition and structure to fully unlock their potential for practical applications.

In summary, chlorine‐free electrolytes offer broad electrochemical stability windows and good compatibility with high‐voltage cathodes, but their performance is often limited by ion–pair formation and Mg surface passivation. Strategies such as introducing amine co‐solvents, additives, and ionic liquids have proven effective in restructuring the solvation sheath, suppressing decomposition, and facilitating uniform Mg plating/stripping. While significant progress has been made in improving ionic conductivity and interfacial stability, challenges related to synthesis complexity and long‐term cycling stability remain, underscoring the need for continued molecular level design of salts, solvents, and additives to realize practical chlorine‐free Mg batteries.

## 6 Trade‐Offs Between Chlorine‐Containing and Chlorine‐Free Electrolytes

In addition to these electrochemical considerations, practical implementation of Mg battery electrolytes must account for corrosivity, environmental impact, and cost. The chloride content in chlorine‐containing electrolytes can lead to significant corrosion of non‐noble metals and potential release of chlorine into the environment.^[^ ^255^, ^283^, ^284^, ^285^ ^]^ For instance, an APC electrolyte composed of 0.5 M PhMgCl and 0.25 M AlCl_3_ in THF contains ≈1.25 mol Cl per liter, corresponding to ≈0.50 g_Cl_ per 10 g of electrolyte. The Predicted Environmental Concentration (PEC) is estimated to reach ≈0.5 mg_Cl_ L^−1^ in a 1000 L water body, and could rise to ≈5 mg_Cl_ L^−1^ in a 100 L system. While these values remain below typical chronic toxicity thresholds for most freshwater organisms, they suggest that under conditions of low dilution or cumulative release, local ecological risks may still arise. By contrast, chlorine‐free electrolytes (e.g., Mg(TFSI)_2_, borate‐based salts) eliminate chlorine‐related corrosion and environmental concerns. Nevertheless, they introduce other elements (F, N, and S) with potential persistence, bioaccumulation, or toxicity issues that should be considered in a full environmental assessment. From an economic perspective, chlorine‐free systems are currently substantially more expensive than conventional chlorine‐containing electrolytes. Laboratory–scale prices indicate that APC electrolytes cost roughly 0.4 euro·mL^−1^, whereas 0.5 M Mg(TFSI)_2_ in DME is ≈36 euro·mL^−1^.^[^ ^134^ ^]^ While industrial‐scale production is expected to reduce these costs, the economic burden of highly purified fluorinated salts remains a significant consideration for large‐scale applications. Taken together, these points illustrate that, even as chlorine‐free electrolytes achieve impressive electrochemical performance, careful attention to material cost, environmental impact, and long‐term chemical stability is essential to guide practical Mg battery development.

**Figure 19:**
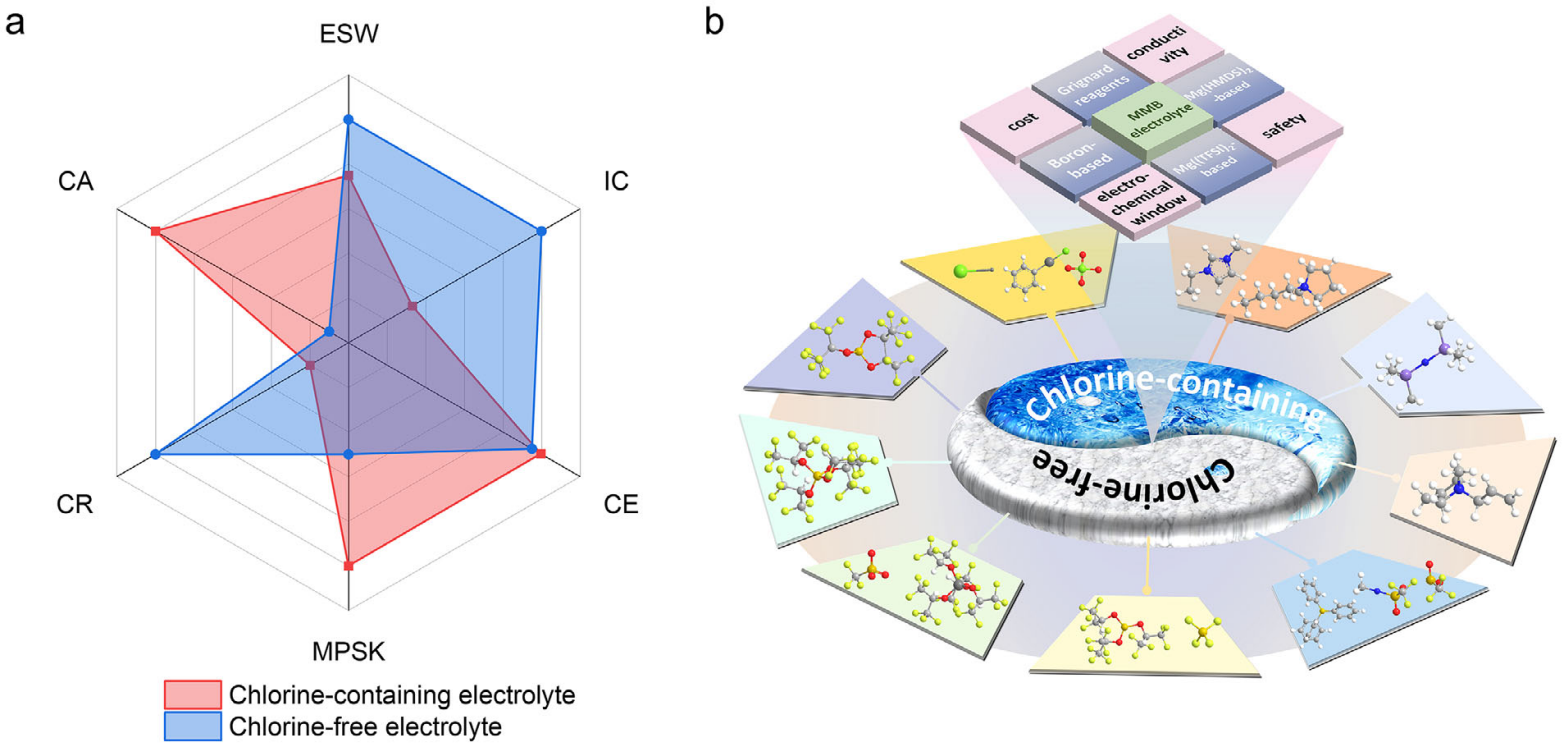
a) Radar comparison of key Mg electrolyte parameters (ESW, IC, CE, MPSK, CR, and CA). b) Schematic illustrations of atomic structures for chlorine‐containing and chlorine‐free Mg electrolytes.

Collectively, the radar chart (**Figure** **19**a) provides a visual comparison of representative chlorine‐containing and chlorine‐free Mg electrolytes across six key metrics: ESW, IC, CE, Mg plating/stripping kinetics (MPSK), corrosion resistance (CR), and cost advantage (CA). Normalized values allow direct comparison, with higher values representing more favorable performance, and reflect general trends rather than precise quantitative measurements. From this comparison, chlorine‐containing electrolytes generally exhibit excellent electrochemical performance (high ESW, IC, CE, and MPSA) and lower cost, but are limited by relatively low CR due to their chlorine content. In contrast, chlorine‐free electrolytes provide superior CR and probably avoid chlorine‐related environmental risks, but at the expense of higher cost and, in some cases, slightly lower interfacial performance. These observations highlight the trade‐offs between electrochemical performance, safety, and economic considerations, emphasizing that careful selection and molecular‐level optimization of electrolytes are essential for practical Mg battery development.

## 7 Conclusion and Perspectives

### 7.1 Conclusion

Mg batteries have emerged as a promising energy storage technology thanks to their potential for high performance, low cost, and enhanced safety. However, the divalent nature of Mg^2+^ leads to a large charge density and strong polarization, which impede further advancements in Mg batteries. One of the key challenges lies in developing practical electrolytes that are compatible with both Mg anodes and high‐performance cathodes. In this review, we provided a comprehensive analysis of the advances and challenges associated with both chlorine‐containing and chlorine‐free electrolytes for Mg batteries (Figure 19b). We examined the fundamental properties of Mg battery electrolytes, including their working mechanisms and selection principles for key components, including salts, solvents, and additives, while highlighting their respective advantages and limitations. Additionally, we assessed electrolyte performance by evaluating overpotential, Coulombic efficiency, and the reversibility of Mg plating/stripping processes.

A comparison of chlorine‐containing and chlorine‐free electrolytes highlights unique advantages and challenges for each. In particular, chlorine‐containing electrolytes, especially Mg–Al chloride complexes such as APC electrolyte, demonstrate superior ionic conductivity (≈10^−3^ S·cm^−1^) and highly reversible Mg plating/stripping with Coulombic efficiencies above 99%. In addition, the (MgCl_2_)_2_–AlCl_3_–Mg(TFSI)_2_/ether (G1 or G2) system, commonly referred to as MACT, has recently emerged as a highly promising electrolyte. MACT electrolyte exhibits high ionic conductivity (≈10^−3^ S·cm^−1^), Coulombic efficiencies above 98%, and improved anodic stability (up to ≈3.0 V vs Mg^2+^/Mg), while significantly reducing the corrosiveness typically associated with traditional chlorine‐based electrolytes. Chlorine‐containing species are more readily adsorbed onto electrode surfaces compared to organic ligands, mitigating Mg surface passivation and facilitating Mg dissolution at relatively low overpotentials (≈0.2 V). These properties have made chlorine‐containing electrolytes a favorable choice in the early stages of Mg battery research. However, their use raises significant safety and environmental concerns due to the highly reactive and corrosive nature. Notably, investigating additives or co‐solvents that stabilize the electrolyte and mitigate undesirable side reactions could significantly enhance both performance and safety.

While chlorine‐containing systems benefit from extensive research and established performance benchmarks, chlorine‐free electrolytes offer safer and more sustainable alternatives for Mg batteries. Among them, the Mg(TFSI)_2_/DME system has emerged as a promising platform, particularly when combined with functional additives. On its own, Mg(TFSI)_2_ in DME exhibits moderate ionic conductivity (≈10^−3^ S cm^−1^) but poor Mg plating/stripping reversibility due to surface passivation. However, with appropriate additives, such as Lewis acids and halogen‐containing species, its electrochemical performance can be significantly enhanced, achieving Coulombic efficiencies exceeding 99% and improved anodic stability up to 3.5–4.0 V vs Mg^2+^/Mg. On the other hand, the borate‐based electrolyte Mg[B(hfip)_4_]_2_/DME offers remarkable electrochemical stability (up to 4.3 V vs Mg^2+^/Mg), non‐corrosive nature, and high compatibility with high‐voltage cathodes. It enables reversible Mg cycling with efficiencies above 98%, even in the absence of chloride species. Due to its weakly coordinating anion and highly fluorinated structure, it minimizes undesired side reactions and ensures long‐term chemical stability. These electrolytes offer comparable ionic conductivity and electrochemical stability while avoiding the safety and environmental risks associated with chlorine. Furthermore, they tend to be less corrosive and more compatible with a broader range of electrode materials, making them a viable option for practical Mg batteries. Nevertheless, achieving stable and efficient Mg plating/stripping with chlorine‐free electrolytes remains a significant challenge.

The macroscopic differences between chlorine‐containing and chlorine‐free electrolytes can also be understood from the perspective of Mg^2+^ transport mechanisms and solvation structures. In chlorine‐containing electrolytes, the prevalent species (e.g., [Mg_2_(µ‐Cl)_3_(THF)_6_]^+^) are CIPs or large ionic aggregates.^[^ ^185^, ^195^, ^286^ ^]^ These bulky complexes rely mainly on vehicular motion, which restricts ion mobility and generally leads to low bulk ionic conductivity.^[^ ^14^, ^30^ ^]^ Nevertheless, chlorine‐containing electrolytes offer interfacial advantages that bridging Cl reduces the desolvation energy and facilitates Mg deposition/dissolution by weakening Mg–solvent interactions, while chloride adsorption at the Mg surface can modulate charge transfer kinetics and lower the deposition overpotential. By contrast, ion aggregation is effectively suppressed in chlorine‐free electrolytes, and the electrolytes generally consist of SSIPs, such as [Mg(DME)_3_]^2+^.^[^ ^14^, ^26^, ^30^, ^185^, ^195^, ^286^ ^]^ In this case, SSIP‐dominated species allow relatively small solvated entities to migrate in a relatively efficient way, thereby improving ionic conductivity. However, the strong bidentate coordination of DME to Mg^2+^ results in a high desolvation energy barrier. In addition, DME molecules can adsorb on the Mg surface, accelerating passivation and leading to significantly large overpotentials for Mg plating/stripping. This mechanistic comparison highlights the delicate trade‐off between bulk transport properties and interfacial kinetics, underscoring the importance of rationally tuning solvation and ion‐pairing behavior in future electrolyte design.

Looking ahead, investigating novel Mg salts, co‐solvent systems, and additives could yield significant improvements. Moreover, gaining a deeper understanding of electrolyte–electrode interface formation and systematically evaluating the effects of various additives on electrolyte performance will be crucial for advancing chlorine‐free Mg battery technology. Such progress will depend not only on empirical optimization, but also on integrating molecular‐level insights into solvation structures, ion transport mechanisms, and interfacial chemistry.

### 7.2 Perspectives

To advance Mg battery technology, there is a strong need to identify suitable electrolytes tailored to specific Mg battery systems through a multifaceted approach. In particular, efforts should be focused on the following aspects, including new component development, electrolyte concepts, fundamental understanding for design guidance, performance limitations, upscalability, and production.

1. Rationally designing electrolyte components. This includes exploring new salt formulations, solvents, additives, and their combinations. Achieving Mg plating/stripping efficiencies above 99% has proven challenging in previous studies involving amine‐, methoxy‐, or phosphonate‐based electrolytes. This limitation primarily stems from the formation of unstable intermediates and subsequent side reactions. Novel Mg salts with weakly coordinating anions or chelating agents can help improve the dissolution of Mg salts and reduce dendrite formation. Proper solvents with high electrochemical stability and low viscosity are required to optimize ionic conductivities. Developing new classes of additives that specifically target magnesium chemistry, such as SEI–inducing, chelation, and solvation–tuning additives, will be essential for improving the cycling performance and stability of Mg batteries.
2. Interfacial electric double layer engineering at the Mg anode. Elucidating the electric double layer structure at the Mg anode is vital for controlling interfacial processes. The inner Helmholtz layer, shaped by specific ion and solvent adsorption, directly modulates charge transfer and initial interphase formation. The outer Helmholtz layer reflects the dynamic solvation environment, governing ion accessibility and flux. Subtle variations in the composition and architecture of these layers critically influence nucleation behavior, deposition morphology, and interfacial stability. Strategic manipulation of double layer characteristics offers a pathway to engineer robust SEIs, enabling highly reversible Mg plating and stripping. A variety of analytical methods including synchrotron‐based characterizations and Sum Frequency Generation Infrared Spectroscopy are effective for revealing the structural characteristics of electrolytes and interfaces.
3. Emerging innovative electrolyte concepts. Solid–state electrolytes can potentially eliminate issues related to liquid electrolyte leakage, volatility, and dendrite formation, but render high interfacial resistance and slow Mg^2+^ mobility. In parallel, several new concepts such as high‐entropy, highly concentrated, sparingly solvating, and single‐ion conducting electrolytes, have shown considerable promise in Mg batteries. Furthermore, expanding the scope beyond traditional systems, magnesium‐based hybrid–ion batteries provide an alternative solution to the challenge of limited reversible cathode materials for Mg^2+^ storage.
4. Metrics toward practical Mg batteries. Conventional evaluation metrics, such as ionic conductivity (>1 mS cm^−1^), electrochemical stability (>4 V vs Mg^2+^/Mg), and Coulombic efficiency (>99%), provide essential benchmarks. Beyond these basic parameters, other often overlooked factors significantly affect the overall performance of Mg batteries. Low temperatures can increase viscosity and reduce ionic mobility, severely affecting charge transfer kinetics. Conversely, elevated temperatures can accelerate electrolyte degradation, leading to side reactions, solvent evaporation, and gas evolution. A practical electrolyte must function reliably across a broad temperature range, ensuring stable performance in diverse environmental conditions.
5. Harnessing artificial intelligence for next‐generation electrolyte design. Artificial intelligence (AI), leveraging machine learning and deep learning frameworks, is emerging as a pivotal tool in electrolyte discovery for Mg batteries. Data‐driven models enable high‐throughput prediction of salt–solvent–additive combinations, uncovering non‐intuitive structure–property relationships inaccessible by conventional approaches. Accelerated screening pipelines, integrating computational chemistry and experimental feedback, offer pathways to rationally design stable, high‐performance electrolytes. Future advancements will depend on expanding high‐quality datasets, refining model interpretability, and tightly coupling AI‐guided insights with interfacial characterization. AI‐driven electrolyte engineering holds the potential to redefine discovery paradigms and accelerate the deployment of practical Mg battery storage technologies.
6. Electrolyte design aligned with cathode chemistries. Advanced cathodes for Mg batteries encompass high‐voltage intercalation–type oxides (e.g., spinels, polyanionic compounds), conversion–type sulfur‐based cathodes (e.g., S, Cu_2_S, CuS), and organic redox–active cathodes (e.g., PTO, COFs). Effective electrolyte optimization must consider the specific electrochemical and interfacial demands of these cathode classes. For high‐voltage intercalation–type cathodes, electrolytes require wide electrochemical stability windows and minimal oxidative decomposition to preserve interfacial integrity and suppress passivation. Chlorine‐containing electrolytes, while facilitating reversible Mg plating/stripping, may trigger side reactions or corrosion at high‐voltage cathode interfaces. Conversely, chlorine‐free electrolytes, although chemically more stable, often exhibit limited Mg^2+^ transport kinetics and interfacial instability, which can reduce capacity and cycling efficiency on high‐voltage cathodes. Conversion–type sulfur‐based cathodes face challenges such as polysulfide dissolution and shuttle effects. In these systems, chlorine‐containing electrolytes may react with dissolved polysulfides or catalyze parasitic reactions at the Mg anode, whereas chlorine‐free electrolytes often fail to stabilize polysulfides and may display high overpotentials during Mg deposition. Addressing these challenges requires careful tuning of solvation structures, incorporation of redox–mediating additives, and interfacial engineering to enable uniform Mg deposition and mitigate shuttle effects. Organic redox–active cathodes, valued for their structural tunability and sustainability, introduce solubility and stability issues. Chlorine‐containing electrolytes may promote undesirable halide‐mediated reactions, while chlorine‐free systems must overcome solubility‐driven capacity fading and interface incompatibilities. Design strategies include molecular modification of redox–active sites, incorporation of insoluble frameworks, and optimization of co‐solvents or additives to stabilize the cathode–electrolyte interface. Thus, aligning electrolyte composition with the chemical and electrochemical requirements of each advanced cathode type is essential for achieving high‐efficiency and long‐lasting Mg batteries.

## Conflict of Interest

The authors declare no conflict of interest.
